# Light- and chemical-induced ciliary signaling governs dorsal/ventral regionalization of human telencephalic organoids

**DOI:** 10.1038/s41467-026-73505-2

**Published:** 2026-05-22

**Authors:** Issei S. Shimada, Akari Goto, Yutaka Hashimoto, Hajime Inoue, Takuto Sugawara, Tomohiro Doura, Tsubasa Fujita, Toshiaki Iwata, Riko Shimmoto, Hiroshi Takase, Masayuki Itoh, Shigeki Kiyonaka, Yoichi Kato

**Affiliations:** 1 Department of Cell Biology, Graduate School of Medical Sciences, Nagoya City University, Nagoya, Aichi Japan; 2 Department of Biomolecular Engineering, Graduate School of Engineering, Nagoya University, Nagoya, Aichi Japan; 3 Core Laboratory, Graduate School of Medical Sciences, Nagoya City University, Nagoya, Aichi Japan; 4 Department of Biochemistry and Cellular Biology, National Center of Neurology and Psychiatry, Kodaira, Tokyo Japan; 5 Research Institute for Quantum and Chemical Innovation, Institutes of Innovation for Future Society, Nagoya University, Nagoya, Aichi Japan

**Keywords:** Neural stem cells, Ciliogenesis, Neural patterning

## Abstract

Neural stem/progenitor cells (NPCs) have primary cilia, which are critical organelles for Sonic hedgehog signaling. However, little is known about the components of primary cilia in NPCs and whether manipulating signaling in the cilia is sufficient to alter dorsal/ventral regional identity. Using a human telencephalic organoid model, we perform comprehensive proteomic profiling of NPC cilia and find enrichment in GTPase signaling. Deletion of the ciliary GTPase *ARL13B* reduces ciliary localization of GPR161, an orphan G protein-coupled receptor 161 that negatively regulates Sonic hedgehog, resulting in ventralization of NPCs. *GPR161* deletion also induces ventralization. To investigate whether manipulation of ciliary signaling is sufficient to restore dorsal identity in this context, we optogenetically elevate ciliary cAMP, rescuing dorsal fate in *GPR161* KO organoids. Furthermore, chemogenetic induction of GPR161 removal from cilia is sufficient to increase ventral NPCs. These data indicate that ciliary signaling functions as a critical switch regulating dorsal/ventral fate decisions.

## Abstract (web summary)

Brain regionalization is a critical process for human brain development. Here, Shimada et al. show that the primary cilium, a hair-like projection on the cell surface, helps determine regional identity in human brain organoids. Altering signaling in the cilium is sufficient to change neural progenitor cell fate.

## Introduction

Primary cilia are antenna-like organelles with various biological functions, including roles in cancer and development^1^. Primary cilia function as antennae, hubs for post-translational regulation of various transcription factors, synapse-receiving organelles, and specialized cAMP signaling switches, highlighting their diverse roles in cellular signaling^1,2^. To function as antennae for extracellular signaling, primary cilia contain ion channels, more than 30 G protein-coupled receptors (GPCRs), adenylyl cyclases 3, 5, and 6, and PKA (protein kinase A) holoenzymes and substrates^3–5^. Importantly, a large fraction of neural stem/progenitor cells (NPCs) have primary cilia that protrude into the ventricle in the cortex^6^. Ciliary dysfunction leads to various brain abnormalities, such as neural tube defects, Joubert syndrome, Dandy-Walker syndrome, and Bardet-Biedl syndrome, which are commonly classified as ciliopathies^4,7^. However, little is known about which ciliary proteins are critical for regulating NPCs.

Sonic hedgehog (SHH) signaling is one of the major signal transduction pathways involved in functions of primary cilia. When SHH is absent, Patched1 (PTCH1) suppresses the SHH signaling pathway activity by lowering accessible sterols in the ciliary membrane, which keeps Smoothened (SMO) activity low^8^. In parallel, a ciliary orphan G protein-coupled receptor 161 (GPR161) constitutively drives Gα_s_/cAMP/PKA signaling, resulting in cleavage of full-length GLI3 into the repressor form (GLI-repressor)^9,10^. These processes lead to transcriptional inhibition of the SHH signaling pathway^11^. When SHH binds PTCH1, PTCH1 exits the cilium, ciliary sterol accessibility increases, and sterol-bound SMO becomes activated. Activated SMO inhibits PKA directly via its C-terminal PKA inhibitor motif^12^. GPR161 is also removed from cilia by a GRK2/β-arrestin/BBSome-dependent process^11,13^. These mechanisms reduce GLI3 repressor formation and induce GLI activation. GLI1 and GLI2 (GLI-activator) function as amplifiers and transcription factors of the SHH signaling^14^, and activated GLI1 and GLI2 translocate to the nucleus to activate SHH target genes. These spatial post-translational mechanisms determine the strength of SHH signaling.

During the embryonic stage, SHH signaling is critical for neural tube development^15^. SHH is secreted from the notochord and floor plate of the neural tube, and promotes ventral NPC identity^16^. On the other hand, WNT and BMP are secreted from the dorsal region of the neural tube and promote dorsal NPC identity^17^. Disruption of SHH signaling induces altered dorsal/ventral patterning in neural tube formation in mice^18^. Deletion of *Ptch1* or *Gpr161* increases Shh signaling and induces ventralization of the neural tube with expanded domains of Foxa2-, Nkx2.2-, and Olig2-positive NPCs, and decreased domains of Pax7- and Pax6-positive NPCs^15,16^. Shh is also secreted from the hypothalamus region. In the tripartite hypothalamus model, the hypothalamus is positioned ventral to the telencephalon and two parts of the diencephalon (prethalamus and zona limitans intrathalamica) during development^19^. Therefore, Shh is a critical morphogen for brain regionalization during development.

Brain regionalization is a critical process for human brain development. Although general characteristics of brain function and development have been studied in various animal models, it remains largely unclear what determines human brain architecture^20–22^. Brain organoids are an in vitro model of human brain generated from pluripotent stem cells^23–25^. Previous studies showed the existence of primary cilia in brain organoids and deletion of genes encoding ciliary components disrupted dorsal/ventral patterning of NPCs^24,26–28^. Schembs et al. demonstrated that loss of Inositol polyphosphate-5-phosphatase E (INPP5E) induced cortical organoids with ventral telencephalic identity and upregulation of SHH signaling^26^. Others also showed that engineered SHH secretion regulates dorsal/ventral patterning of NPCs in various brain organoid models^29–31^. Consistent with this, a morphogen-gradient patterning study showed that graded SHH is critical for dorsal/ventral patterning of brain organoids^32^. These studies demonstrated the critical role of SHH and primary cilia in NPCs of brain organoids. Despite these advances, little is known about the molecular composition of cilia in human NPCs and whether manipulating components in primary cilia affects the fate of NPCs.

Here, we use proximity labeling to define the ciliary proteome of human NPCs of telencephalic organoids and reveal an enrichment of GTPase proteins. Deletion of *ADP-Ribosylation Factor-Like Protein 13B* (*ARL13B*), a ciliary GTPase, and deletion of its downstream target *GPR161* induces ventralization of NPCs in the organoids. We elevate ciliary cAMP levels by optogenetics to restore dorsal identity of *GPR161* KO organoids. Lastly, we generate a chemogenetic model to regulate the exit of GPR161 from the cilia to induce ventralization of NPCs in the organoids. These data indicate that ciliary components serve as an instructive switch governing dorsal/ventral fate decisions in human NPCs.

## Results

### Cilia in telencephalic organoids are signaling hubs

**Fig. 1:**
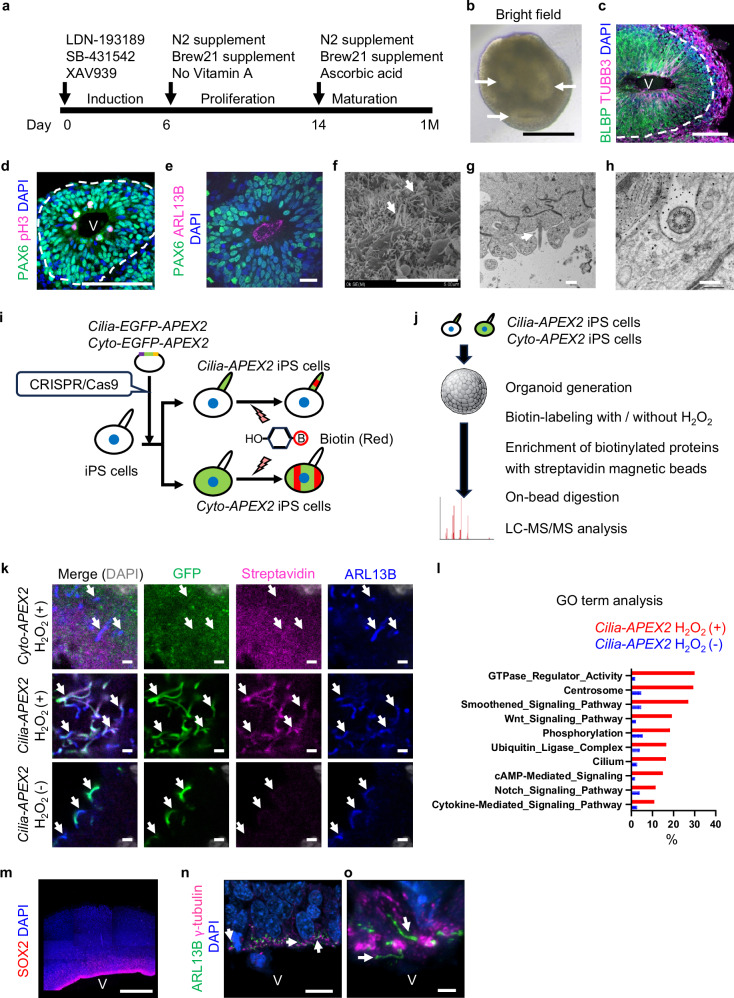
Proximity labeling identifies enriched ciliary signaling proteins in primary cilia of neural stem/progenitor cells (NPCs) in telencephalic organoids. **a** A scheme of telencephalic organoid generation from iPS cells. M indicates months. **b** A bright-field view of a human telencephalic organoid. Arrows indicate ventricles. **c** BLBP-positive NPCs and β-tubulin III (TUBB3)-positive neurons. The white dotted line indicates the junction between the ventricular zone and the cortical plate. V ventricle. **d** pH3-positive mitotic cells in the inner layer of PAX6-positive NPCs enriched in the ventricular zone. The white dotted line indicates a ventricular zone. **e** ARL13B-positive primary cilia were observed inside the ventricular zone enriched in PAX6-positive NPCs. **f** Scanning electron microscopy (SEM) analysis revealed a primary cilia-enriched region inside the ventricular zone of telencephalic organoids. Arrows indicate representative primary cilia. **g** Transmission electron microscopy (TEM) analysis showed one primary cilium per NPC in the ventricular zone. The arrow indicates a primary cilium. **h** TEM analysis showed that the primary cilium has a 9 + 0 structure. **i** CRISPR/Cas9 was used to insert *cyto-EGFP-APEX2* and *cilia-EGFP-APEX2* into the AAVS1 locus in iPS cells. Following treatment with biotin-phenol and H_2_O_2_, biotin-labeled proteins can be visualized with a streptavidin-conjugated fluorophore in primary cilia. **j** Telencephalic organoids were generated from *cyto-EGFP-APEX2* iPS cells and *cilia-EGFP-APEX2* iPS cells. At 2 weeks of culture, telencephalic organoids were treated with biotin-phenol and H_2_O_2_. Each sample from 200 to 500 organoids. Biotin-labeled proteins were enriched and identified by mass spectrometry. **k** Organoids were treated with or without H_2_O_2_ and stained for ARL13B (primary cilia), GFP (EGFP-APEX2), and streptavidin (biotinylated proteins). Arrows indicate representative primary cilia. **l** Ciliary proteins identified by mass spectrometry analysis and GO term analysis. Red bars indicate proteins enriched in H_2_O_2_-treated samples. Blue bars indicate proteins in samples without H_2_O_2_ treatment. **m**–**o** SOX2-positive NPCs were observed in the cortex of a human fetus at 15 gestational weeks. Source data are provided as a Source Data file. Scale bars: **b** 500 µm, **c**, **d** 100 µm, **e** 20 µm, **f** 5 µm, **g** 500 nm, **h** 200 nm. **k** 1 µm, **m** 1000 µm, **n** 10 µm, and **o** 2 µm. Illustration from NIAID NIH BioArt Source (bioart.niaid.nih.gov/bioart/399 and bioart.niaid.nih.gov/bioart/582).

NPCs, also known as apical radial glia, possess primary cilia that are observed in humans, mice, and brain organoid models, but the functions and components of primary cilia in NPC regulation are not well known^26,27^. To examine the role of primary cilia in human NPCs, we generated induced pluripotent stem (iPS) cell-derived telencephalic organoids using a guided differentiation protocol^33^ (Fig. 1a). After one month of culture, we successfully generated dorsal telencephalic organoids containing multiple ventricular zones (Fig. 1b). These ventricular zones were composed of BLBP-positive NPCs surrounded by β-tubulin III-positive neurons (Fig. 1c and Supplementary Fig. 1a). As in the mouse brain, phosphorylated histone H3 (pH3)-positive mitotic cells were observed at the inner surface of PAX6-positive ventricular zones (Fig. 1d and Supplementary Fig. 1b). Electron microscopy analysis also revealed accumulation of mitotic cells around the ventricular zones (Supplementary Fig. 1c). Primary cilia stained for ARL13B were observed in the ventricles surrounded by PAX6-positive ventricular zones of telencephalic organoids (Fig. 1e and Supplementary Fig. 1d). We next performed scanning electron microscopy (SEM) to visualize primary cilia in the ventricular zone. We observed primary cilia pointing toward the ventricles from cells in the ventricular zone (Fig. 1f and Supplementary Fig. 1e, f). In transmission electron microscopy (TEM) analysis, we clearly observed that each apical endfoot of NPCs contained one primary cilium (Fig. 1g and Supplementary Fig. 1g). We did not observe any NPCs containing more than one primary cilium. Each NPC apical endfoot contained one mother centriole and one daughter centriole (Supplementary Fig. 1h, i). The electron micrograph of the cross-section of primary cilia showed a 9 + 0 axoneme structure (Fig. 1h), which indicates that NPCs have primary cilia in telencephalic organoids.

To systematically identify proteins localized in primary cilia of telencephalic organoids, we utilized a proximity labeling technique followed by unbiased protein identification using mass spectrometry^34^. We generated iPS cells expressing cytoplasm-localized APEX2 (*cyto-APEX2* iPS cells) and cilia-localized APEX2 (*cilia-APEX2 iPS* cells) by inserting a *CAG-EGFP-APEX2* construct and a *CAG-NPHP3*_*1–203*_*-EGFP-APEX2* construct, respectively, into the AAVS1 safe-harbor locus of chromosome 19, using the CRISPR/Cas9 system (Fig. 1i). Cultured *EGFP*-expressing iPS cells were sorted to enrich for GFP-positive iPS cells for subsequent analysis. Using these *cyto-APEX2* and *cilia-APEX2* iPS cells, we generated telencephalic organoids (Fig. 1j). We treated them with biotin-phenol and H_2_O_2_ to label proteins in close proximity to APEX2 in the cells and cilia (*cyto-APEX2* samples treated with H_2_O_2_, *cilia-APEX2* samples treated with H_2_O_2_, and *cilia-APEX2* samples without H_2_O_2_ treatment), and then examined the localization of GFP and biotinylated proteins in the telencephalic organoids. As expected, GFP- and biotin-labeled proteins were enriched in ARL13B-positive primary cilia of the NPCs in the *cilia-APEX2* telencephalic organoids treated with H_2_O_2_, but not in *cyto-APEX2* telencephalic organoids treated with H_2_O_2_ or *cilia-APEX2* telencephalic organoids without H_2_O_2_ treatment (Fig. 1k). To isolate proteins from these primary cilia, we enriched biotin-labeled proteins using streptavidin magnetic beads and performed unbiased protein identification using mass spectrometry. We identified commonly well-known ciliary proteins (Supplementary Fig. 1j and Supplementary Data 1). We conducted a systematic Gene Ontology (GO) term analysis and found various cilia-related pathways and diseases in proteins derived from *cilia-APEX2* telencephalic organoids treated with H_2_O_2_ (Supplementary Fig. 2a–c). Cilia-related GO analysis, including “Cilium”, “Centrosome”, “Smoothened signaling components” and “GTPase regulator activity molecules” revealed that various signaling pathways and cilium-related components were enriched in H_2_O_2_-treated *cilia-APEX2* samples (Fig. 1l), indicating that we successfully identified proteins in the primary cilia. These data indicate that primary cilia are enriched in signaling components in NPCs of human telencephalic organoids^35^.

We then assessed primary cilium localization in the human fetal cortex. ARL13B-positive primary cilia extended from SOX2-positive NPCs in the ventricular zone of the cortex in a human fetus at 15 gestational weeks (Fig. 1m–o). The primary cilium was nucleated from a γ-tubulin-positive basal body. These data indicate that NPCs in the fetal cortex have primary cilia, as previously reported in the forebrain of a postconception week 8 human embryo and in other mammals^26,36,37^.

### *ARL13B*-deleted NPCs have abnormal primary cilia

**Fig. 2:**
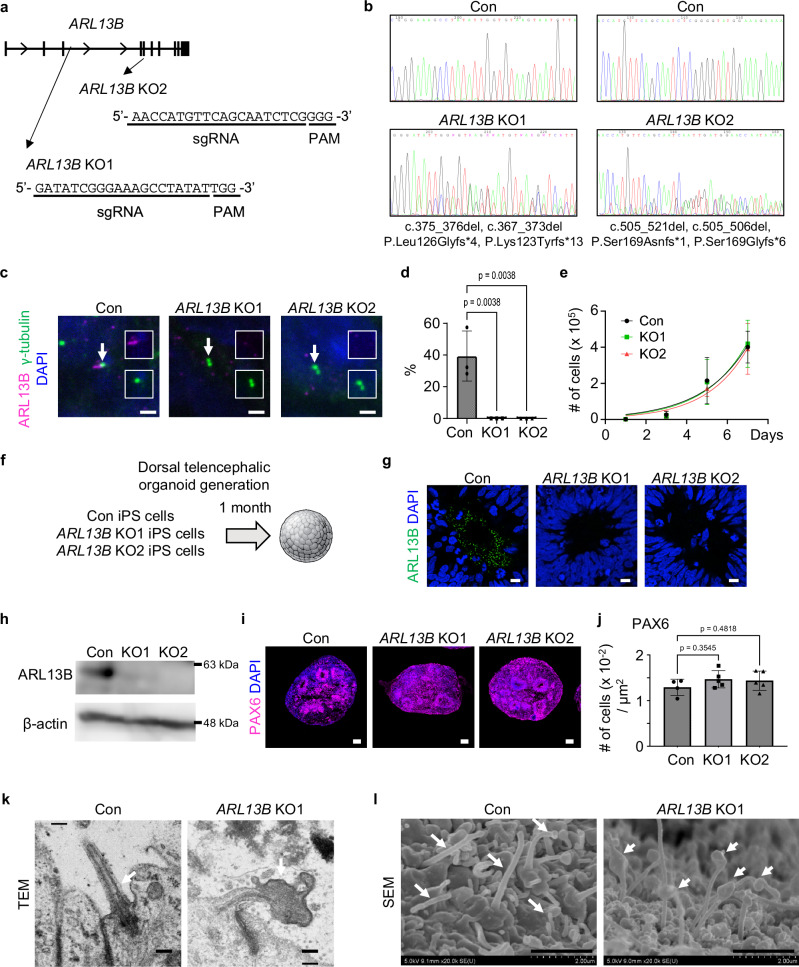
Deletion of *ARL13B* induces disrupted morphology of primary cilia in neural stem/progenitor cells (NPCs) in telencephalic organoids. **a** A scheme of *ARL13B* gene with CRISPR/Cas9 target sequences. **b** Sanger sequencing of CRISPR/Cas9-targeted loci in control (Con; 201B7 iPS cells (left) and Windy iPS cells (right)), *ARL13B* KO1 (201B7 iPS cells), and *ARL13B* KO2 iPS cells (201B7 iPS cells). **c** ARL13B-positive primary cilia were evident in control, but not *ARL13B* KO1 or *ARL13B* KO2 iPS cells. γ-tubulin indicates basal bodies. Arrows indicate basal bodies. **d** Quantification of ARL13B-positive primary cilia in (**c**). *n* = 3 technical replicates in three independent experiments. One-way ANOVA with Sidak’s test was performed. **e** Cell growth curves of control, *ARL13B* KO1, and *ARL13B* KO2 iPS cells. *n* = 3 technical replicates in three independent experiments. Two-way ANOVA and Dunnett’s multiple comparisons test were performed. *p* = 0.9992 (control vs. KO1) and *p* = 0.8887 (control vs. KO2). **f** Generation of telencephalic organoids from iPS cells. **g** ARL13B-positive primary cilia were observed in the ventricular zones (VZ) of control, but not in *ARL13B* KO1 or *ARL13B* KO2 telencephalic organoids. **h** Western blot analysis revealed that ARL13B expression was absent in *ARL13B* KO1 and *ARL13B* KO2 organoids. β-actin is a loading control. **i** PAX6-positive NPCs were observed in the VZ of control, *ARL13B* KO1 and *ARL13B* KO2 organoids. **j** The number of PAX6-positive NPCs in the VZ of control, *ARL13B* KO1, and *ARL13B* KO2 organoids. *n* = 4, 5, 5 organoids in three independent experiments. One-way ANOVA with Sidak’s test was performed. **k** Transmission electron microscopy (TEM) analysis revealed the existence of bulged primary cilia in the VZ of *ARL13B* KO1 organoids. Arrows indicate representative primary cilia. **l** Scanning electron microscopy (SEM) analysis revealed the existence of bulged and shortened primary cilia in the VZ of *ARL13B* KO1 organoids. Arrows indicate representative primary cilia. Source data are provided as a Source Data file. Scale bars: **c** 2 µm, **g** 10 µm, **i** 100 µm, **k** 200 nm, and **l** 2 µm. **d**, **e**, and **j** Data are presented as mean values ± standard deviation. Illustration from NIAID NIH BioArt Source (bioart.niaid.nih.gov/bioart/399).

ARL13B is a small GTPase enriched in primary cilia, and contributes to cilia morphology and signaling^36^. To examine the role of ciliary signaling in NPCs in telencephalic organoids, we generated *ARL13B* knockout (KO) iPS cells using the CRISPR/Cas9 system (Fig. 2a, b and Supplementary Fig. 3a). Deletion of *ARL13B* in iPS cells was confirmed by immunocytochemistry (Fig. 2c, d). Deletion of *ARL13B* did not affect cell growth (Fig. 2e) or stem cell marker expression (Supplementary Fig. 3b) in iPS cells compared to control iPS cells. These data indicate that ARL13B may be dispensable for iPS cell maintenance. To examine the function of ciliary signaling in NPCs, we generated telencephalic organoids derived from *ARL13B* KO iPS cells (hereafter *ARL13B* KO organoids) using a guided differentiation protocol toward dorsal telencephalic identity (Figs. 1a and 2f). We first confirmed that ARL13B protein was absent in *ARL13B* KO organoids at 1 month of culture (Fig. 2g, h and Supplementary Fig. 3c). Deletion of *ARL13B* did not disrupt generation of PAX6-positive NPCs in the organoids, compared to control telencephalic organoids (Fig. 2i, j and Supplementary Fig. 3d).

We next analyzed the morphology of primary cilia in *ARL13B* KO NPCs. We cultured iPS cell-derived NPCs under 2D conditions and quantified the number and length of primary cilia. The number of ciliated cells was significantly decreased in *ARL13B* KO NPCs in 2D culture (Supplementary Fig. 3e, f). The length of cilia was significantly shorter in *ARL13B* KO NPCs than in control cells (Supplementary Fig. 3g).

To further analyze primary cilia in *ARL13B* KO organoids, we employed electron microscopy. Primary cilia were easily visualized in the ventricles of control telencephalic organoids (Fig. 2k). In some cases, we identified primary cilia whose tips were bulged in the *ARL13B* KO NPCs (Fig. 2k). Microtubule-like structures accumulated at the tips of primary cilia in *ARL13B* KO NPCs, suggesting disrupted cargo trafficking. SEM analysis revealed an enrichment of bulged primary cilia in ventricles of *ARL13B* KO organoids (Fig. 2l). These data indicate that *ARL13B* deletion disrupted morphology of primary cilia in NPCs of telencephalic organoids.

### *ARL13B* deletion induces ventralization and SHH activation

**Fig. 3:**
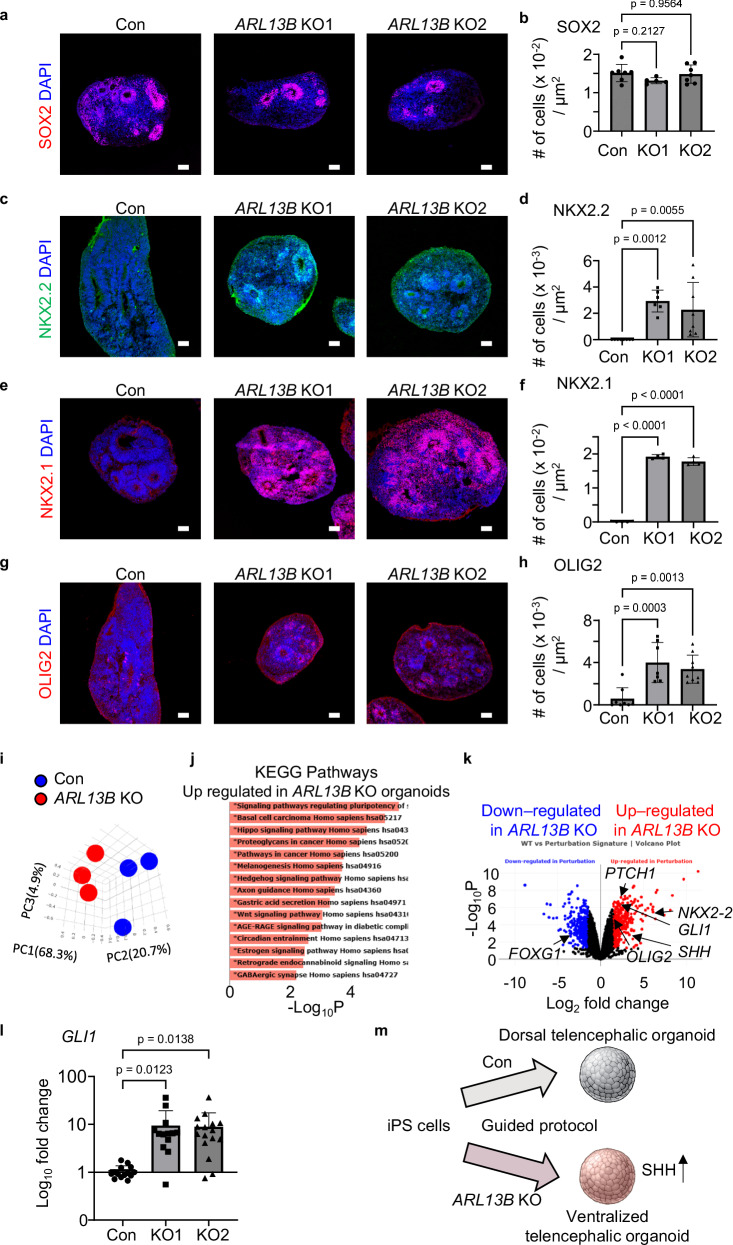
*ARL13B* deletion induces ventralization in telencephalic organoids. **a**–**h** Neural stem/progenitor cells (NPCs) were observed in the ventricular zones (VZ) of *ARL13B* KO1 and *ARL13B* KO2 organoids. **a**, **b** SOX2-positive NPCs. *n* = 7, 5, 7 organoids in two independent experiments. **c**, **d** NKX2.2-positive ventralized NPCs. *n* = 8, 6, 8 organoids in three independent experiments. **e**, **f** NKX2.1-positive ventralized NPCs. *n* = 3, 4, 4 organoids in one independent experiment. **g**, **h** OLIG2-positive ventralized NPCs. *n* = 8, 7, 9 organoids in three independent experiments. **i** PCA showing that control and *ARL13B* KO1 organoid transcripts have distinct gene expression patterns. Numbers indicate the variance explained. Each sample contains pooled RNA from 10 to 30 telencephalic organoids at 2 weeks. **j** KEGG pathway analysis revealed that various signaling pathways were upregulated. KEGG pathway enrichment analysis was performed using BioJupies. The *p* value was calculated with Fisher’s exact test and adjusted for multiple testing using the Benjamini–Hochberg method. Bold pathway names indicate false discovery rate (FDR) < 0.1. **k** Volcano plot of RNA-seq between control and *ARL13B* KO1 organoids analyzed by BioJupies. Genes with log_2_ fold change greater than 1.5 and raw *p*-values less than 0.05, and genes with log_2_ fold change less than −1.5 and raw *p*-values less than 0.05 are colored red and blue, respectively. Statistical significance was assessed using two-sided moderated *t*-tests, and *p*-values were adjusted for multiple comparisons using the Benjamini–Hochberg method. *PTCH1*, *NKX2-2*, *GLI1*, and *SHH*, which are components of SHH signaling, are indicated by black arrows. **l** qRT-PCR revealed altered expression of SHH target gene in *ARL13B* KO1 and *ARL13B* KO2 organoids. Each dot represents RNAs pooled from 10 to 30 telencephalic organoids. *n* = 14, 13, 16 pools of telencephalic organoids in five independent experiments. **m** A summary of the *ARL13B* deletion effects on the telencephalic organoids. Source data are provided as a Source Data file. **b**, **d**, **f**, **h**, and **l** One-way ANOVA with Sidak’s test was performed. Data are presented as mean values ± standard deviation. Scale bars: **a**, **c**, **e**, **g** 100 µm. Illustration from NIAID NIH BioArt Source (bioart.niaid.nih.gov/bioart/399).

ARL13B is a critical component of various ciliary signaling pathways in a context-dependent manner^38^. We examined the effects of *ARL13B* deletion on telencephalic organoids. Almost all cells in the ventricular zones expressed SOX2 and FOXG1, indicating that cells in the ventricular zones were telencephalic NPCs (Fig. 3a, b and Supplementary Fig. 4a–c). Interestingly, *ARL13B* KO organoids contained NKX2.2-positive NPCs, indicating the appearance of ventral NPCs (Fig. 3c, d and Supplementary Fig. 4d). This result was unexpected because we employed the guided differentiation protocol to generate dorsal telencephalic organoids using dual SMAD inhibitors^33^. We also observed increased numbers of NKX2.1-positive NPCs, GSX2-positive NPCs, and OLIG2-positive NPCs, but not FOXA2-positive NPCs in *ARL13B* KO organoids compared to control telencephalic organoids, indicating ventral induction by *ARL13B* deletion (Fig. 3e–h and Supplementary Fig. 4e–i).

To examine downstream mechanisms of *ARL13B* deletion on NPCs, we isolated RNA from organoids enriched for NPCs prior to the neural maturation period, and performed RNA-seq analysis (Supplementary Data 2). Principal component analysis (PCA) revealed that *ARL13B* deletion affected the gene expression patterns of telencephalic organoids (Fig. 3i). KEGG pathway analysis, volcano plot analysis and heatmap analysis revealed upregulation of the SHH signaling pathway among various other pathways (Fig. 3j, k and Supplementary Fig. 5a). Furthermore, qRT-PCR analysis confirmed that *ARL13B* deletion significantly upregulated expression of *GLI1* (Fig. 3l). RNA-seq analysis revealed that *ARL13B* deletion reduced expression of cortical and telencephalic markers such as *EMX2* and *FOXG1*, but no obvious effects on markers of midbrain and hindbrain, such as *IRX2*, *IRX3*, and *HOXA2* (Supplementary Fig. 5b). RNA-seq analysis also revealed that *ARL13B* deletion induced increased ventral markers such as *SIX3*, *NKX2-1*, and *NKX2-2* (Supplementary Fig. 5c). Since cells in the ventricular zones mainly expressed FOXG1, PAX6, GSX2, NKX2.1, and NKX2.2 and did not express FOXA2, the loss of *ARL13B* induced ventralization of the telencephalic organoids (Fig. 3m).

### GPR161 negatively regulates SHH signaling in NPCs

**Fig. 4:**
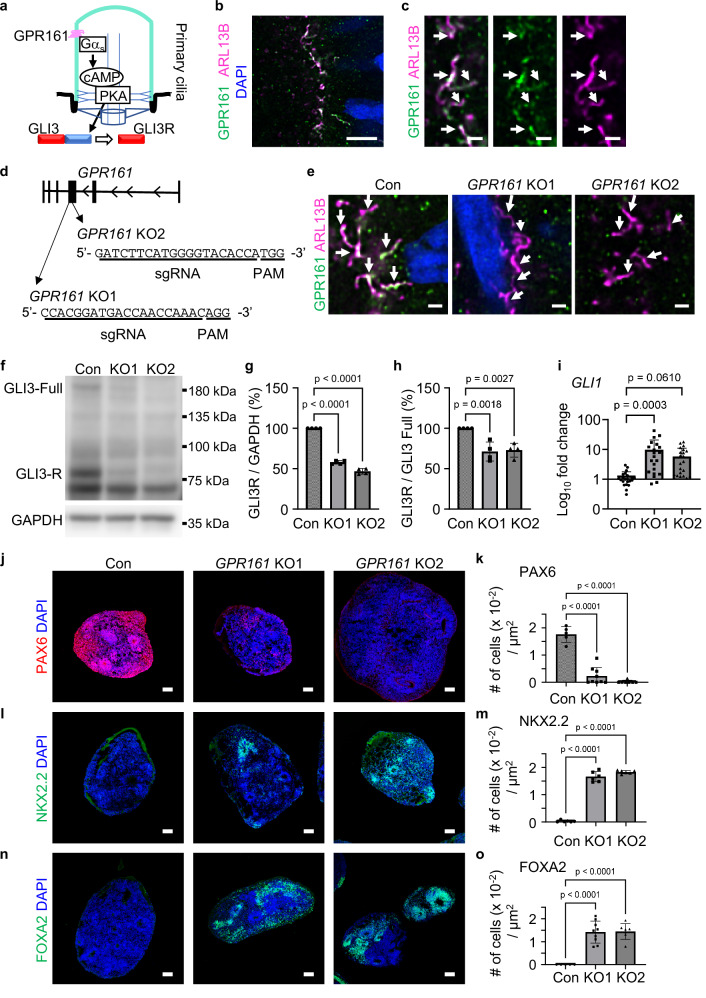
Deletion of *GPR161* induces ventralization of neural stem/progenitor cells via GLI3 regulation. **a** A diagram showing GPR161 functions in primary cilia. GLI3, full-length GLI3; GLI3R, GLI3 repressor. **b**, **c** GPR161 localization in ARL13B-positive primary cilia in the ventricular zone of control telencephalic organoids. Arrows indicate GPR161 in primary cilia. **d** A scheme of *GPR161* gene with CRISPR/Cas9 target sequences. **e** GPR161 localization in ARL13B-positive primary cilia was observed in the ventricular zones of control, but not in *GPR161* KO1 or KO2 organoids. Arrowheads indicate primary cilia; arrows indicate GPR161 in primary cilia. DAPI is shown in blue. **f**–**h** Western blotting showed reduction of the GLI3 repressor (GLI3R) in *GPR161* KO organoids. GAPDH is a loading control. *n* = 4, 4, 4 pools of organoids in two independent experiments. One-way ANOVA with Sidak’s test was performed. **i** qRT-PCR revealed altered expression of SHH target genes in *GPR161* KO1 and *GPR161* KO2 organoids during the proliferation period. Each dot indicates RNAs pooled from 10–30 telencephalic organoids. *n* = 22, 21, 22 pools of organoids in five independent experiments. One-way ANOVA with Sidak’s test was performed. **j**, **k** Reduced PAX6-positive dorsal neural stem/progenitor cells (NPCs) in the ventricular zones of *GPR161* KO1 and *GPR161* KO2 organoids. *n* = 5, 9, 7 organoids in two independent experiments. One-way ANOVA with Sidak’s test was performed. **l**, **m** Increased NKX2.2-positive ventral NPCs in the ventricular zones of *GPR161* KO1 and *GPR161* KO2 organoids. *n* = 6, 6, 6 organoids in one independent experiment. One-way ANOVA with Sidak’s test was performed. **n**, **o** Increased FOXA2-positive ventral NPCs in the ventricular zones of *GPR161* KO1 and *GPR161* KO2 organoids. *n* = 5, 9, 7 telencephalic organoids in two independent experiments. One-way ANOVA with Sidak’s test was performed. Source data are provided as a Source Data file. **g**, **h**, **i**, **k**, **m**, and **o** Data are presented as mean values ± standard deviation. Scale bars: **b** 5 µm and **c**, **e** 1 µm and **j**, **l**, **n** 100 µm.

We hypothesized that cilia-intrinsic mechanisms are critical for the ventralization of *ARL13B* KO NPCs. GPR161 localizes to primary cilia in both mouse and human telencephalic organoids, and negatively regulates the SHH signaling pathway (Fig. 4a)^10,39^. Previous studies showed that deletion of *ARL13B* causes GPR161 mislocalization and impairs responsiveness to SHH signaling in primary cilia^40,41^. We observed that GPR161 localized to primary cilia of NPCs of control telencephalic organoids (Fig. 4b, c). Percentages of GPR161-positive cilia were significantly decreased in *ARL13B* KO NPCs under 2D culture (Supplementary Fig. 6a). Thus, it is possible that GPR161 underlies *ARL13B* deletion induced upregulation of SHH signaling.

To examine the role of GPR161 in telencephalic organoids, we generated *GPR161* KO iPS cells using CRISPR/Cas9 (Fig. 4d and Supplementary Fig. 6b). Growth of *GPR161* KO iPS cells was similar to that of control iPS cells (Supplementary Fig. 6c). Deletion of *GPR161* did not affect expression of stem cell markers in iPS cells (Supplementary Fig. 6d), suggesting that GPR161 may not be critical for iPS cell maintenance.

To examine the function of GPR161-mediated signaling in NPCs, we generated telencephalic organoids using *GPR161* KO iPS cells (hereafter *GPR161* KO organoids) using the guided differentiation protocol for dorsal telencephalic organoids. We first confirmed that GPR161 was absent from primary cilia in the ventricles of *GPR161* KO organoids after 1 month of culture (Fig. 4e). To examine the effects of *GPR161* on SHH signaling, we analyzed critical components of the SHH signaling pathway, GLI1, GLI2, GLI3, and SMO. We showed that *GPR161* deletion decreased GLI3 repressor formation (Fig. 4f–h). Deletion of *GPR161* induced upregulation of *GLI1* (Fig. 4i). We did not observe an increase in GLI2 accumulation in the primary cilia of NPCs in *GPR161* KO organoids (Supplementary Fig. 6e, f). We observed increased SMO accumulation in the primary cilia of NPCs in *GPR161* KO organoids (Supplementary Fig. 6g, h). These data indicate that *GPR161* deletion shifts the GLI activator/repressor balance toward GLI activation^9^, consistent with previous reports^10,37,42^.

### *GPR161* deletion induces ventralization in NPCs

*GPR161* deletion induces open cranial and caudal neural tubes in mice and dominant-negative variants of *GPR161* have been identified in spina bifida patients, indicating the importance of GPR161 during brain development^43–45^. We examined the function of GPR161 in the formation of dorsal/ventral NPCs in telencephalic organoids. Almost all cells in the ventricular zones expressed SOX2, indicating that cells in the ventricular zones were NPCs (Supplementary Fig. 7a, b). Although we employed a method to generate dorsal telencephalic organoids using dual SMAD inhibitors^33^, *GPR161* KO organoids showed reduced numbers of PAX6-positive and PAX7-positive dorsal NPCs (Fig. 4j, k and Supplementary Fig. 7c–e). We occasionally observed OLIG2-expressing NPCs in both control and *GPR161* KO organoids (Supplementary Fig. 7f, g). We observed increased numbers of NKX2.2-positive and FOXA2-positive ventral NPCs, but not GSX2-positive NPCs in *GPR161* KO organoids, compared to control telencephalic organoids (Fig. 4l–o, Supplementary Fig. 7h, i, and Supplementary Fig. 8a, b). The analysis of previously published ChIP-seq data using brain organoids^31^ showed that GLI3 directly interacts with the NKX2.2 genomic region, indicating that the GLI3 repressor may directly inhibit *NKX2.2* expression (Supplementary Fig. 8c). These data indicate that deletion of *GPR161* prevents the formation of dorsal NPCs and induces ventral NPCs during telencephalic organoid formation using the guided differentiation protocol.

### cAMP signaling restores dorsal identity in *GPR161* KO NPCs

**Fig. 5:**
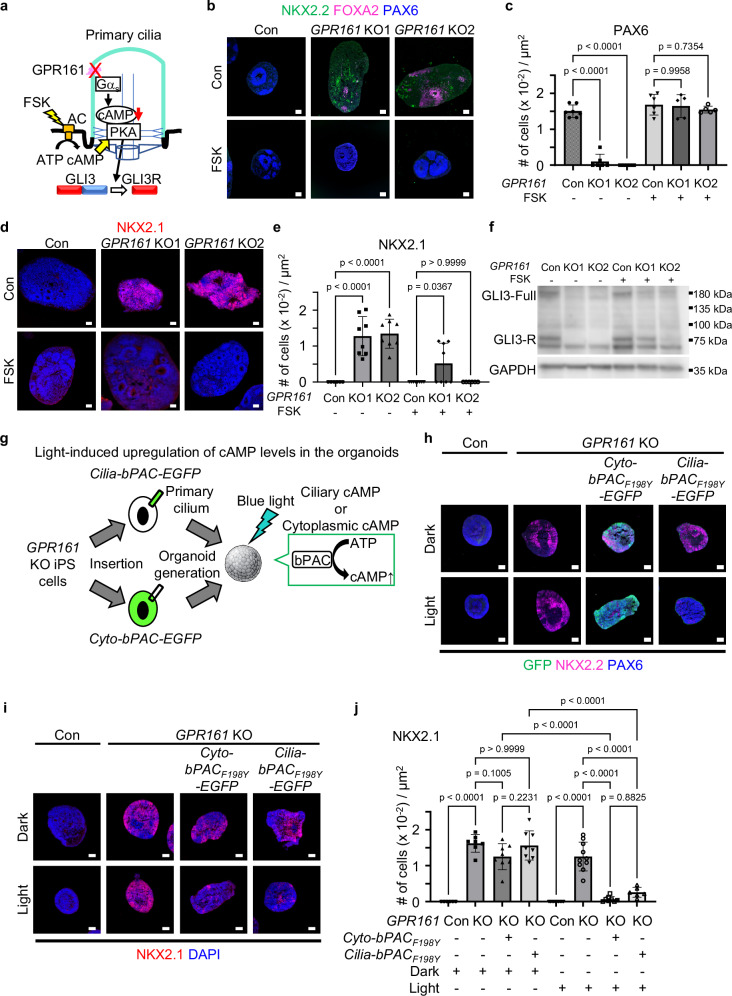
Enhancement of cytoplasmic or ciliary cAMP levels restores dorsal identity of *GPR161* KO organoids. **a** A diagram showing that forskolin treatment generates cAMP in *GPR161* KO cells. AC, adenylyl cyclase, FSK, forskolin. **b**, **c** FSK treatment restores dorsal identity of neural stem/progenitor cells (NPCs) in *GPR161* KO organoids. Analysis of PAX6-positive NPCs in the ventricular zones of telencephalic organoids. *n* = 7, 6, 6, 6, 5, 5 organoids in two independent experiments. One-way ANOVA with Sidak’s test was performed. **d**, **e** Analysis of NKX2.1-positive NPCs in the ventricular zones of telencephalic organoids. *n* = 6, 8, 8, 8, 8, 6 organoids in two independent experiments. One-way ANOVA with Sidak’s test was performed. **f** Western blot analysis revealed that the reduction of GLI3 repressor (GLI3R) in *GPR161* KO organoids was rescued by FSK treatment. GAPDH is a loading control. **g** A schematic of the optogenetic assay in *cyto-bPAC-EGFP*- and *cilia-bPAC-EGFP*-expressing *GPR161* KO iPS cells. bPAC indicates photoactivated adenylyl cyclase. **h**–**j** Enhancement of either cytoplasmic cAMP levels or ciliary cAMP levels restored dorsal identity of *cyto-bPAC*_*F198Y*_-*EGFP-* and *cilia-bPAC*_*F198Y*_-*EGFP*-expressing *GPR161* KO organoids under dark conditions or with programmed light exposure (0.1 s on, 2 min off cycle). **j** The number of NKX2.1-positive ventral NPCs was significantly decreased in the ventricular zones of both *cyto-bPAC*_*F198Y*_*-EGFP*- and *cilia-bPAC*_*F198Y*_*-EGFP*-*GPR161* KO organoids, compared to dark conditions. *n* = 6, 7, 8, 8, 10, 10, 8, 6 organoids in two independent experiments. One-way ANOVA with Sidak’s test was performed. Source data are provided as a Source Data file. **c**, **e**, and **j** Data are presented as mean values ± standard deviation. Scale bars: **b**, **d**, **h**, and **i** 100 µm. Illustration from NIAID NIH BioArt Source (bioart.niaid.nih.gov/bioart/399).

Previous studies showed that GPR161 utilized the Gα_S_-cAMP-PKA-GLI3 axis to regulate the SHH signaling pathway^10^. To examine whether cAMP-mediated signaling is critical for dorsal/ventral fate determination, we treated *GPR161* KO organoids with forskolin, a chemical that increases cAMP levels through adenylyl cyclase activation (Fig. 5a). We examined the effect of forskolin at various concentrations and durations, and found that treatment with 500 nM forskolin throughout the culture period effectively promoted dorsalization of NPCs in *GPR161* KO organoids (Supplementary Fig. 8d, e). Addition of forskolin did not affect numbers of PAX6-positive dorsal NPCs and NKX2.1-positive, NKX2.2-positive, or FOXA2-positive ventral NPCs in control telencephalic organoids (Fig. 5b–e and Supplementary Fig. 9a–d). Notably, forskolin treatment significantly rescued the number of PAX6-positive dorsal NPCs, and reduced numbers of NKX2.1-, NKX2.2-, or FOXA2-positive ventral NPCs in *GPR161* KO organoids (Fig. 5b–e and Supplementary Fig. 9a–d). Importantly, GLI3 repressor levels were restored by forskolin treatment in *GPR161* KO organoids, indicating the importance of cAMP for GLI3 repressor formation (Fig. 5f and Supplementary Fig. 9e, f).

### Ciliary cAMP restores dorsal identity of *GPR161* KO NPCs

One proposed mechanism of ciliary signaling is that cAMP regulation in cilia affects the SHH signaling pathway^2,46,47^. To examine whether an increase in ciliary or cytoplasmic cAMP is sufficient to determine the dorsal/ventral fate of NPCs in *GPR161* KO organoids, we generated cells enabling subcellular modulation of cAMP levels. We inserted a *CAG-ARL13B-photoactivated adenylyl cyclase (bPAC)-EGFP* construct into the AAVS1 locus on chromosome 19 of *GPR161* KO iPS cells to target cAMP production in the cilia (*cilia-bPAC-GPR161* KO cells). In parallel, we inserted a *CAG-bPAC-EGFP* construct to target cAMP production in the cytoplasm (*cyto-bPAC-GPR161* KO cells) (Fig. 5g and Supplementary Fig. 10a). bPAC is a light-activated adenylyl cyclase that produces cAMP upon exposure to 470 nm light. The *ARL13B-bPAC-EGFP* fusion protein enables bPAC to localize specifically to primary cilia^2^. We then generated telencephalic organoids and confirmed that bPAC-EGFP was enriched in either ARL13B-positive primary cilia or the cytoplasm of NPCs in the ventricular zones of *cilia-bPAC-EGFP*-*GPR161* KO organoids and *cyto-bPAC-EGFP*-*GPR161* KO organoids, respectively (Supplementary Fig. 10b). To examine whether ciliary cAMP-mediated signaling is sufficient for dorsal/ventral determination, *cilia-bPAC-GPR161* KO organoids and *cyto-bPAC-GPR161* KO organoids were exposed to a programmed light stimulation regimen for two weeks and were analyzed (Supplementary Fig. 10c–f). Neither dark nor light exposure affected the dorsal/ventral fate of control and *GPR161* KO organoids (Supplementary Fig. 11a–c). *Cyto-bPAC-GPR161* KO- and *cilia-bPAC-GPR161* KO organoids in dark conditions contained both PAX6- and NKX2.2-positive NPCs, indicating leaky bPAC activity in the organoids under dark conditions (Supplementary Fig. 11a–c). Notably, light exposure reduced the number of NKX2.2-positive NPCs in both *cyto-bPAC-GPR161* KO and *cilia-bPAC-GPR161* KO organoids (Supplementary Fig. 11a–c).

Since the degree of leaky bPAC activity in the dark hindered us from determining effects of cytoplasmic versus ciliary cAMP levels on dorsal/ventral brain formation, we sought to generate iPS cells expressing a low-activity variant of bPAC in the dark. A recent study showed that a point mutation (F198Y) in bPAC reduces activity in the dark^48^. Based on this, we generated *cilia-bPAC*_*F198Y*_*-GPR161* KO and *cyto-bPAC*_*F198Y*_*-GPR161* KO iPS cells. We generated telencephalic organoids and analyzed the effects of bPAC_F198Y_ on dorsal/ventral NPC formation (Supplementary Fig. 12a). Because the previous dark/light stimulation protocol affected the viability of these organoids, we shortened the duration of light exposure. Under dark conditions, c*yto-bPAC*_*F198Y*_*-GPR161* KO- and c*ilia-bPAC*_*F198Y*_*-GPR161* KO organoids exhibited reduced numbers of PAX6-positive NPCs, indicating that bPAC_F198Y_ retains low basal activity in the dark (Fig. 5h–j and Supplementary Fig. 12b). Notably, light exposure reduced the number of NKX2.1-positive and NKX2.2-positive NPCs in both *cyto-bPAC*_*F198Y*_*-GPR161* KO and *cilia-bPAC*_*F198Y*_*-GPR161* KO organoids (Fig. 5h–j and Supplementary Fig. 12c–e). Since the degree of reduction in the number of ventral NPCs between cytoplasmic and ciliary bPAC_F198Y_ activation before and after light exposure was not significantly different, these data indicate that an increase in ciliary cAMP levels following light exposure is sufficient to restore dorsal identity of *GPR161* KO organoids.

### Chemogenetic suppression of GPR161 induces ventralization

GPR161 is a constitutively active orphan GPCR and functions primarily in primary cilia^49^. Activity of GPR161 is reduced by removal from primary cilia via β−arrestin-mediated transport and endocytosis at the ciliary pocket^9,11^. We hypothesized that regulating GPR161 to promote its exit from primary cilia should suppress GPR161 signaling and induce ventralization of NPCs. However, since GPR161 is an orphan GPCR, we could not use small molecules or ligands to regulate GPR161-mediated signaling.

**Fig. 6:**
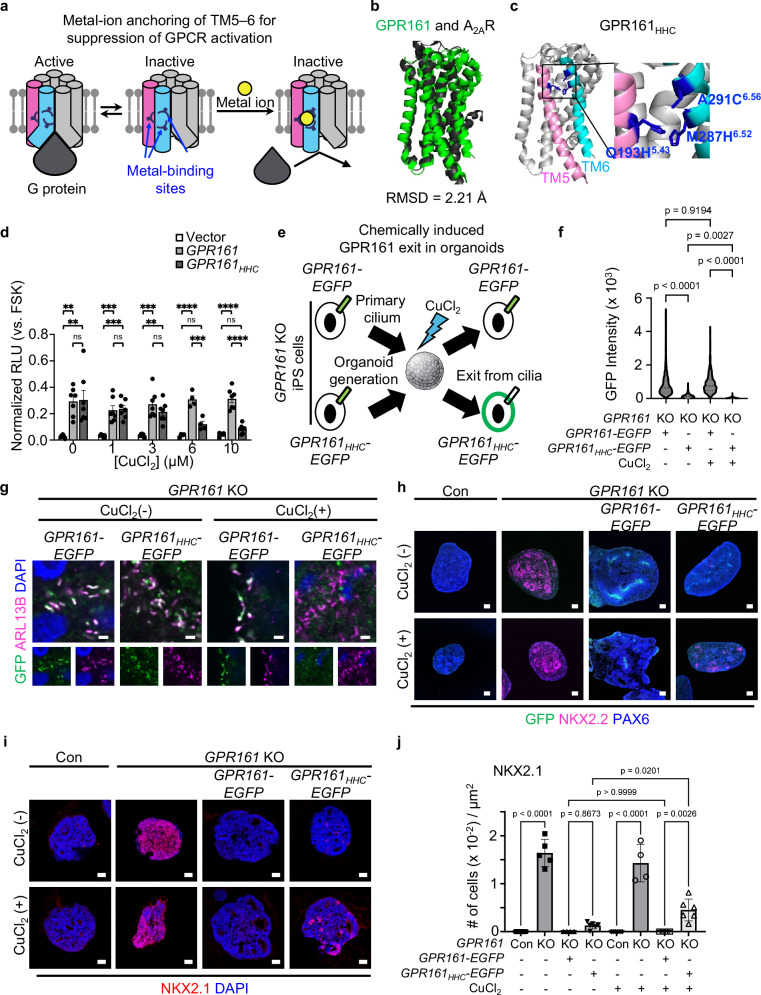
Ciliary exit of GPR161 by coordination chemogenetics induces ventralization of neural stem/progenitor cells. **a** Schematic of chemogenetic suppression of GPCR activation by metal coordination. Metal binding suppresses receptor activity independently of ligand binding. TM, transmembrane helix. **b** Structural comparison of GPR161 (green, PDB ID: 8SMV) and A_2A_R (black, PDB ID: 2YDO). RMSD, root mean square deviation. **c** Putative structures of GPR161_HHC_ mutant: GPR161 (Q193H^5.43^/M287H^6.52^/A291C^6.56^). **d** Copper(II) chloride reduced cAMP levels in *GPR161*_*HHC*_-transfected HEK293 cells. One-way ANOVA with Tukey’s multiple comparisons test was performed. At 0, 1, 3, 6, 10 µM, *n* = 6, 7, 7; 6, 7, 7; 6, 6, 6; 4, 4, 4; and 6, 7, 7 independent experiments for Mock, *GPR161*, and *GPR161*_*HHC*_, respectively. *p*-values for Mock vs *GPR161*, Mock vs *GPR161*_*HHC*,_ *GPR161* vs *GPR161*_*HHC*_ were 0.0052, 0.0039, and 0.9877 at 0 µM; 0.0003, 0.0002, and 0.9567 at 1 µM; 0.0108, 0.0658, and 0.6305 at 3 µM; <0.0001, 0.0501, and 0.0004 at 6 µM; <0.0001, 0.31, and <0.0001 at 10 µM. RLU, relative luminescence units compared to the FSK-treated control. **e** Scheme of the chemogenetic assay. Copper(II) chloride was added on day 6. Organoids were analyzed at 2 weeks. **f**, **g** Copper(II) chloride (10 µM) reduced GFP signal in primary cilia of neural stem/progenitor cells (NPCs) of *GPR161*_*HHC*_*-EGFP*-expressing *GPR161* KO organoids. *n* = 439, 601, 332, and 471 cilia i*n* two independent experiments. Violin plots show the data distribution, with horizontal lines indicating the 25th percentile, median, and 75th percentile. **h**–**j** Copper(II) chloride increased NKX2.1- and NKX2.2-positive NPCs, but not PAX6-positive NPCs, in ventricular zones of *GPR161*_*HHC*_*-EGFP*-expressing *GPR161* KO organoids. **j** *n* = 6, 5, 4, 6, 6, 4, 4, 6 organoids in two independent experiments. **f** and **j** One-way ANOVA with Sidak’s test was performed. Source data are provided as a Source Data file. Data are presented as (**d**) mean values ± standard error of the mean and (**j)** mean values ± standard deviation. **p < 0.01, ***p < 0.001 and ****p < 0.0001. Scale bars: **g** 1 µm and **h**, **i** 100 µm. Illustration from NIAID NIH BioArt Source (bioart.niaid.nih.gov/bioart/399).

Recent advances in crystal structure analysis have revealed that GPCRs undergo significant conformational changes upon agonist binding^50^. Coordination chemogenetics is a technique that uses genetically engineered mutations to introduce coordinating amino acids, allowing metal ions to bind and regulate receptor activity without impairing its intrinsic function (Fig. 6a)^51–53^. We recently demonstrated a chemogenetic method for metal ion-induced inhibition of adenosine A_2A_ receptor (A_2A_R) activity, without affecting its intrinsic ligand-induced signaling^54^. Therefore, we hypothesized that the same coordination chemogenetic strategy could be applied to suppress GPR161 activity.

To enable chemogenetic regulation of GPR161, we mimicked our published A_2A_R model, which was engineered with a single mutation (F182H) to create a metal-binding site (F182H, H250, and C254) in transmembrane helix 5 (TM5) and TM6^54^. A metal ion binds to the metal-binding site and suppresses its receptor activity independently of ligand binding^54^. Structural comparison between A_2A_R and GPR161 showed relatively high structural similarity, with a root mean square deviation (RMSD) of 2.21 Å (Fig. 6b). To apply coordination chemogenetics to GPR161, we introduced mutations (Q193H^5.43^, M287H^6.52^, and A291C^6.56^; hereafter GPR161_HHC_) that correspond to the same Ballesteros–Weinstein numbers as the coordinating residues found in the mutant of A_2A_R (Fig. 6c).

Transfection of *GPR161* and *GPR161*_*HHC*_, but not the vector control, induced similar cAMP levels in HEK293 cells in the absence of copper(II) chloride treatment (Fig. 6d). On the other hand, treatment with copper(II) chloride (6 or 10 µM) significantly reduced cAMP levels in *GPR161*_*HHC*_-transfected HEK293 cells compared to *GPR161*-transfected HEK293 cells (Fig. 6d). Copper(II) chloride treatment also significantly reduced cAMP levels in *GPR161*_*HHC*_*-EGFP*-HEK293 cells compared to *GPR161-EGFP*-transfected HEK293 cells, indicating that GFP insertion at the C-terminus did not disrupt the function of GPR161 and GPR161_HHC_ (Supplementary Fig. 13a). These results showed that *GPR161*_*HHC*_ preserves its intrinsic Gα_S_-coupled signaling while signaling is inhibited by copper(II) chloride.

To examine the functional role of a conformational change in GPR161 in NPCs, we introduced *GPR161-EGFP* or *GPR161*_*HHC*_*-EGFP* into the AAVS1 locus of chromosome 19 in *GPR161* KO iPS cells (Fig. 6e). In this manner, we could avoid the endogenous *GPR161* activity and analyze activity of the AAVS1-integrated *GPR161* constructs. We generated telencephalic organoids from these iPS cells using the guided differentiation protocol (Fig. 6e). Interestingly, ciliary localization of GPR161_HHC_-EGFP was significantly decreased as compared to GPR161-EGFP, suggesting that GPR161_HHC_-EGFP has an unknown mechanism that decreases ciliary localization (Fig. 6f, g). We treated these organoids with copper(II) chloride starting from the sixth day of differentiation and analyzed at two weeks to avoid potential toxic effects of copper(II) chloride on organoids. We first analyzed organoid size and confirmed that copper(II) chloride did not affect organoid size (Supplementary Fig. 13b, c). We analyzed GFP intensity to assess effects of copper(II) chloride on GPR161 localization. Copper(II) chloride did not affect GPR161-EGFP intensity in ARL13B-positive primary cilia of NPCs in telencephalic organoids (Fig. 6f, g). Importantly, treatment with copper(II) chloride significantly decreased GFP intensity of GPR161_HHC_-EGFP in primary cilia, compared with untreated cilia (Fig. 6f, g). These data indicate that coordination chemogenetics regulates ciliary localization of GPR161_HHC_-EGFP (Supplementary Fig. 13d).

To examine whether mislocalization of GPR161 is critical for dorsal/ventral formation of NPCs in telencephalic organoids, we analyzed these organoids with PAX6, NKX2.1, and NKX2.2. Treatment with copper(II) chloride did not affect the number of PAX6-, NKX2.1-, or NKX2.2-positive NPCs in wild-type or *GPR161* KO organoids (Fig. 6h–j, Supplementary Fig. 13e, and Supplementary Fig. 14a–c). Without copper(II) chloride treatment, insertion of *GPR161-EGFP* and *GPR161*_*HHC*_*-EGFP* rescued the phenotype of *GPR161* KO organoids, as demonstrated by increased PAX6-, and decreased NKX2.1- and NKX2.2-positive NPCs in the organoids (Fig. 6h–j, Supplementary Fig. 13e, and Supplementary Fig. 14a–c). Copper(II) chloride treatment did not affect the number of PAX6-positive NPCs in *GPR161-EGFP GPR161* KO organoids or *GPR161*_*HHC*_*-EGFP GPR161* KO organoids. Importantly, the number of NKX2.1- and NKX2.2-positive NPCs was significantly increased in *GPR161*_*HHC*_*-EGFP GPR161* KO organoids, compared to *GPR161-EGFP GPR161* KO organoids. It is worth noting that the increase in NKX2.1- and NKX2.2-positive NPCs in *GPR161*_*HHC*_*-EGFP GPR161* KO organoids following copper(II) chloride treatment remains lower than in *GPR161* KO organoids, indicating that *GPR161* KO has a stronger effect on NKX2.1 and NKX2.2 regulation than ciliary exit of GPR161_HHC_-EGFP alone. In contrast, we did not observe a difference in the number of PAX6-positive NPCs in *GPR161*_*HHC*_*-EGFP GPR161* KO organoids following copper(II) chloride treatment, indicating that the ciliary exit of GPR161_HHC_-EGFP was not sufficient to affect the number of PAX6-positive cells. Nonetheless, these data indicate that exit of GPR161_HHC_ from primary cilia was sufficient to induce mild ventralization in human telencephalic organoids.

## Discussion

**Fig. 7:**
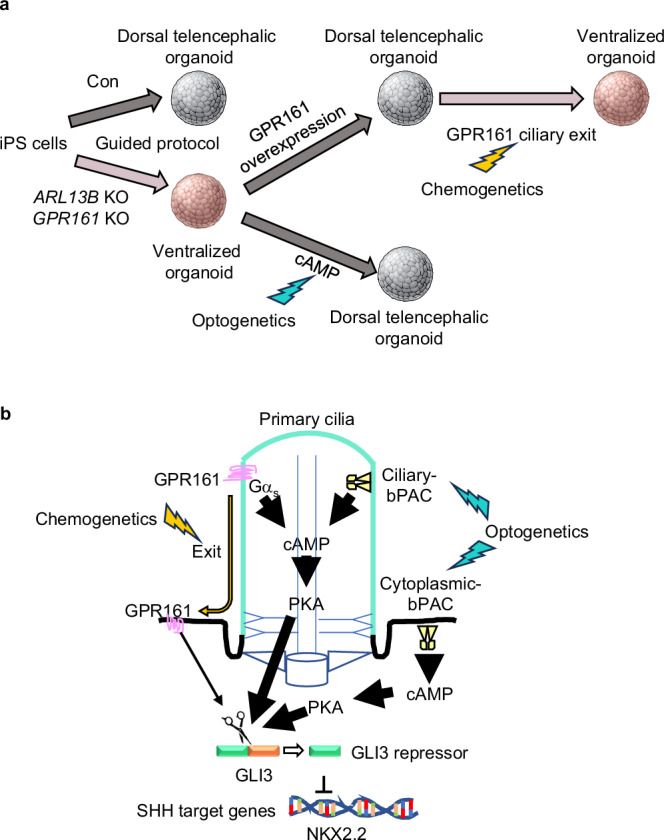
Manipulation of ciliary components regulates dorsal/ventral fate of neural stem/progenitor cells in human telencephalic organoids. **a** Overview of approaches used to manipulate ciliary components in telencephalic organoids using CRISPR/Cas9 KO, optogenetics, and chemogenetics. **b** Schematic of optogenetic and chemogenetic control of ciliary signaling. Illustration from NIAID NIH BioArt Source (bioart.niaid.nih.gov/bioart/399).

Previous studies have shown that primary cilia extend from NPCs in the mouse cortex and various brain organoid models^24,26–28^, but little is known about the components of primary cilia and whether regulating the components in cilia is sufficient for regulation of fate decisions of NPCs. Here, we generated telencephalic organoids using a commonly used protocol with dual SMAD inhibitors and a WNT inhibitor^33^. In the current study, we found various signaling components, such as GTPase proteins, in primary cilia of human telencephalic organoids using a proximity biotinylation assay (Fig. 1). We showed that *ARL13B* deficiency leads to ventral differentiation of NPCs in telencephalic organoids despite using the guided differentiation protocol (Fig. 3). Additionally, *GPR161* deletion induced ventralization of NPCs (Fig. 4). Mechanistically, we revealed that GPR161 regulates the SHH signaling pathway (Fig. 4). Remarkably, upregulation of cAMP by forskolin treatment and cAMP activation by optogenetics successfully restored dorsal identity of *GPR161* KO NPCs. This rescue by forskolin was accompanied by increased levels of GLI3 repressor formation; however, assessment of the role of the GLI3 repressor in the *GPR161* KO background requires careful analysis in future studies (Fig. 5). It is important to note that an increase in ciliary cAMP levels alone by optogenetics was sufficient to restore dorsal identity in *GPR161* KO organoids (Fig. 5). Lastly, using coordination chemogenetics, induction of GPR161 exit from primary cilia was sufficient to induce ventralization of NPCs, although the extent of ventralization was less than that caused by *GPR161* deletion (Fig. 6). Here, we show that (1) primary cilia of human NPCs are enriched in signaling cascade components revealed by proximity labeling followed by mass spectrometry, (2) ciliary-localized cAMP upregulation is sufficient to affect the fate decisions of NPCs in human telencephalic organoids, and (3) coordination chemogenetics can be applied to an orphan GPCR to regulate the fate decisions of human NPCs (Fig. 7). These findings suggest that ciliary signaling is sufficient to determine the dorsal/ventral fate of human NPCs.

SHH signaling determines dorsal/ventral fate of the neural tube in various animal models^15,55,56^. Upregulation of *GLI1* in both *ARL13B* and *GPR161* KO organoids suggests that SHH signaling contributes to ventralization. GLI1 acts as an amplifier of the SHH signaling pathway. A recent study showed that GLI1 also functions as a transcription factor after ciliary localization and subsequent nuclear translocation, where it binds SHH target genes^14^. SMO also accumulated in the cilia of *GPR161* KO organoids, indicating activation of SMO-mediated inhibition of PKA^57^. Indeed, GLI3 repressor formation was decreased in *GPR161* KO organoids. In agreement with our study, a previous study showed that *GLI3* deletion is critical for dorsal/ventral fate decisions of NPCs in unguided brain organoids^31^. These data indicate that reduced GLI3 repressor is a major downstream effect in *GPR161* KO organoids. However, the precise roles of full-length GLI3 and GLI3 repressor in dorsal/ventral fate decisions remain to be examined. Notably, we did not observe increased GLI2 levels in the cilia of *GPR161* KO organoids as compared to control telencephalic organoids, suggesting that GLI2 may not be critical in this context. Interestingly, Brooks et al. recently showed that Gli2 is dispensable for rostral neural tube development in *Gpr161* KO mice^44^. They reported that Gli3 is critical for rostral neural tube closure, and both Gli2 and Gli3 are critical for midline-caudal neural tube closure in *Gpr161* KO mice^44^. Since GLI1 was increased and GLI3 repressor formation was decreased in *GPR161* KO organoids, the GLI activator/repressor balance is shifted toward activator function in our organoid model. Of note, the *GPR161* deletion phenotype appears different from the phenotype induced by *ARL13B* deletion in our organoids. *ARL13B* deletion induced organoids with NKX2.1, NKX2.2, and GSX2, suggesting a ventral telencephalon identity with lateral/medial ganglionic eminence (LGE/MGE)-like features, although NKX2.2 is not a canonical marker of ventral telencephalon. NKX2.2 is a commonly used marker for the ventralization of neural tube and hypothalamus region, with limited expression in the ventral telencephalon^58–60^. On the other hand, *GPR161* deletion induced organoids with NKX2.1, NKX2.2, and FOXA2, but not GSX2, suggesting a ventral forebrain identity with hypothalamus-like features. The interpretation is broadly consistent with the tripartite hypothalamus model, in which the anterior tuberal hypothalamus occupies a ventral position relative to the telencephalon^19^. It is also important to note that organoids may exhibit gene expression patterns distinct from those of in vivo models; therefore, we may have observed ectopic expression of non-canonical genes in the organoids. Nevertheless, compared to control organoids, which express PAX6 but not NKX2.1, NKX2.2, GSX2, or FOXA2, deletion of *ARL13B* or *GPR161* induced ventralization-associated changes in NPC markers under a commonly used protocol for telencephalic organoid generation.

The importance of primary cilia in human brain development is underscored by heterogeneous human diseases called ciliopathies, including Joubert syndrome, Bardet-Biedl syndrome, neural tube defects, and intellectual disability^4,7^. Roles of primary cilia in NPCs are well studied in mice. However, it was difficult to examine ciliary signaling in human brain before the era of brain organoid research. Recent studies showed that brain organoids contain primary cilia in the ventricular zones^25,26^. In agreement with our study, other ciliary genes, such as *INPP5E* and *GLI3*, are involved in dorsal/ventral determination of NPCs in human telencephalic organoids using a guided differentiation protocol and brain organoids using a non-guided differentiation protocol^26,31^. Another study also revealed the importance of primary cilia in differentiation of NPCs into basal progenitor cells^61^. These data indicate that primary cilia are a critical organelle in differentiation of human NPCs and could be a druggable target in the future.

Although coordination chemogenetics is a useful method to inhibit GPR161, we were not able to generate iPS cells harboring endogenous *GPR161*_*HHC*_ mutations, which are required for coordination chemogenetics. Therefore, we expressed *CAG-driven GPR161*_*HHC*_ from the AAVS1 locus on chromosome 19 in *GPR161* KO iPS cells. The CAG promoter was chosen to insert the gene of interest into the AAVS1 locus, since other commonly used promoters can be silenced in iPS cells and differentiated cells^62,63^. Although insertion of a gene of interest into the AAVS1 locus is commonly used, it is possible that the endogenous *GPR161* promoter may yield distinct expression levels of *GPR161*, which may alter GPR161 function in regulating dorsal/ventral formation of telencephalic organoids. The mechanism underlying the decreased GFP intensity of GPR161_HHC_ in NPCs remains to be determined. The GPR161_HHC_ mutation may reduce ciliary localization by impairing trafficking to primary cilia or by reducing retention in primary cilia, resulting in decreased accumulation in the cilia. Nevertheless, we show that coordination chemogenetics can be applied to an orphan GPCR and a ciliary GPCR, and thereby expands our understanding of the role of GPCRs in brain development.

## Methods

### Human fetus immunostaining

This experiment was approved by the IRB of Nagoya City University (60-24-0081). Fetal human brain sections (15 gestational weeks; sex unknown) were previously collected at National Center of Neurology and Psychiatry in Japan with parental consent and without compensation. Paraffinized sections of human brain were rehydrated and autoclaved with Immunosaver (Nissin EM, Cat# 333). Sections were then immunostained. The slides were destroyed after analysis.

### iPS cell culture

Previously characterized human induced pluripotent stem cells (iPS cells; 201B7 (RIKEN; female) and #51 (also known as Windy; Dr. Akihiro Umezawa at National Center for Child Health and Development; male) were used in the current study^64–67^. iPS cells were maintained in Stemfit (Ajinomoto, RCAK02N) medium with iMatrix-511 (Takara Bio, T311). The medium was changed every one to three days for routine maintenance. iMatrix-511 was used in an uncoated manner (0.2 µg/cm^2^)^68^. For routine maintenance, iPS cells were passaged every five to seven days. For passage, cells were rinsed once with 0.5 mM EDTA, and then incubated with 0.5 mM EDTA/TrypLE Express Enzyme (Gibco, 12604021) (1:1 ratio) for 5 min at 37 °C. Cells were then collected in a tube and centrifuged at 190 × *g* for 3 min. Cell numbers were counted, and cells were seeded at a density of 750 cells/cm^2^ for passage. After passage, the culture medium was supplemented with 10 µM Y-27632 (Chemscene, CS-0878) for the first 24 h. 150 μL of Stem Cell Banker (Takara Bio, CB045) was routinely used for cryopreservation of 1–50 × 10^4^ cells. Cells were used for experiments between passages 40–60. Cells were tested for mycoplasma by PCR. Primers are listed in Supplementary Table 1.

### Generation of *ARL13B* and *GPR161* knockout iPS cells

Clustered regularly interspaced short palindromic repeats (CRISPR)/Cas9-mediated genome editing was used to delete *ARL13B* or *GPR161* in iPS cells. To prepare ribonucleoprotein (RNP) complexes, 2 µL of 100 µM CRISPR crRNA (Integrated DNA Technologies) were mixed with 2 µL of 100 µM Alt-R tracrRNA with Atto^TM^ 550 (Integrated DNA Technologies, 1075927), followed by incubation at 95 °C for 5 min and then at room temperature to form a single guide RNA (sgRNA) complex. One µL of 62 µM Alt-R S.p. HiFi Cas9 protein (Integrated DNA Technologies, 1081060) was added to the sgRNA complex and incubated in the dark at room temperature for 30 min to form an RNP complex. Subsequently, 0.5–1 × 10^6^ iPS cells were harvested and electroporated with 5 µL of the prepared RNP complex dissolved in 100 µL electroporation solution (6.6 mM ATP, 11.8 mM MgCl_2_-6H_2_O, 86.4 mM KH_2_PO_4_, 14.0 mM NaHCO_3_, 2.18 mM glucose, pH7.4) using Nucleofector 2b (Program #B-016; Lonza). Cells were centrifuged in DMEM/F12 medium (Invitrogen, 11330-032) and plated for cell culture. The next day, cells were harvested and stained with DAPI (Sigma-Aldrich, D8417) in Stemfit medium. DAPI-negative and Atto^TM^ 550-positive iPS cells were analyzed or isolated using a cell sorter (BD Biosciences, FACSCanto II, FACSAria II, FACSAria III) and plated in a dish at a cell density of 100–200 cells per 10 cm dish. The following day, cells were observed, and individual iPS cell colonies were clonally expanded. Non-clonal colonies were manually scratched with a 20-µL tip and removed from the plate under a microscope (Olympus, CKX53). Five to seven days after sorting, cells were treated with 0.5 mM EDTA for 3 min and 3 mL of DMEM/F12 medium were added to the plate. Single colonies were picked manually with a 20-µL tip and plated into a 24-well plate, and cultured for 7–14 days. After expansion of clones, cells were harvested. Half of the cells were stored for DNA extraction, while the other half was frozen for passage. Genomic DNA was extracted in Proteinase K lysis buffer (10 mM Tris-HCl pH8.8, 50 mM KCl, 2 mM MgCl_2_, 0.45% NP-40, 0.45% Tween 20, 0.2 mg/mL Proteinase K), and PCR was performed to examine the sequence of the target region to obtain knockout cell lines. Cell lines generated in this study are available from the corresponding authors upon request, after obtaining permission from RIKEN and the National Center for Child Health and Development. Primer sequences are listed in Supplementary Table 1.

### Plasmid construction

*pAAVS1-CAG-NPHP3*_*1–203*_*-EGFP-APEX2* plasmid was generated from *pEF5B-FRT-cilia-APEX* plasmid (Addgene 73186). Mutagenesis was used to generate *APEX2* from *APEX*, and then *NPHP3*_*1–203*_*-EGFP-APEX2* was inserted into the *pAAVS1-CAG-EGFP* plasmid (Addgene 80491^69^). *pAAVS1-CAG-EGFP-APEX2* plasmid was generated from *pAAVS1-CAG-NPHP3*_*1–203*_*-EGFP-APEX2* plasmid*. pAAVS1-CAG-bPAC-EGFP* plasmid was generated from *pgLAP5-CRYS-Arl13b-bPAC-EGFP* plasmid (gift from Dr. Jeremy Reiter at UCSF^2^), *pAAVS1-P-CAG-mCh* plasmid (Addgene 80492^69^), and *pAAVS1-CAG-EGFP* plasmid using In-Fusion HD Cloning Kit (Takara, 639650). *bPAC* was inserted into *pAAVS1-CAG-EGFP* plasmid. To generate *pAAVS1-CAG-bPAC*_*F198Y*_*-EGFP* plasmid, F198Y mutation was inserted using mutagenesis kit (TOYOBO, KOD-Plus-Mutagenesis kit, Cat# SMK-101).　*pAAVS1-CAG-ARL13B-bPAC-EGFP* plasmid was generated from *pgLAP5-CRYS-Arl13b-bPAC-EGFP* plasmid, *pAAVS1-P-CAG-mCh* plasmid and *pAAVS1-CAG-EGFP* using In-Fusion HD Cloning Kit. *ARL13B-bPAC* was inserted into *pAAVS1-CAG-EGFP* plasmid. To generate *pAAVS1-CAG-ARL13B-bPAC*_*F198Y*_*-EGFP* plasmid, F198Y mutation was inserted using a mutagenesis kit. The cDNA region of human *GPR161* (CCDS1268.1) was amplified from human brain Marathon-Ready cDNA library (Clontech, 639300). The coding sequence was subcloned into NheI/NotI restriction site of *pCDMd1* vector, which contains the CMVd1 promoter from the PFN22A HaloTag CMVd1 Flexi vector (Promega, G2841). Site-directed mutagenesis was performed to generate *GPR161*_*HHC*_ using the Q5 Site-Directed Mutagenesis Kit (NEB, E0554) or NEBuilder HiFi DNA Assembly Master Mix (NEB, E2621). The expression vector of *Gα15* was constructed by subcloning of the coding sequence of *Gα15* into pCAGGS vector (kindly gifted from Dr. Jun-ichi Miyazaki)^70^. *pAAVS1-CAG-GPR161-EGFP* plasmid and *pAAVS1-CAG-GPR161*_*HHC*_*-EGFP* plasmid were generated from *pCDMd1-GPR161* plasmid, *pCDMd1-GPR161*_*HHC*_ plasmid and *pAAVS1-CAG-EGFP* plasmid. *pCDMd1-GPR161-EGFP* plasmid and *pCDMd1-GPR161*_*HHC*_*-EGFP* plasmid were generated from *pAAVS1-CAG-GPR161-EGFP*, *pAAVS1-CAG-GPR161*_*HHC*_*-EGFP* and *pCDMd1* plasmids.

### CRISPR/Cas9 knock-in at the AAVS1 locus

To generate iPS cells expressing a gene of interest, we utilized a published protocol to insert one copy of the gene of interest into the *AAVS1* locus on chromosome 19^69^. 201B7 iPS cells and 201B7 *GPR161* KO2 iPS cells were plated into 24-well plates at a density of 6000 cells/well. The next day, cells were transfected with pXAT2 (Addgene #80494^69^), and *pAAVS1-CAG-NPHP3*_*1–203*_*-EGFP-APEX2* construct for *cilia-EGFP-APEX2* iPS cells*, pAAVS1-CAG-bPAC-EGFP* construct for cyto*-bPAC-EGFP GPR161* KO iPS cells, *pAAVS1-CAG-Arl13B*-*bPAC-EGFP* construct for *cilia-bPAC-EGFP GPR161* KO iPS cells, *pAAVS1-CAG*-*bPAC*_*F198Y*_*-EGFP* construct for *cyto-bPAC*_*F198Y*_*-EGFP GPR161* KO iPS cells, *pAAVS1-CAG-Arl13B-bPAC*_*F198Y*_*-EGFP* construct for *cilia-bPAC*_*F198Y*_*-EGFP GPR161* KO iPS cells, *pAAVS1-CAG-GPR161-EGFP* construct for *GPR161-EGFP GPR161* KO iPS cells and *pAAVS1-CAG-GPR161*_*HHC*_*-EGFP* construct for *GPR161*_*HHC*_*-EGFP GPR161* KO iPS cells using Lipofectamine Stem Transfection Reagent (Thermo Fisher Scientific, STEM00001). Cells were cultured to confluency and GFP-positive cells were sorted using FACS (FACSAria II, FACSAria III). Cells were plated onto 60-mm dishes and cultured to confluency. Then GFP-positive cells were sorted using FACS for the second time. Cells were plated onto 60-mm dishes and then used for experiments or cryopreservation. We also tried to use Crys (very weak) or CMVd1 (weak) promoters instead of the CAG promoter for insertion into the AAVS1 locus; however, no EGFP-positive iPS cells were obtained. Therefore, we did not use the Crys or CMVd1 promoters.

### Generation of telencephalic organoids from iPS cells

To generate telencephalic organoids, we used a dual SMAD inhibitor protocol with slight modifications^33^. We seeded 3000 iPS cells into ultra-low attachment-coated 96-well U-bottom plates (Corning 7007; Thermo Fisher Scientific, 174925) and cultured them in induction medium (DMEM/F12 medium containing 20% knockout serum replacement (Thermo Fisher Scientific, 10828010), 1 mM MEM non-essential amino acids (Gibco, 11140050), 2 mM L-glutamine (Nacalai, 16948-04), 100 µM 2-mercaptoethanol (FUJIFILM Wako Pure Chemical, 133-14571), 100 nM LDN-193189 (Cayman Chemical, 19396), 10 µM SB-431542 (Cayman Chemical, 13031), and 2 µM XAV939 (Cayman Chemical, 13596)) with 50 µM Y-27632 (for the first 24 h) for the first 6 days with replacement every 2–3 days. To obtain a large number of organoids for protein/RNA collection, 188,000 iPS cells were seeded into EZ-sphere 24-well plates (approximately 400 cells per microwell; Iwaki, 4820-900SP). On day 6, the induction medium was removed and 2% Matrigel (Corning, 354234) was added to the bottom of the plate and cultured in organoid culture medium 1 (50% DMEM/F12 medium, 50% MACS Neuro Medium (Miltenyi Biotec, 130-093-570), 1 mM MEM non-essential amino acids, 2 mM L-glutamine, 50 µM 2-mercaptoethanol, 400 nM insulin (Nacalai, 12878-86), 0.5 × N-2 MAX Media Supplement (R&D Systems, AR009), 100 U/ml penicillin/streptomycin (Nacalai, 26253-84) and 0.5 × MACS NeuroBrew21 supplement without vitamin A (Miltenyi Biotec, 130-097-263)) for 8 days. The medium was replaced every 2–3 days. Organoids were transferred to 6-well plates on day 14. Six-well plates can be pre-coated with 2-hydroxyethyl methacrylate solution (1.2 g of 2-hydroxyethyl methacrylate (Sigma-Aldrich, P3932) in 40 mL of 95% ethanol) for low binding conditions (optional). To mature the organoids, they were cultured in organoid culture medium 2 (50% DMEM/F12 medium, 50% MACS Neuro Medium, 1 mM MEM non-essential amino acids, 2 mM L-glutamine, 50 µM 2-mercaptoethanol, 400 nM insulin, 0.5 × N-2 MAX Supplement, 100 U/mL penicillin/streptomycin, 0.5 × MACS NeuroBrew21 with vitamin A (Miltenyi Biotec, 130-093-566), and 200 µM L-ascorbic acid (Sigma-Aldrich, A92902)) from day 14 up to one month. Plates were rotated on an orbital shaker (TAITEC, CS-LR) at 70 rpm. The culture medium was replaced every 1–4 days.

### 2D neural stem/progenitor cells (NPCs) culture

iPS cell-derived NPCs were generated^67,71,72^. 188,000 iPS cells were seeded into EZ-sphere 24-well plates and cultured in induction medium with 50 µM Y-27632 (for the first 24 h) for the first 6 days with replacement every 2–3 days (approximately 400 cells/microwell). On the sixth day, spheres were seeded into 6-well plates that had been coated with 20 ng/mL laminin (Corning, 354232) in poly-D-lysine (R&D Systems, 3439-100-01) for 1 h at 37 °C. Attached spheres were cultured in Neural progenitor cell medium (50% DMEM/F12 medium and 50% MACS Neuro Medium containing 1 mM MEM non-essential amino acids, 2 mM L-glutamine, 0.5 × N-2 MAX media supplement, 0.5 × MACS NeuroBrew21, 20 ng/mL FGF-2 (gift from Dr. Spees), 20 ng/mL EGF (Alomone Labs, E-100), 100 U/mL penicillin, and 100 µg/mL streptomycin). Sphere-derived monolayer cells were cultured until neural rosette formation, with replacement of the medium every 2–3 days. Subsequently, cells were dissociated, and were passaged into a new Poly-D-Lysine/laminin-coated dish with Y-27632, and cryopreserved in Stem Cell Banker.

### APEX2 proximity labeling for mass spectrometry analysis

Telencephalic organoids were generated from *cyto-EGFP-APEX2* iPS cells and *cilia-EGFP-APEX2* iPS cells and analyzed at 2 weeks. This time point was chosen because of an enriched neural progenitor cell population in telencephalic organoids, compared to 1-month-old telencephalic organoids. Matrigel was omitted to avoid Matrigel-derived proteins in our analysis. Telencephalic organoids were chopped and treated with 0.5 mM biotin-tyramide (Biotin-phenol; Focus Biomolecules, 10-3446) at 37 °C for 45 min, and then treated with 30% H_2_O_2_ (FUJIFILM Wako Pure Chemical, 081-04215) for 1 min. Three types of telencephalic organoids (*cyto-APEX2* telencephalic organoids treated with H_2_O_2_ (*n* = 3 independent experiments), *cilia-APEX2* telencephalic organoids treated with H_2_O_2_ (*n* = 6 independent experiments) and *cilia-APEX2* telencephalic organoids not treated with H_2_O_2_ (*n* = 6 independent experiments)) were washed three times with quenching buffer (5 mM Trolox (Cayman, 10011659), 10 mM sodium ascorbate (Nacalai, 03422-32) and 0.1% sodium azide (FUJIFILM Wako Pure Chemical, 194-01275) in DMEM/F12 medium). RIPA lysis buffer (150 mM NaCl (Sigma-Aldrich, S3014), 1% NP-40, 0.5% sodium deoxycholate (Sigma-Aldrich, D6750), 0.1% SDS (Sigma-Aldrich, 28-3260-5), 50 mM Tris-HCl pH7.4) containing 1% protease and phosphatase inhibitor cocktail (Thermo Fisher Scientific, 78445), 5 mM Trolox, 10 mM sodium ascorbate and 0.1% sodium azide was added and sonication was performed on telencephalic organoids. Samples were centrifuged to obtain supernatant proteins. Streptavidin magnetic beads (Pierce, 88816) were added and samples were rotated overnight at 4 °C.

Samples were washed with RIPA buffer, 1 M KCl (Nacalai, 28514-75), 0.1 M NaHCO_3_ (Sigma-Aldrich, 28-1850-5), and 1 M Urea (Nacalai, 35940-81) in 10 mM Tris-HCl (pH 8.0). Samples were reduced and alkylated with 25 mM TCEP (Thermo Fisher Scientific, 77720) and 25 mM 2-chloroacetamide (FUJIFILM Wako Pure Chemical, 032-09762) in on-bead digestion buffer (50 mM pH8.0 HEPES (Sigma-Aldrich, H4034), 1 µM CaCl_2_ (FUJIFILM Wako Pure Chemical, 039-00475), 2% acetonitrile (Kanto Chemical, 01033-23), ultrapure water (FUJIFILM Wako Pure Chemical, 214-01301) at 55 °C for 30 min. Samples were washed with on-bead digestion buffer, and incubated with 1 µg trypsin (Pierce, 90057) in on-bead digestion buffer at 37 °C for 16 h. A Dynal MPC-S magnetic particle concentrator (Invitrogen, 120.20D) was used to separate beads and supernatant was transferred to a new tube. Samples were adjusted to 1% trifluoroacetic acid (FUJIFILM Wako Pure Chemical, 206-10731) and desalted by SDB-RPS (CDS, 2241) stage tip. Dried peptides were reconstituted in 1% formic acid/2% acetonitrile and 200 ng of peptides were analyzed by liquid chromatography-tandem mass spectrometry (LC-MS/MS).

### RNA-seq analysis

Total RNA was isolated from telencephalic organoids at two weeks. This time point was chosen to reduce the variability of cells in telencephalic organoids. mRNA was purified from total RNA using poly-T oligo-attached magnetic beads. mRNA libraries were prepared using the NEBNext Ultra II RNA library prep kit for Illumina (NEB, E7770) and were sequenced using a Novaseq X Plus sequencer (150 bp, paired-end) by Nippon Genetics. Raw FASTQ files were trimmed with FASTP (v0.23.2) and mapped with HISAT2 (v2.2.1). Read counts and TPM were calculated with featureCounts (SUBREAD, v2.0.1) and Rnanorm (v2.1.0). Except for genes with a read count of less than 10 and mitochondria genes, log fold change and *p*-values were calculated using pydeseq2 (Supplementary Data 2)^73^. To generate the heatmap, we used the Transcripts Per Million (TPM) of each sample. GraphPad Prism was used for the analysis. Data were also analyzed with BioJupies (https://maayanlab.cloud/biojupies/)^74^. KEGG pathway enrichment analysis was performed using BioJupies^75^. The *p*-values were calculated using Fisher’s exact test, and adjusted for multiple comparison using the Benjamini–Hochberg method. Bold pathway names indicate false discovery rate (FDR) < 0.1. For the volcano plot, differential expression analysis was performed using BioJupies^76^. Statistical significance was assessed using two-sided moderated *t*-tests, and *p*-values were adjusted for multiple comparisons using the Benjamini–Hochberg method.

### Optogenetics

We generated a custom LED device to automatically control 470-nm LED exposure. The device consisted of Arduino Uno SMD R3 (Arduino, A000073), Gravity-relay module (DFRobot, DFR0643), and LED device (Optocode, LEDB-SBOXHP). We performed optogenetic experiments in 96-well low-binding U-bottom plates and 6-well plates using the custom LED device. During handling of cells, the cell culture room was kept dark using a 660-nm LED stand light (Optocode, LED660-100STND). The 470-nm LED was controlled by an Arduino Uno microcontroller, which was programmed with a custom script written in the Arduino Integrated Development Environment (Version 2.3.1; Supplementary Fig. 10c, d). We stimulated organoids with 470-nm blue light for 2 weeks using a pulsed pattern (100 ms ON, 2 min OFF or 500 ms ON, 2 min OFF) (Supplementary Fig. 10e, f).

### Putative GPCR structures and overlapping images

Analysis of protein structure was performed^54^. We obtained structures of GPCRs (A_2A_R (Protein Data Bank ID (PDB ID): 2YDO) and GPR161 (PDB ID: 8SMV)) from the RCSB Protein Data Bank (http://www.rcsb.org). Structures were processed in PyMOL (v3.1.4.1) to remove water molecules, to highlight GPCRs in colors, and to substitute target residues with Cys or His. PyMOL was also used to overlap structures between two GPCRs and to calculate the root mean square deviation between the GPCRs.

### Coordination chemogenetics

Telencephalic organoids were generated using *GPR161-EGFP GPR161* KO iPS cells and *GPR161*_*HHC*_*-EGFP GPR161* KO iPS cells. Telencephalic organoids were treated with 10 µM copper(II) chloride starting on day 6 and fixed on day 14 for further analysis.

### LC-MS/MS analysis

Peptides were analyzed with Vanquish Neo UHPLC system equipped with a C_18_ column (Nikkyo Technos, NTCC-360/75-3-125), FAIMS Pro Duo interface, and an Orbitrap Exploris 240 mass spectrometer. Peptides were fractionated with a gradient of solvent A (0.1% formic acid in water) and solvent B (80% acetonitrile, 0.1% formic acid) from 6 to 31% solvent B over 90 min, then from 31 to 50% over 5 min at a flow rate of 250 nL/min. Compensation voltage was set to −45 V with 1.2 L/min of total carrier gas flow in FAIMS. MS and MS2 spectra were obtained in full MS/DIA mode. The full scan range was set to 480–800 *m/z* at 60,000 resolution and DIA scan windows were set to staggered 8 *m/z* at 30,000 resolution. Details regarding LC and MS parameters are provided in the deposited raw files.

### Mass spectrometry-based label-free protein quantification

Proteomic raw data were analyzed with DIA-NN 2.0.2 Academia. An in silico spectral library was generated using UniProt human FASTA (UP000005640, downloaded in October 2024) and common contaminant^77^ with the additional variable modifications of methionine oxidation and N-terminal acetylation. The precursor charge and *m/z* ranges were set to 2–4 and 480–800, respectively. Mass accuracy, MS1 accuracy, and scan window in algorithm section were all set to 11. Optional filtering with –matrix-spec-q was also set. Other parameters were set to default values.

Differentially expressed proteins in *cilia-APEX2* group were identified using the following criteria: (1) non-detected proteins in the negative control group were assigned a value of 1 for calculation purposes, (2) a fold change > 1.5 relative to the negative control and *cyto-APEX2* group, (3) detection of the protein in at least two H_2_O_2_-treated *cilia-APEX2* group. Systematic GO term analysis was performed using g:Profiler (https://biit.cs.ut.ee/gprofiler/gost). GO analysis was also performed using the following GO terms; GO: 0030695 GTPase Regulator Activity, GO: 0005813 Centrosome, GO: 0007224 Smoothened signaling pathway, GO: 0016055 Wnt Signaling Pathway, GO: 0016310 Phosphorylation, GO: 0000151 Ubiquitin Ligase Complex, GO: 0005929 Cilium, GO: 0019933 cAMP-Mediated Signaling, GO: 0007219 Notch Signaling Pathway, GO: 0019221 Cytokine-Mediated Signaling Pathway.

### Sample processing, antibodies, immunostaining, and microscopy

Telencephalic organoids were fixed with 4% paraformaldehyde (FUJIFILM Wako Pure Chemical, 162-16065) in PBS for 3–4 h at 4 °C, and incubated in 30% sucrose (Nacalai Tesque, 09589-05) in PBS for 1 day at 4 °C. The length of paraformaldehyde fixation is important for GPR161 immunohistochemistry. Telencephalic organoids were mounted with OCT compound (Sakura Finetek Japan, 4583) and cut into 16 µm frozen sections. For immunostaining using frozen sections, sections were incubated in PBS for 15 min to dissolve the OCT. Sections were then blocked in blocking buffer (3% normal serum block (BioLegend, 927503), and 0.4% Triton X-100 (Sigma-Aldrich, 30-5140-5) in PBS) for 1 h at room temperature. For OLIG2 staining, FBS blocking buffer was used (3% fetal bovine serum (FBS; Sigma-Aldrich, 172012), 0.4% Triton X-100 in PBS). Sections were incubated with primary antibodies (dilution, 1:500 unless otherwise stated) against the following antigens overnight at room temperature: acetylated α-tubulin (Sigma-Aldrich, T6793), ARL13B (Proteintech, 17711-1-AP), ARL13B (BioLegend, 857602), β-catenin (Sigma-Aldrich, C2206), β-tubulin III (Santa Cruz, sc-80005), BLBP (Millipore, ABN14), FOXA2 (HNF-3b; Santa Cruz, sc-101060), FOXG1 (Abcam, ab18259), γ-tubulin (Santa Cruz, sc-17787), γ-tubulin (Santa Cruz, sc-51715), GFP (Santa Cruz, sc-51715), GLI2 (R&D Systems, AF3526), GPR161 (Proteintech, 13398-1-AP), GPR161 (Sigma-Aldrich, AV42354), GSX2 (Sigma-Aldrich, ABN162), NKX2.1 (Diagnostic Biosystems, MOB285-01), NKX2.2 (1:20, Hybridoma Bank, 74.5A5), OCT3/4 (Santa Cruz, sc-5279), OLIG2 (R&D Systems, AF2418), PAX6 (BioLegend, 901301), PAX7 (1:20, Hybridoma Bank, Pax7), pH3 (1:1000, Santa Cruz, sc-374669), Poly-E (1:1000, GT335; Adipogen, AG-20B-0020), SMO (Santa Cruz, sc-166685) and SOX2 (Santa Cruz, sc-365823). After 3 PBS washes, sections were incubated with secondary antibodies (Alexa Fluor 488-, 555-, 594-, 647-conjugated secondary antibodies; Invitrogen; dilution, 1:500 unless otherwise stated) for 1 h at room temperature. To visualize APEX2 signaling, we treated sections with iFluor555-streptavidin conjugate (1:1000, AAT Bioquest, Cat# 16959) for 1 min at room temperature. Cell nuclei were stained with DAPI. Slides were mounted with Fluoromount-G (SouthernBiotech, 0100-01) or CC/Mount (Diagnostic Biosystems, K002). Images were acquired with the following microscopes (10×, 20×, 40×, 63×, 100× lenses; Olympus FV3000, Olympus SpinSR, Nikon AX R, Nikon AX R MP with NSPARC or Nikon A1).

### Immunocytochemistry of iPS cells

iPS cells were plated on a plastic coverslip (Sumitomo Bakelite, MS-92132Z) in a 24-well plate. To examine primary cilia, cells were starved for 24 h in DMEM/F12 medium. Cells were fixed with 4% paraformaldehyde for 15 min at room temperature. Cells were blocked in blocking buffer and stained with antibodies. The plastic cover glass was mounted on a slide and images were acquired with microscopes.

### Immunocytochemistry of NPCs

NPCs were plated on a Poly-D-lysine/laminin coated-cover glass (Marienfeld Superior™ Borosilicate Glass Cover Glasses, Round, Paul Marienfeld GmbH, 0111520) in a 24-well plate. Cells were fixed with ice-cold methanol (Sigma-Aldrich, 19-2410-4) for 15 min at 4 °C. Cells were blocked in blocking buffer and stained for antibodies. The cover glass was mounted on a slide and images were acquired with microscopes.

### Cell growth curve

iPS cells were seeded in 24-well plates at a density of 2500 cells/cm^2^. To quantify the number of cells, cells were treated with 200 µL of TrypLE/0.5 mM EDTA solution and pipetted. Ten µL of solution was taken and the number of cells was quantified on a hematocytometer.

### RNA isolation and qRT-PCR

Total RNA was isolated from 10 to 30 telencephalic organoids using Isogen II (NipponGene, 311-07361) or an RNA premium kit (Nippon Genetics, FG-81050), according to the manufacturers’ instructions. cDNA was generated using ReverTra Ace qPCR RT Master Mix with gDNA remover (Toyobo, FSQ-301), according to the manufacturer’s instructions. qRT-PCR was performed with FastStart universal SYBR green master (Sigma-Aldrich, 4913914001). Reactions were run using a real-time PCR machine (ThermoFisher, QuantStudio 12 K Flex). Primer sequences are listed in the Supplementary Table 1.

### CUT&Tag data of GLI3 binding

CUT&Tag data of GLI3 binding in 23-day-old cerebral organoids from human iPS cell line 409B2 was re-analyzed from published data^31^. Raw FASTQ files (downloaded from E-MTAB-12006) were processed through fastp (v0.23.2)^78^ and mapped with Bowtie2 (v2.2.5)^79^ to the human genome hg38. The resulting SAM file was converted to bigWig file using SAMtools (v1.15.1)^80^ and deepTools (v3.5.1)^81^. Peak calling was performed using HOMER (v4.11)^82^. Signal tracks of GLI3 binding were visualized using IGV (v2.17.4)^83^.

### Western blot

To obtain protein extracts for Western blot analysis, 10–30 telencephalic organoids were rinsed twice with ice-cold PBS and frozen until use. Frozen samples were lysed with RIPA lysis buffer containing 1% protease and phosphatase inhibitor cocktail for 3–4 h at 4 °C. The lysate was centrifuged (15,000 × *g*, 4 °C) for 30 min, and the supernatant was used for further analysis. 4× SDS sample buffer (0.225 M Tris-HCl pH6.8, 50% glycerol (Sigma-Aldrich, 12-1120-3), 5% SDS, 0.05% bromophenol blue (Sigma-Aldrich, 03-4140-3), 0.25 M DTT (Nacalai Tesque, 14128-62)) was added, and samples were boiled at 100 °C for 5 min. Samples were subjected to 9% homemade SDS-polyacrylamide gel or Bullet 5–20% PAGE Plus precast gel (Nacalai Tesque, 21793-64) electrophoresis with running buffer (25 mM Tris, 192 mM glycine, 0.1% SDS), followed by transfer to polyvinylidene difluoride membranes (Millipore, IPVH00010) with transfer buffer (50 mM Tris, 192 mM glycine, 20% methanol). Non-specific binding was blocked with 5% skim milk in 0.1% Triton X-100 in PBS (PBST) for 1 h at room temperature or Bullet blocking one for western blotting (Nacalai Tesque, 13779-01) for 5 min, and proteins were probed with primary antibodies {ARL13B (Proteintech, 17711-1-AP), β-actin (Santa Cruz, sc-69879), GAPDH (Novus, NB300-322), GLI3 (R&D Systems, AF3690)}, diluted 1:2500 in 0.1% PBST at 4 °C overnight or Bullet ImmunoReaction buffer (Nacalai Tesque, 18439-85) at room temperature for 30 min. After washing with 0.1% PBST, horseradish peroxidase (HRP)-labeled secondary antibodies against rabbit, mouse, or goat IgG were probed for 1 h at room temperature at a dilution of 1:2500 in 0.1% PBST or 30 min at room temperature in Bullet ImmunoReaction Buffer. Luminata Forte Western HRP Substrate (Millipore, WBLUF0500) or SuperSignal West Atto Ultimate Sensitivity Chemiluminescent Substrate (ThermoFisher Scientific, A38556) was used to detect the signal. All images were acquired with an Amersham Imager 680 (GE Healthcare). Subsequently, the blot was treated with Western Blot Stripping Buffer (ThermoFisher Scientific, 21059) for 10–15 min at room temperature. The same blot was re-probed with anti-β-actin antibody or GAPDH antibody as a loading control. Primary and secondary antibodies are listed in Supplementary Table 1. Uncropped and unprocessed images are provided in the source data.

### Luciferase reporter assay for GPR161 signaling

HEK293 cells (ATCC, CRL-1573) were maintained in Dulbecco’s modified Eagle’s medium (DMEM) (Nacalai Tesque, 08458-16) supplemented with 10% FBS (Nichirei Biosciences, 175012-500 ML), 100 units mL^–1^ penicillin, and 100 µg mL^–1^ streptomycin (FUJIFILM Wako Pure Chemical, 168-23191) at 37 °C in a humidified atmosphere of 95% air and 5% CO_2_. HEK293 cells were transfected with plasmids (*pCDMd1-GPR161*, *pCDMd1-GPR161*_*HHC*_, *pCDMd1-GPR161-EGFP*, *pCDMd1-GPR161*_*HHC*_*-EGFP*, *pCDMd1* vector control, and *pCAGGS-Gα15*) using ViaFect (Promega, E4981) or Lipofectamine 3000 (Thermo Fisher Scientific, L3000001) according to the manufacturer’s instructions. After transfection, cells were cultured in DMEM with 10% FBS, 100 units mL^–1^ penicillin, and 100 µg mL^–1^ streptomycin at 37 °C in a humidified atmosphere of 95% air and 5% CO_2_ for 20 h. Cells were treated with copper(II) chloride (FUJIFILM Wako Pure Chemical, 039-04152) for 4 h. As a positive control, 10 µM forskolin (FSK) (Tokyo Chemical Industry, F0855) was used. HEK293 cells were utilized for Bright-Glo Luciferase assay (Promega, E2620) according to the manufacturer’s instructions. The signal was quantified with a FlexStation3 (Molecular Devices, FLEX3). Raw data from the FSK-treated group were set to 1 for calculation of relative luminescence units (RLU).

### Transmission electron microscopy (TEM)

Telencephalic organoids were fixed in 2% glutaraldehyde (Electron Microscopy Sciences, 16220-P), in 0.1 M phosphate buffer (PB; pH 7.4) at 4 °C overnight, and post-fixed in 2% osmium tetroxide (MERCK, 24505) in 0.1 M PB (pH 7.4) at 4 °C for 45 min. Samples were then dehydrated with a graded ethanol series and embedded in Quetol 812 epoxy resin (Nissin EM, 340) at 60 °C for 48 h. Ultrathin sections 80–100 nm thick were obtained with an Ultracut-UCT (Leica), stained with 2% uranyl acetate (Merck, 8473) solution for 15 min, and stained with modified Sato’s lead solution^84^ for 5 min. TEM observations were performed using an electron microscope (JEM-1400 Plus; JEOL).

### Scanning electron microscopy (SEM)

Samples were cut into two or more pieces using a 76 razor (Nissin EM, 4761) under a microscope, and then washed in organoid culture medium 2 in a 6-well plate for 30 min to remove debris from inside the ventricles. Samples were pre-fixed in a fixative containing 2% glutaraldehyde in 0.1 M PB (pH 7.4) for more than 24 h. After fixation, samples were post-fixed with 2% osmium tetroxide in 0.1 M PB for 1 h. These specimens were gradually dehydrated in ethanol, followed by critical point drying (Leica, CPD300). Samples were mounted on SEM specimen stubs, coated with a layer of sublimated OsO_4_ using an osmium plasma coater (OPC80N; Filgen, Nagoya, Japan). Images were acquired using a Field-Emission SEM (S-4800; Hitachi, Tokyo, Japan) at 5 kV.

### Declaration of generative AI in scientific writing

We have used ChatGPT, DeepL, and Grammarly to correct English grammar of the current manuscript. After correcting the grammar, the authors reviewed and edited the content as needed and take full responsibility for the content of the published article.

### Statistics and reproducibility

To quantify the number of cells, we took pictures of regions of interest and analyzed them using Fiji. Data from 1 to 20 ventricular zones in 3–22 telencephalic organoids in 1–5 independent experiments were used for the analysis, and the number of organoids is shown as n in graphs. Organoids were analyzed at 4–7 weeks of culture unless otherwise noted. To quantify lengths of cilia of NPCs in 2D culture, we took pictures of regions of interest using a 40 × lens with 2 × zoom (FV3000, Olympus) and quantified the length using Fiji manually. To quantify GFP intensity in primary cilia of telencephalic organoids, we took pictures of regions of interest with z-stack imaging using a 100 × lens (SpinSR10, Olympus) and quantified the intensity using Fiji manually. Organoids that failed to grow were excluded from the experiments and analysis. The experiments were not randomized. The investigators were not blinded to allocation during experiments and outcome assessment. Sample sizes were based on our experience with these assays. No statistical method was used to predetermine sample size. Representative data, including micrographs and Western blots, were confirmed in at least three independent experiments with consistent and reproducible outcomes, except for Fig. 1m–o (*n *= 1 biological sample). All data in figures are expressed as means ± standard deviation or means ± standard error of the mean (Fig. 6d and Supplementary Fig. 13a). To assess the statistical significance of differences among treatments, we performed unpaired, two-sided Student’s *t*-tests that assumed unequal variances between groups, one-way ANOVA with Sidak’s tests, one-way ANOVA with Tukey multiple comparisons tests, and Two-way ANOVA and Dunnett’s multiple comparisons test. GraphPad Prism (GraphPad, La Jolla, CA) was used for statistical analysis. Values of *p* < 0.05 were considered significant. In organoid experiments for quantifying specific area or cell number, *n* represents the number of organoids. For each independent experiment, samples were newly generated from distinct passages of each iPS cell.

### Reporting summary

Further information on research design is available in the Nature Portfolio Reporting Summary linked to this article.

## Supplementary information

Supplementary Information

Description of Additional Supplementary Files

Supplementary Data 1

Supplementary Data 2

Reporting Summary

Transparent Peer Review file

## Source data

Source Data

## Data Availability

RNA sequencing data have been deposited in the DNA Data Bank of Japan (DDBJ) BioProject (PRJDB20782 [https://ddbj.nig.ac.jp/search/entry/bioproject/PRJDB20782]). Mass spectrometry analysis data have been deposited in the Japan Proteome Standard Repository (JPST003807 [https://repository.jpostdb.org/entry/JPST003807])^85^. CUT&Tag data ERR10092480 was used for the reanalysis^31^. Requests for further information and resources should be directed to and will be fulfilled by the lead contact, Dr. Issei S. Shimada (ishimada@med.nagoya-cu.ac.jp). All unique reagents generated in this study are available from the lead contact with a completed materials transfer agreement. Source data are provided with this paper.

## Code availability

Code for the Arduino Uno gravity-relay module for optogenetics is available at 10.5281/zenodo.19492078.
