# Supplementary material for: Light- and chemical-induced ciliary signaling governs dorsal/ventral regionalization of human telencephalic organoids
Source: Nat Commun. 2026 May 22;17:6712. doi: 10.1038/s41467-026-73505-2 (PMC13385379; doi:10.1038/s41467-026-73505-2)
Supplement: Supplementary file 1 — Supplementary Information [file 41467_2026_73505_MOESM1_ESM.pdf]

Supplementary Information

Light- and chemical-induced ciliary signaling governs dorsal/ventral regionalization of human telencephalic organoids

Issei S. Shimada[1*], Akari Goto[1], Yutaka Hashimoto[1], Hajime Inoue[2], Takuto Sugawara[2], Tomohiro Doura[2], Tsubasa Fujita[1], Toshiaki Iwata[1], Riko Shimmoto[1], Hiroshi Takase[3], Masayuki Itoh[4], Shigeki Kiyonaka[2,5], Yoichi Kato[1*]

Author affiliations

1. Department of Cell Biology, Graduate School of Medical Sciences, Nagoya City University, Nagoya, Aichi, Japan, 467-8601
2. Department of Biomolecular Engineering, Graduate School of Engineering, Nagoya University, Nagoya, Aichi, Japan, 464-8603
3. Core Laboratory, Graduate School of Medical Sciences, Nagoya City University, Nagoya, Aichi, Japan, 467-8601
4. Department of Biochemistry and Cellular Biology, National Center of Neurology and Psychiatry, Kodaira, Tokyo, Japan, 187-8502
5. Research Institute for Quantum and Chemical Innovation, Institutes of Innovation for Future Society, Nagoya University, Nagoya, Aichi, Japan, 464-8603

* These authors contributed equally to the work: Issei S. Shimada and Yoichi Kato.
E-mail addresses: ishimada@med.nagoya-cu.ac.jp (I.S. Shimada),
kato.41@med.nagoya-cu.ac.jp (Y. Kato)

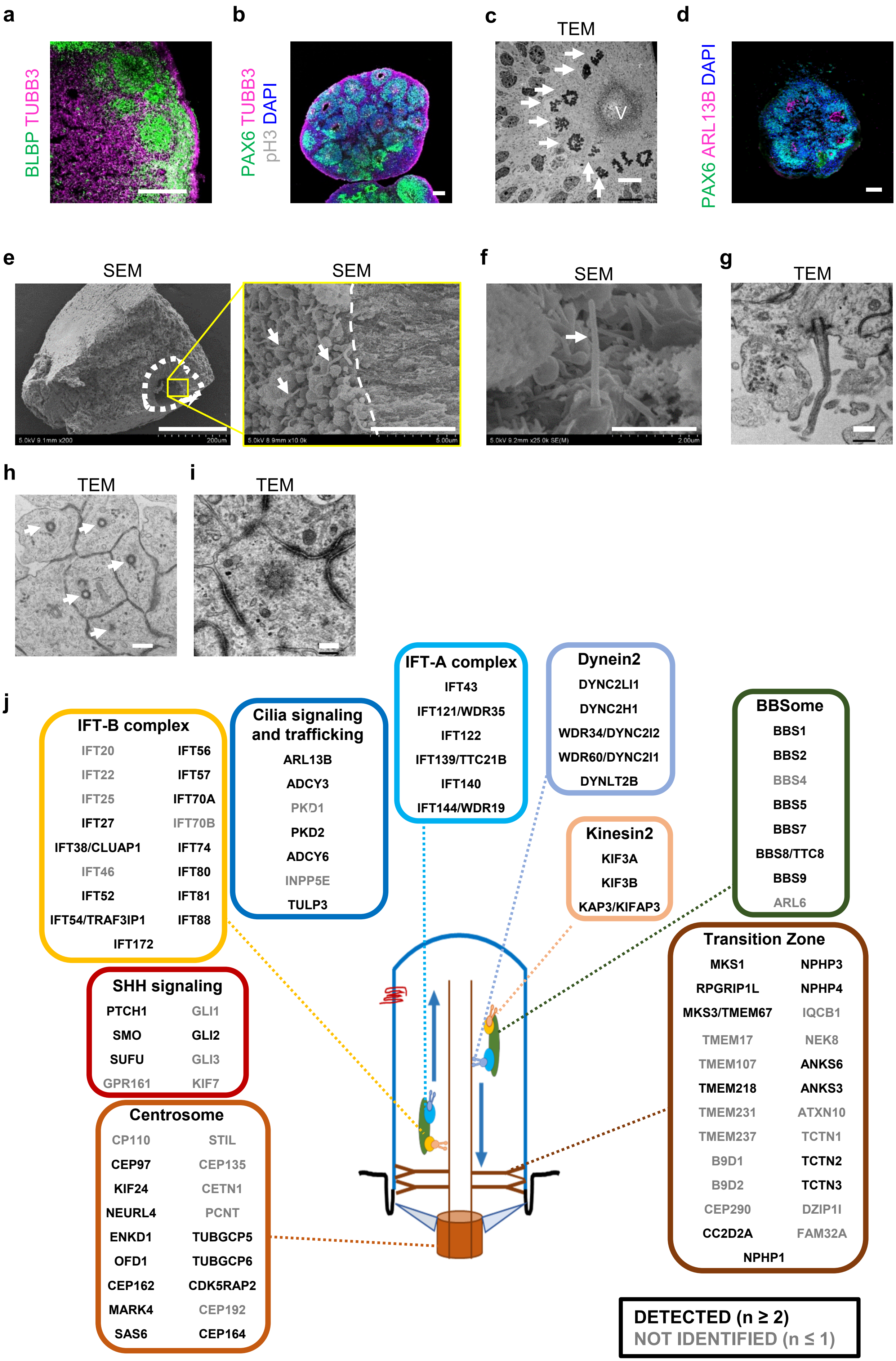
a
BLBP TUBB3
b
PAX6 TUBB3 pH3 DAPI
c
TEM
V
d
PAX6 ARL13B DAPI
e
SEM
SEM
f
SEM
g
TEM
h
TEM
i
TEM
j
IFT-B complex
IFT20 IFT56
IFT22 IFT57
IFT25 IFT70A
IFT27 IFT70B
IFT38/CLUAP1 IFT74
IFT46 IFT80
IFT52 IFT81
IFT54/TRAF3IP1 IFT88
IFT172
Cilia signaling and trafficking
ARL13B
ADCY3
PKD1
PKD2
ADCY6
INPP5E
TULP3
IFT-A complex
IFT43
IFT121/WDR35
IFT122
IFT139/TTC21B
IFT140
IFT144/WDR19
Dynein2
DYNC2LI1
DYNC2H1
WDR34/DYNC2I2
WDR60/DYNC2I1
DYNLT2B
Kinesin2
KIF3A
KIF3B
KAP3/KIFAP3
BBSome
BBS1
BBS2
BBS4
BBS5
BBS7
BBS8/TTC8
BBS9
ARL6
SHH signaling
PTCH1 GLI1
SMO GLI2
SUFU GLI3
GPR161 KIF7
Centrosome
CP110 STIL
CEP97 CEP135
KIF24 CETN1
NEURL4 PCNT
ENKD1 TUBGCP5
OFD1 TUBGCP6
CEP162 CDK5RAP2
MARK4 CEP192
SAS6 CEP164
Transition Zone
MKS1 NPHP3
RPGRIP1L NPHP4
MKS3/TMEM67 IQCB1
TMEM17 NEK8
TMEM107 ANKS6
TMEM218 ANKS3
TMEM231 ATXN10
TMEM237 TCTN1
B9D1 TCTN2
B9D2 TCTN3
CEP290 DZIP1l
CC2D2A FAM32A
NPHP1
DETECTED (n ≥ 2)
NOT IDENTIFIED (n ≤ 1)

Supplementary Fig. 1 Neural stem/progenitor cells (NPCs) have primary cilia in the ventricles of telencephalic organoids (**a**) BLBP-positive NPCs and β-tubulin III (TUBB3)-positive neurons. (**b**) pH3-positive mitotic cells were observed in the inner layer of PAX6-positive NPCs enriched in the ventricular zone. β-tubulin III (TUBB3)-positive neurons were observed around the ventricular zone. (**c**) Transmission electron microscopy (TEM) analysis revealed the existence of mitotic cells in the inner layer of the ventricular zone. Arrows indicate mitotic cells. (**d**) ARL13B-positive primary cilia were observed inside the ventricular zone enriched with PAX6-positive NPCs. (**e, f**) SEM analysis revealed a cilia-enriched region on the inner side of the ventricular zone of telencephalic organoids. White dotted lines indicate ventricular zones. Arrows indicate representative primary cilia. (**g**) TEM analysis revealed the existence of one primary cilium per NPC in the ventricular zone. (**h**, **i**) TEM analysis identified basal bodies (arrows) at the apical endfeet of NPCs in the ventricular zone. (**j**) Commonly known ciliary proteins identified in *cilia-EGFP-APEX2* organoids are shown. Black labels indicate detected in at least two samples and gray labels indicate detected in one or zero samples. Scale bars: (**a**) 200 μm, (**b**, **d**) 100 μm, (**c**) 10 μm, (**e**, left) 200 μm, (**e**, right) 5 μm, (**f**) 2 μm, (**g**, **h**) 500 nm and (**i**) 200 nm.

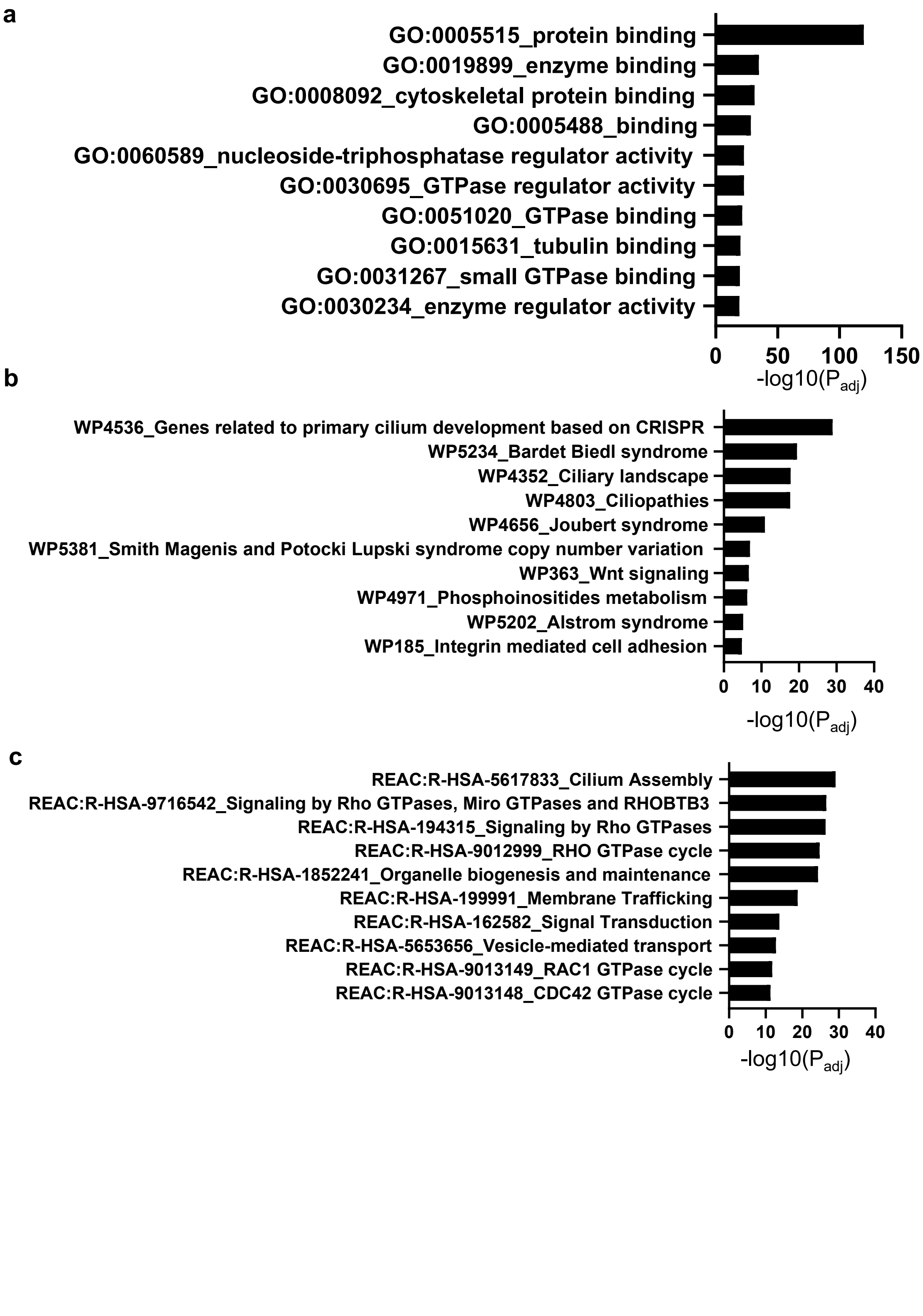
a
GO:0005515_protein binding
GO:0019899_enzyme binding
GO:0008092_cytoskeletal protein binding
GO:0005488_binding
GO:0060589_nucleoside-triphosphatase regulator activity
GO:0030695_GTPase regulator activity
GO:0051020_GTPase binding
GO:0015631_tubulin binding
GO:0031267_small GTPase binding
GO:0030234_enzyme regulator activity
0
50
100
150
-log10(P_adj)
b
WP4536_Genes related to primary cilium development based on CRISPR
WP5234_Bardet Biedl syndrome
WP4352_Ciliary landscape
WP4803_Ciliopathies
WP4656_Joubert syndrome
WP5381_Smith Magenis and Potocki Lupski syndrome copy number variation
WP363_Wnt signaling
WP4971_Phosphoinositides metabolism
WP5202_Alstrom syndrome
WP185_Integrin mediated cell adhesion
0
10
20
30
40
-log10(P_adj)
c
REAC:R-HSA-5617833_Cilium Assembly
REAC:R-HSA-9716542_Signaling by Rho GTPases, Miro GTPases and RHOBTB3
REAC:R-HSA-194315_Signaling by Rho GTPases
REAC:R-HSA-9012999_RHO GTPase cycle
REAC:R-HSA-1852241_Organelle biogenesis and maintenance
REAC:R-HSA-199991_Membrane Trafficking
REAC:R-HSA-162582_Signal Transduction
REAC:R-HSA-5653656_Vesicle-mediated transport
REAC:R-HSA-9013149_RAC1 GTPase cycle
REAC:R-HSA-9013148_CDC42 GTPase cycle
0
10
20
30
40
-log10(P_adj)

Supplementary Fig. 2 Proteomic analysis of ciliary components (**a**–**c**) GO term analysis using g:Profiler was performed on proteins detected in the *cilia-EGFP-APEX2* samples identified by proteomics. GO indicates Gene Ontology, REAC indicates Reactome and WP indicates Wikipathways. Source data are provided as a Source Data file.

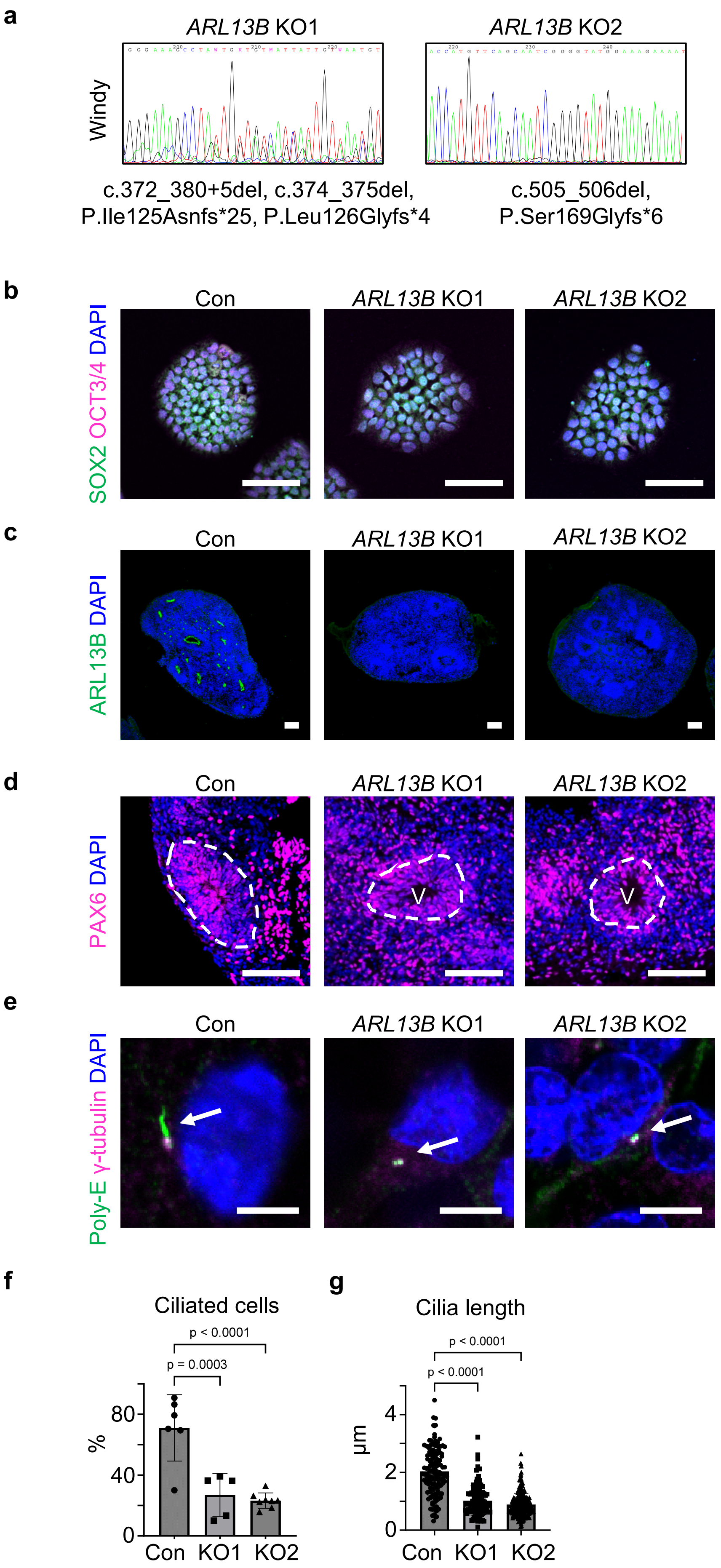

a
ARL13B KO1
ARL13B KO2
Windy
c.372_380+5del, c.374_375del,
P.Ile125Asnfs*25, P.Leu126Glyfs*4
c.505_506del,
P.Ser169Glyfs*6
b
Con
ARL13B KO1
ARL13B KO2
SOX2 OCT3/4 DAPI
c
ARL13B DAPI
d
PAX6 DAPI
V
e
Poly-E γ-tubulin DAPI
f
Ciliated cells
p < 0.0001
p = 0.0003
%
80
40
0
Con KO1 KO2
g
Cilia length
p < 0.0001
μm
4
2
0

Supplementary Fig. 3 *ARL13B* deletion induces shortening of primary cilia in neural stem/progenitor cells (NPCs) in 2D culture (**a**) Sanger sequencing of the CRISPR/Cas9-targeted loci in *ARL13B* KO1 and *ARL13B* KO2 iPS cells (Windy iPS cells). (**b**) SOX2 and OCT3/4 expression in control, *ARL13B* KO1 and *ARL13B* KO2 iPS cells. (**c**) ARL13B-positive primary cilia were seen in the ventricular zone of control, but not in *ARL13B* KO1 or *ARL13B* KO2 organoids. (**d**) PAX6-positive NPCs were observed in the ventricular zone of control, *ARL13B* KO1 and *ARL13B* KO2 organoids. V, ventricle. White dotted lines indicate ventricular zones. (**e**–**g**) Comparison of the percentage and length of ciliated cells using control, *ARL13B* KO1 and *ARL13B* KO2 NPCs in 2D culture. (**f**) n = 6, 5, 8 regions of interest in two independent experiments. One-way ANOVA with Sidak's test was performed. (**g**) n = 137, 108, 256 cilia in two independent experiments. One-way ANOVA with Sidak's test was performed. Source data are provided as a Source Data file. (**f** and **g**) Data are presented as mean values ± standard deviation. Scale bars: (**b**–**d**) 100 μm and (**e**) 5 μm.

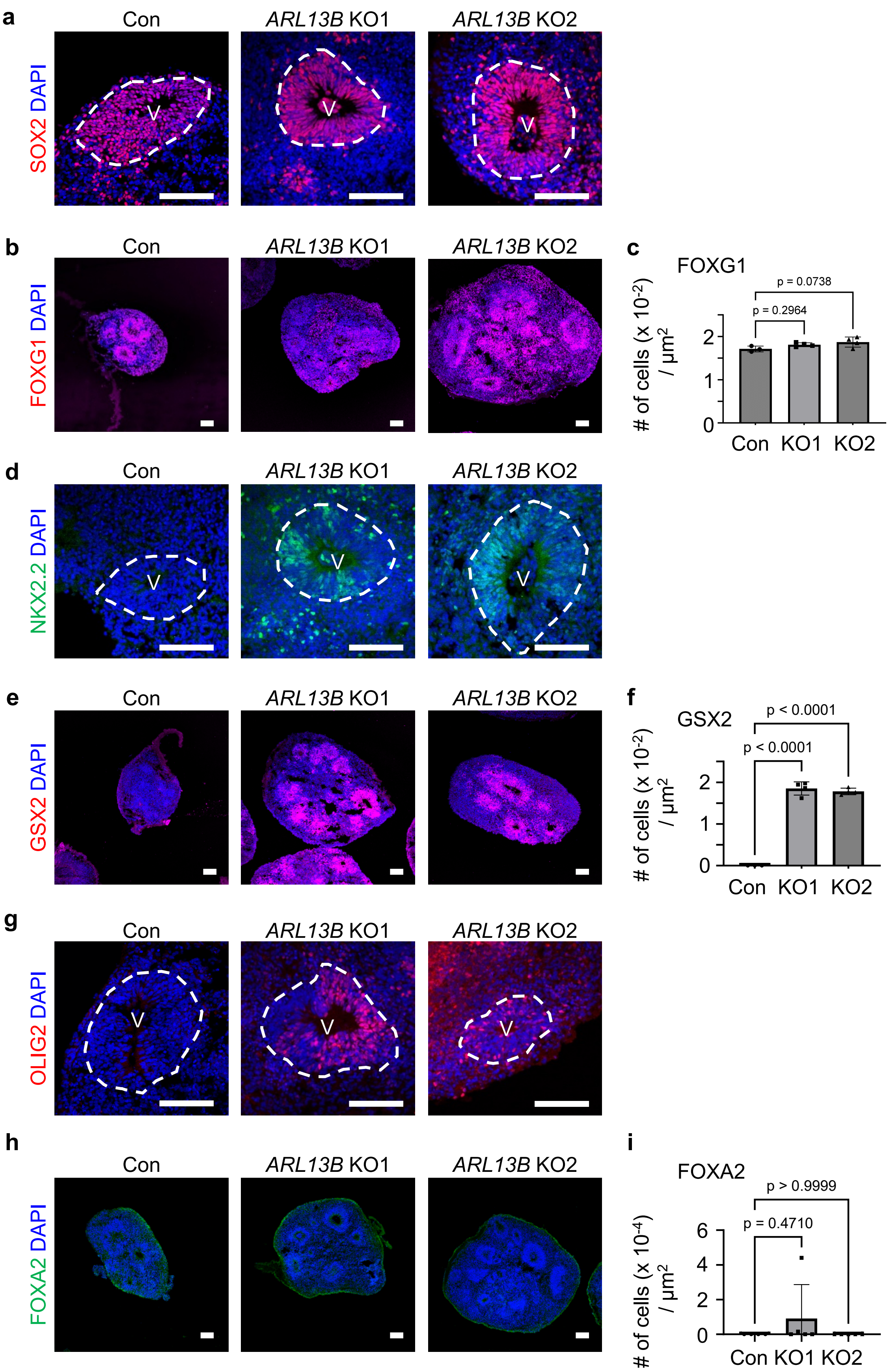
a
Con
ARL13B KO1
ARL13B KO2
SOX2 DAPI
V
b
FOXG1 DAPI
c
FOXG1
p = 0.0738
p = 0.2964
# of cells (x 10-2) / μm2
Con KO1 KO2
d
NKX2.2 DAPI
e
GSX2 DAPI
f
GSX2
p < 0.0001
p < 0.0001
g
OLIG2 DAPI
h
FOXA2 DAPI
i
FOXA2
p > 0.9999
p = 0.4710
# of cells (x 10-4) / μm2

Supplementary Fig. 4 *ARL13B* deletion induces ventralization in telencephalic organoids (**a**) SOX2-positive pan-neural stem/progenitor cells (NPCs) were observed in *ARL13B* KO1 and *ARL13B* KO2 organoids. White dotted lines indicate ventricular zones. V, ventricle. (**b**, **c**) FOXG1-positive telencephalic NPCs were observed in the ventricular zones of *ARL13B* KO1 and *ARL13B* KO2 organoids. *n* = 3, 4, 4 organoids in one independent experiment. One-way ANOVA with Sidak's test was performed. (**d**) NKX2.2-positive ventralized NPCs were observed in the ventricular zones of *ARL13B* KO1 and *ARL13B* KO2 organoids. White dotted lines indicate ventricular zones. V, ventricle. (**e**, **f**) GSX2-positive ventralized NPCs were observed in the ventricular zones of *ARL13B* KO1 and *ARL13B* KO2 organoids. *n* = 3, 4, 4 organoids in one independent experiment. One-way ANOVA with Sidak's test was performed. (**g**) OLIG2-positive ventralized NPCs were observed in the ventricular zones of *ARL13B* KO1 and *ARL13B* KO2 organoids. V, ventricle. White dotted lines indicate ventricular zones. (**h**, **i**) FOXA2-positive ventralized NPCs were rarely observed in the ventricular zones of *ARL13B* KO1 and *ARL13B* KO2 organoids. *n* = 4, 5, 5 organoids in three independent experiments. One-way ANOVA with Sidak's test was performed. Source data are provided as a Source Data file. (**c**, **f** and **i**) Data are presented as mean values ± standard deviation. Scale bars: (**a**, **b**, **d**, **e**, **g**, **h**) 100 µm.

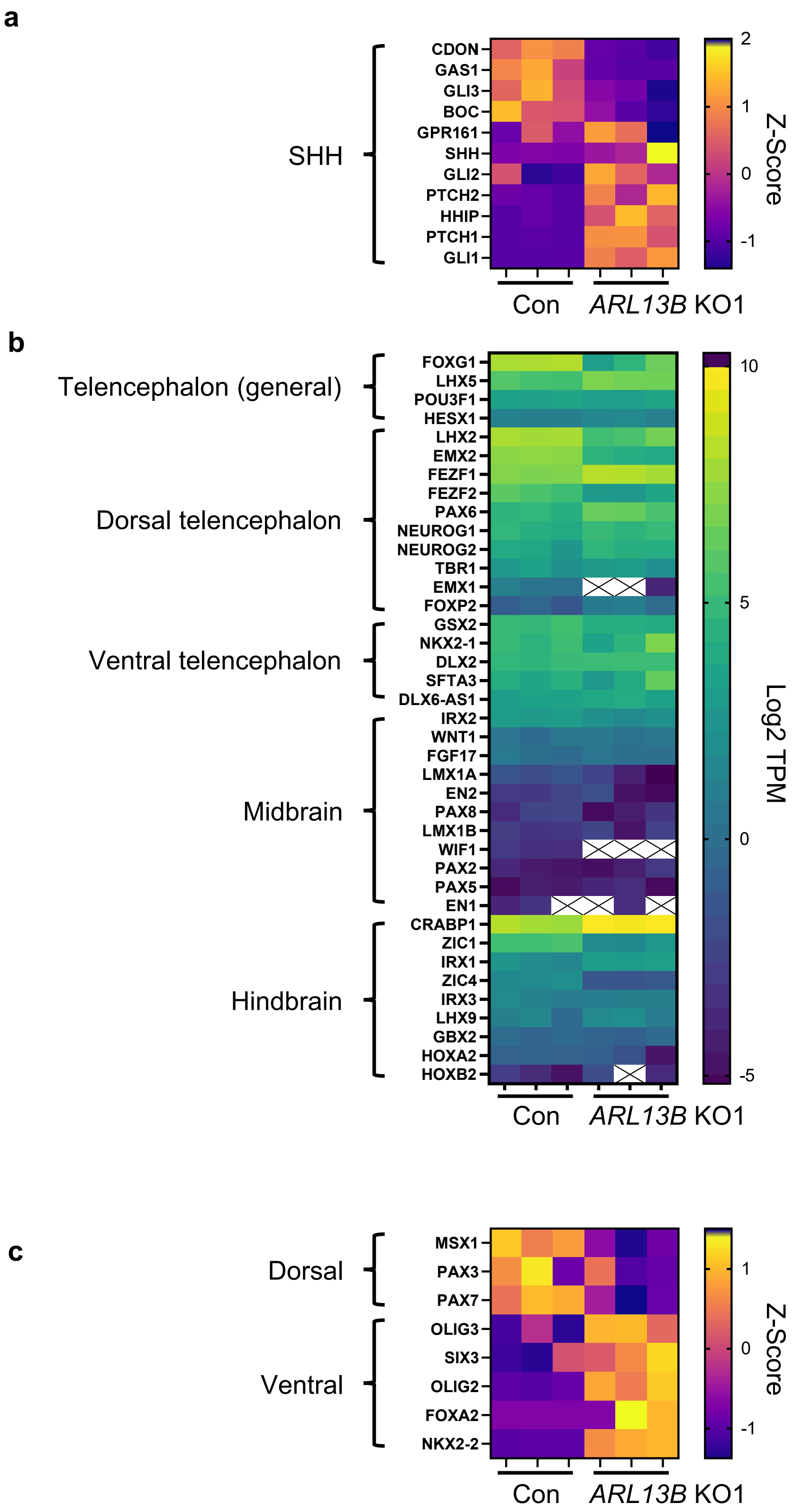
a
SHH
CDON
GAS1
GLI3
BOC
GPR161
SHH
GLI2
PTCH2
HHIP
PTCH1
GLI1
Z-Score
2
1
0
-1
Con
ARL13B KO1
b
Telencephalon (general)
FOXG1
LHX5
POU3F1
HESX1
Dorsal telencephalon
LHX2
EMX2
FEZF1
FEZF2
PAX6
NEUROG1
NEUROG2
TBR1
EMX1
FOXP2
Ventral telencephalon
GSX2
NKX2-1
DLX2
SFTA3
DLX6-AS1
Midbrain
IRX2
WNT1
FGF17
LMX1A
EN2
PAX8
LMX1B
WIF1
PAX2
PAX5
EN1
Hindbrain
CRABP1
ZIC1
IRX1
ZIC4
IRX3
LHX9
GBX2
HOXA2
HOXB2
Log2 TPM
10
5
0
-5
Con
ARL13B KO1
c
Dorsal
MSX1
PAX3
PAX7
Ventral
OLIG3
SIX3
OLIG2
FOXA2
NKX2-2
Z-Score
1
0
-1
Con
ARL13B KO1

Supplementary Fig. 5 RNA-seq analysis of *ARL13B* KO1 organoids reveals increased SONIC HEDGEHOG (SHH) signaling and altered brain regionalization

(**a**–**c**) Heatmap of RNA-seq data comparing control and *ARL13B* KO1 organoids. Genes are shown as (**a**, **c**) Z-score and (**b**) $\log_2$ TPM. White boxes with an X indicate no detection. Source data are provided as a Source Data file.

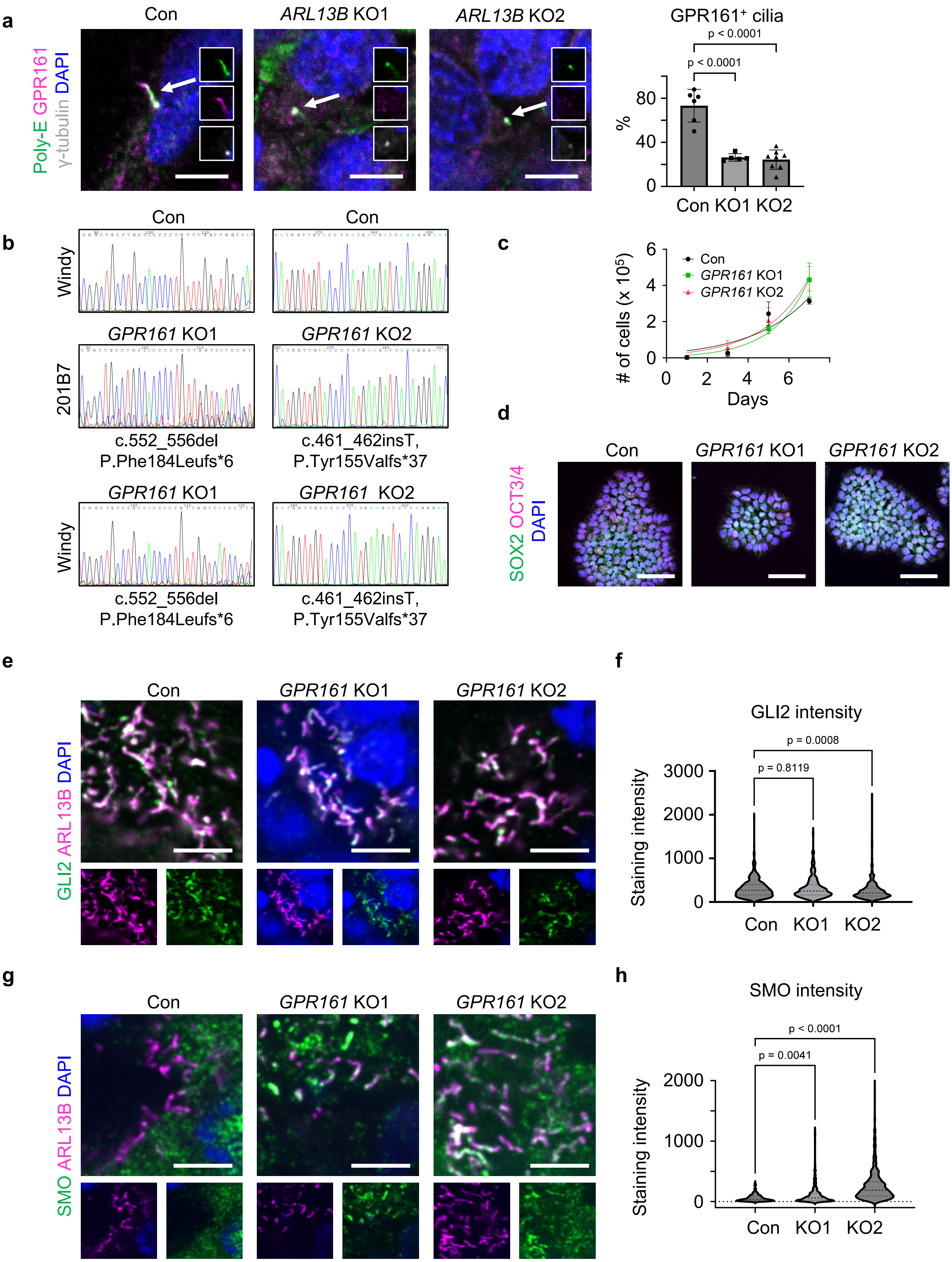
a
Con
ARL13B KO1
ARL13B KO2
Poly-E GPR161 γ-tubulin DAPI
GPR161⁺ cilia
p < 0.0001
p < 0.0001
%
80
40
0
Con KO1 KO2
b
Con
Con
Windy
GPR161 KO1
GPR161 KO2
201B7
c.552_556del
P.Phe184Leufs*6
c.461_462insT,
P.Tyr155Valfs*37
GPR161 KO1
GPR161 KO2
Windy
c.552_556del
P.Phe184Leufs*6
c.461_462insT,
P.Tyr155Valfs*37
c
Con
GPR161 KO1
GPR161 KO2
# of cells (x 10^5)
Days
d
Con
GPR161 KO1
GPR161 KO2
SOX2 OCT3/4 DAPI
e
Con
GPR161 KO1
GPR161 KO2
GLI2 ARL13B DAPI
f
GLI2 intensity
p = 0.0008
p = 0.8119
Staining intensity
3000
2000
1000
0
Con KO1 KO2
g
Con
GPR161 KO1
GPR161 KO2
SMO ARL13B DAPI
h
SMO intensity
p < 0.0001
p = 0.0041
Staining intensity
2000
1000
0
Con KO1 KO2

Supplementary Fig. 6 Deletion of *GPR161* affects SONIC HEDGEHOG signaling (**a**) Quantification of GPR161-positive cilia in control, *ARL13B* KO1 and *ARL13B* KO2 neural stem/progenitor cells (NPCs) in 2D culture shown in Supplementary Fig. 3e. Arrows indicate primary cilia. White boxes show magnified views of primary cilia in each channel. *n* = 6, 5, 8 regions of interest in two independent experiments. One-way ANOVA with Sidak's test was performed. (**b**) Sanger sequencing of CRISPR/Cas9-targeted loci in control, *GPR161* KO1 and *GPR161* KO2 iPS cells (201B7 cells and Windy iPS cells). (**c**) Cell growth curves of control, *GPR161* KO1 and *GPR161* KO2 iPS cells. *n* = 3 technical replicates in three independent experiments. Two-way ANOVA and Dunnett's multiple comparisons test were performed. *p* values for Con vs *GPR161* KO1, Con vs *GPR161* KO2 were 0.8934 and 0.2324, respectively. (**d**) SOX2 and OCT3/4 expression in control, *GPR161* KO1 and *GPR161* KO2 iPS cells. (**e**, **f**) GLI2 localization in the primary cilia of NPCs in telencephalic organoids was quantified. *n* = 560, 450, 515 cilia in one independent experiment. One-way ANOVA with Sidak's test was performed. (**g**, **h**) SMO localization in the primary cilia of NPCs in telencephalic organoids was quantified. *n* = 227, 436, 593 cilia in two independent experiments. One-way ANOVA with Sidak's test was performed. Source data are provided as a Source Data file. (**a** and **c**) Data are presented as mean values ± standard deviation. (**f** and **h**) Violin plots show the data distribution, with horizontal lines indicating the 25th percentile, median and 75th percentile. Scale bars: (**a**, **e**, **g**) 5 μm and (**d**) 100 μm.

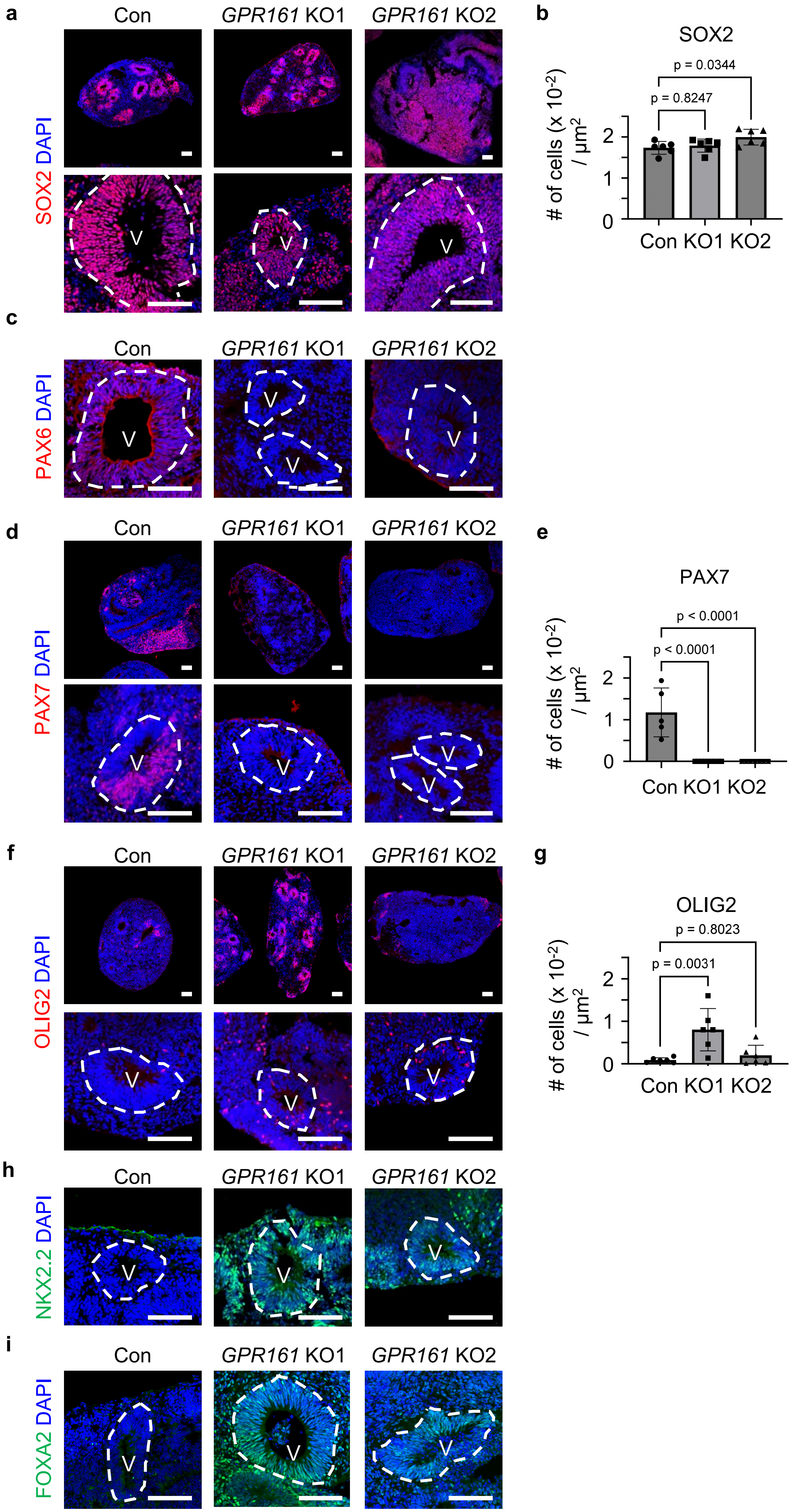
a
Con
GPR161 KO1
GPR161 KO2
SOX2 DAPI
V
b
SOX2
p = 0.0344
p = 0.8247
# of cells (x 10-2) / μm2
Con KO1 KO2
c
PAX6 DAPI
d
PAX7 DAPI
e
PAX7
p < 0.0001
f
OLIG2 DAPI
g
OLIG2
p = 0.8023
p = 0.0031
h
NKX2.2 DAPI
i
FOXA2 DAPI

Supplementary Fig. 7 *GPR161* deletion alters dorsal/ventral neural stem/progenitor cell marker patterns in the telencephalic organoids (**a**, **b**) SOX2-positive pan-neural stem/progenitor cells (NPCs) were observed in the ventricular zones of control, *GPR161* KO1 and *GPR161* KO2 organoids. White dotted lines indicate ventricular zones. V, ventricle. n = 6, 6, 6 organoids in one independent experiment. One-way ANOVA with Sidak's test was performed. (**c**) Reduced PAX6-positive dorsal NPCs in the ventricular zones of *GPR161* KO organoids. White dotted lines indicate ventricular zones. (**d**, **e**) Reduced PAX7-positive dorsal NPCs in the ventricular zones of *GPR161* KO organoids. n = 5, 9, 7 organoids in two independent experiments. One-way ANOVA with Sidak's test was performed. White dotted lines indicate ventricular zones. (**f**, **g**) Sparse OLIG2-positive NPCs in the ventricular zones of control, *GPR161* KO1 and *GPR161* KO2 organoids. n = 6, 6, 6 organoids in one independent experiment. One-way ANOVA with Sidak's test was performed. White dotted lines indicate ventricular zones. (**h**) Increased NKX2.2-positive ventral NPCs in the ventricular zones of *GPR161* KO organoids. White dotted lines indicate ventricular zones. (**i**) Increased FOXA2-positive ventral NPCs in the ventricular zones of *GPR161* KO organoids. White dotted lines indicate ventricular zones. Source data are provided as a Source Data file. (**b**, **e** and **g**) Data are presented as mean values ± standard deviation. Scale bars: (**a, c, d**, **f**, **h**, **i**) 100 µm.

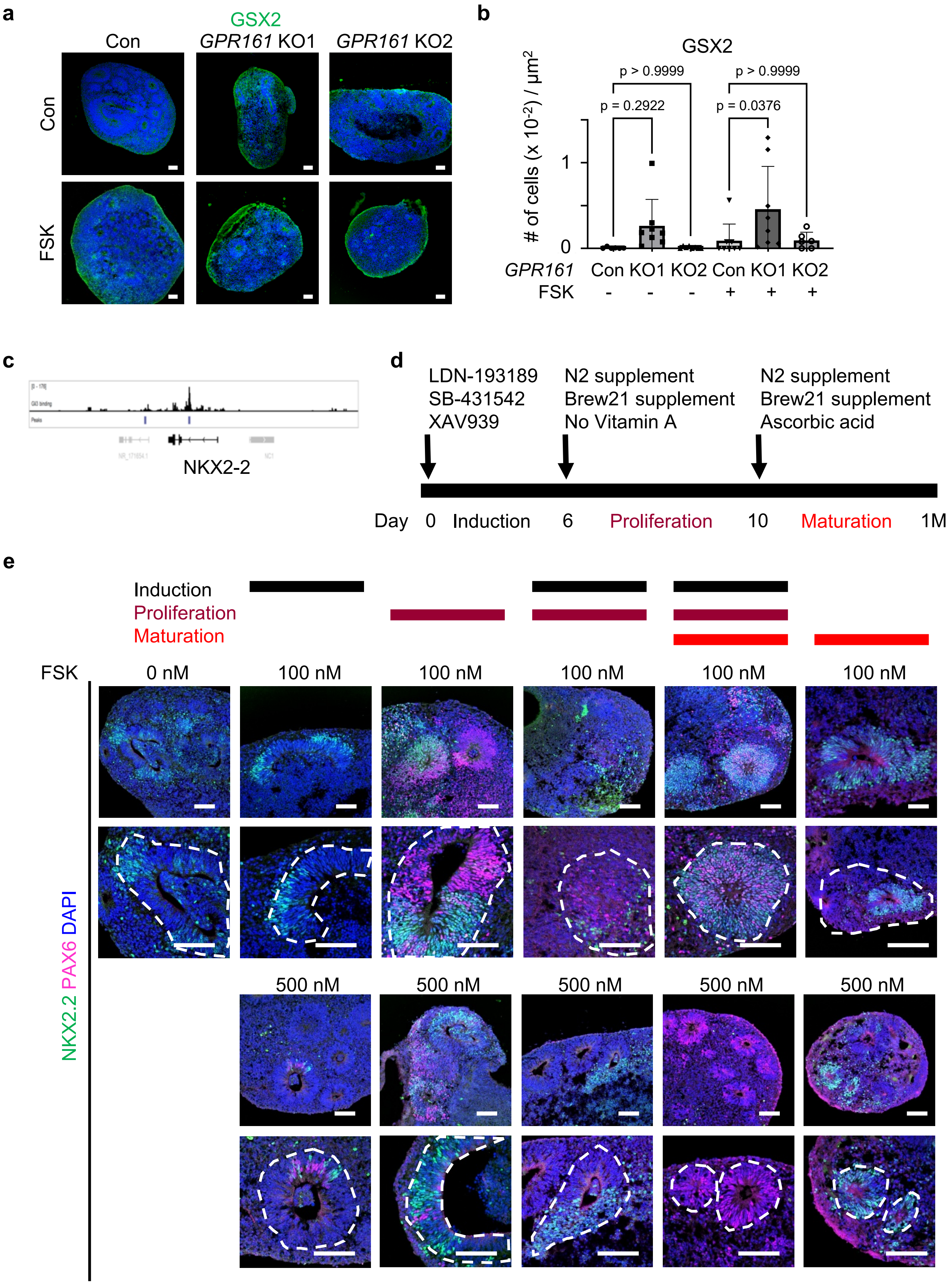
a
GSX2
Con
GPR161 KO1
GPR161 KO2
Con
FSK
b
GSX2
# of cells (x 10-2) / µm2
p > 0.9999
p > 0.9999
p = 0.2922
p = 0.0376
1
0
GPR161 Con KO1 KO2 Con KO1 KO2
FSK - - - + + +
c
NKX2-2
d
LDN-193189
SB-431542
XAV939
N2 supplement
Brew21 supplement
No Vitamin A
N2 supplement
Brew21 supplement
Ascorbic acid
Day 0 Induction 6 Proliferation 10 Maturation 1M
e
Induction
Proliferation
Maturation
FSK 0 nM 100 nM 100 nM 100 nM 100 nM 100 nM
500 nM 500 nM 500 nM 500 nM 500 nM
NKX2.2 PAX6 DAPI

Supplementary Fig. 8 Forskolin treatment schedule in *GPR161* KO organoids (**a**, **b**) GSX2 in the ventricular zones of *GPR161* KO organoids. *n* = 6, 8, 8, 8, 8, 6 organoids in 2 independent experiments. One-way ANOVA with Sidak's test was performed. FSK: forskolin. (**c**) CUT&Tag data of GLI3 binding in 23-day-old brain organoids from human iPS cell line showing the NKX2-2 gene region. Signal tracks (upper, black), called peaks (middle, blue), and genes (lower, gray and black) are shown. (**d**) Time schedule of telencephalic organoid generation. (**e**) Induction of dorsalization of neural stem/progenitor cells (NPCs) in *GPR161* KO1 organoids with various treatment schedules of FSK. Black, brown and pink lines indicate the period with forskolin. White dotted lines indicate ventricular zones. Source data are provided as a Source Data file. (**b**) Data are presented as mean values ± standard deviation. Scale bars: (**a**, **e**) 100 μm.

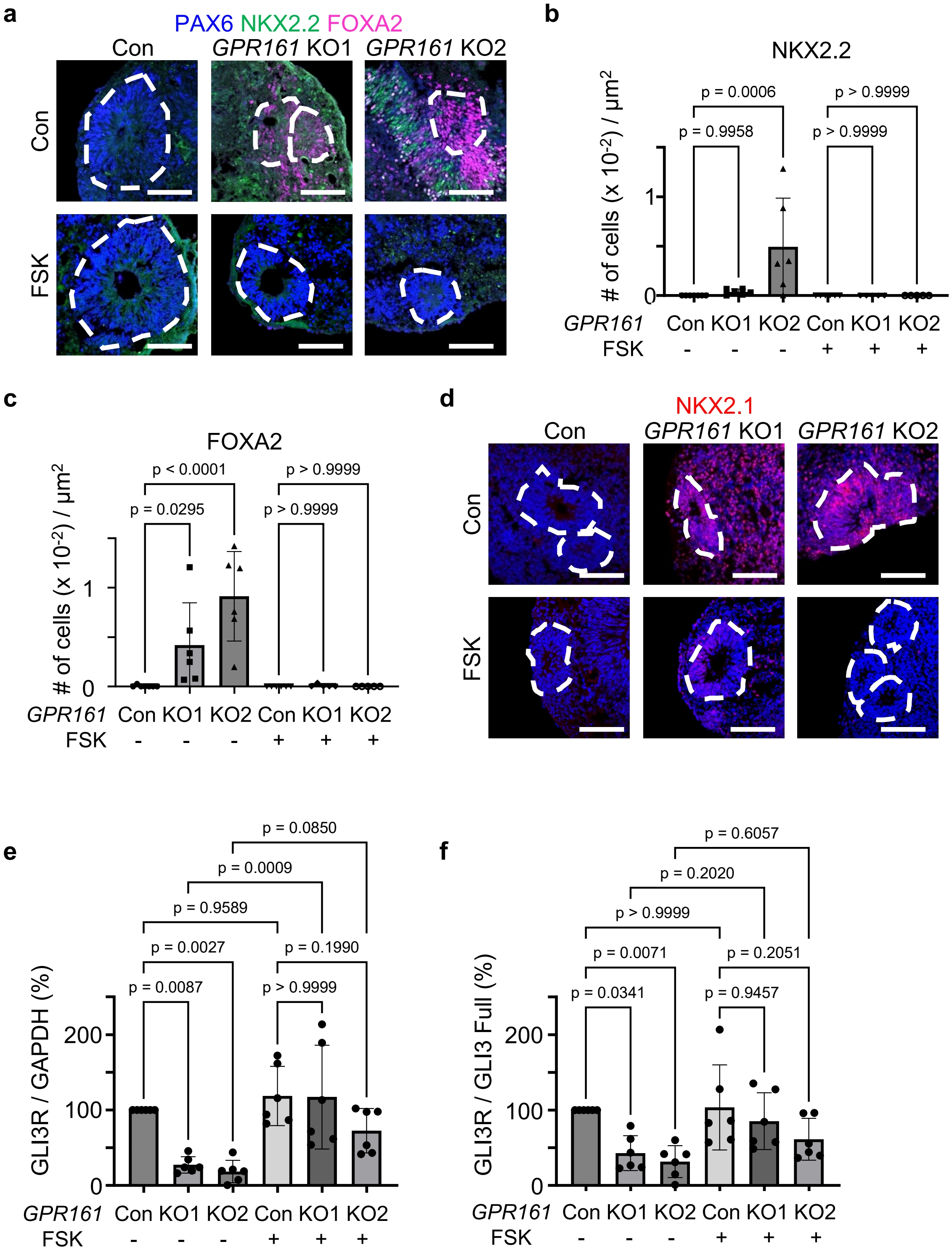
a
PAX6 NKX2.2 FOXA2
Con
GPR161 KO1
GPR161 KO2
Con
FSK
b
NKX2.2
p = 0.0006
p > 0.9999
p = 0.9958
p > 0.9999
# of cells (x 10-2) / µm2
GPR161 Con KO1 KO2 Con KO1 KO2
FSK - - - + + +
c
FOXA2
p < 0.0001
p > 0.9999
p = 0.0295
p > 0.9999
# of cells (x 10-2) / µm2
GPR161 Con KO1 KO2 Con KO1 KO2
FSK - - - + + +
d
NKX2.1
Con
GPR161 KO1
GPR161 KO2
Con
FSK
e
p = 0.0850
p = 0.0009
p = 0.9589
p = 0.0027
p = 0.1990
p = 0.0087
p > 0.9999
GLI3R / GAPDH (%)
GPR161 Con KO1 KO2 Con KO1 KO2
FSK - - - + + +
f
p = 0.6057
p = 0.2020
p > 0.9999
p = 0.0071
p = 0.2051
p = 0.0341
p = 0.9457
GLI3R / GLI3 Full (%)
GPR161 Con KO1 KO2 Con KO1 KO2
FSK - - - + + +

Supplementary Fig. 9 Forskolin treatment partially restores dorsal identity of *GPR161* KO organoids (**a**–**d**) Forskolin (FSK) treatment restored dorsal identity of neural stem/progenitor cells (NPCs) in *GPR161* KO organoids. Analysis of (**a**–**c**) PAX6, NKX2.2, FOXA2 and (**d**) NKX2.1 in the ventricular zones. White dotted lines indicate ventricular zones. (**b**, **c**) *n* = 7, 6, 6, 6, 5, 5 organoids in two independent experiments. One-way ANOVA with Sidak's test was performed. (**e**, **f**) Western blot analysis of the GLI3 repressor (GLI3R) in *GPR161* KO organoids. GAPDH is a loading control. *n* = 6, 6, 6, 6, 6, 6 pools of organoids in three independent experiments. One-way ANOVA with Sidak's test was performed. Scale bars: (**a** and **d**) 100 μm. Source data are provided as a Source Data file. (**b**, **c**, **e** and **f**) Data are presented as mean values ± standard deviation.

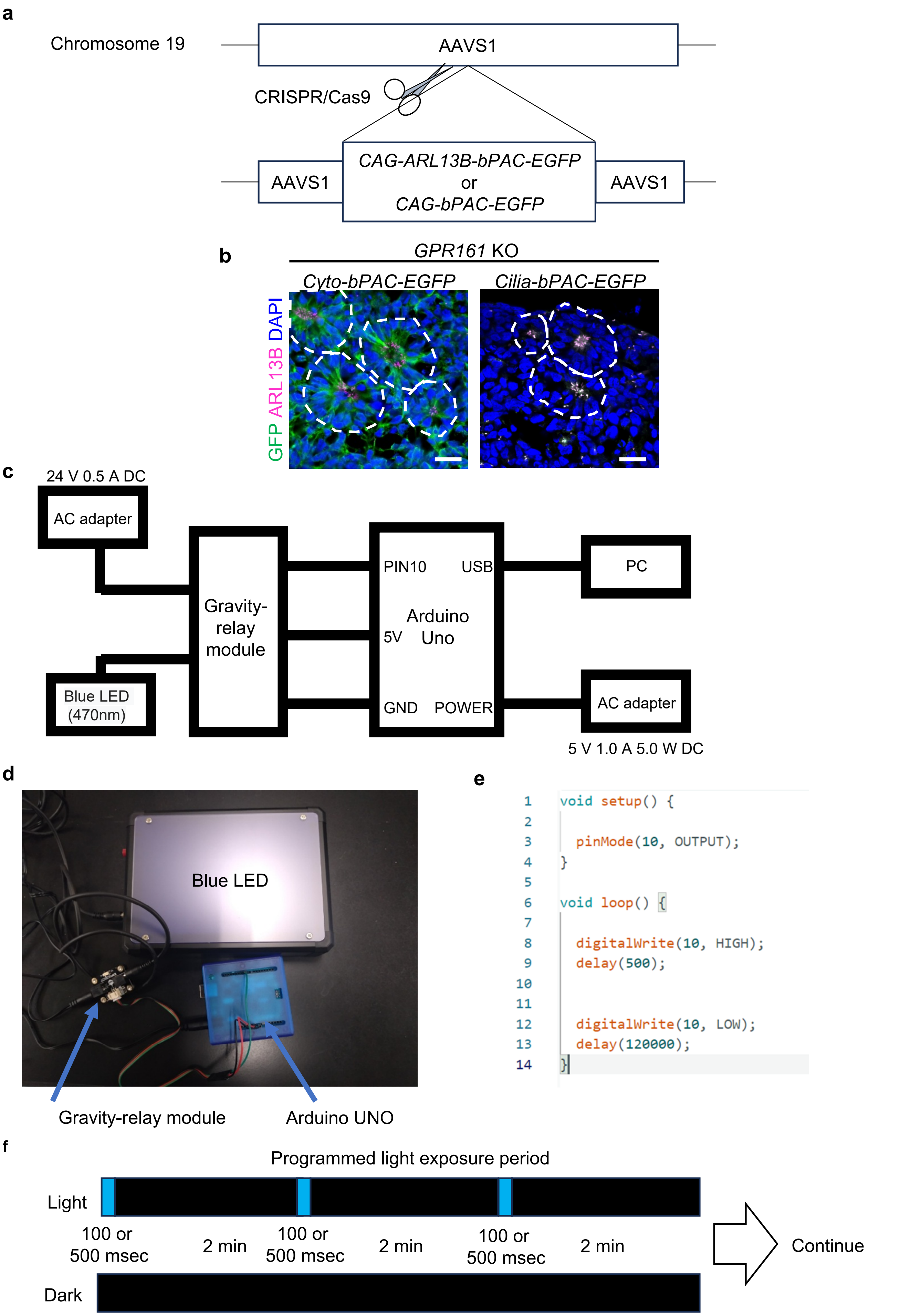
a
Chromosome 19
AAVS1
CRISPR/Cas9
AAVS1
CAG-ARL13B-bPAC-EGFP
or
CAG-bPAC-EGFP
AAVS1
b
GPR161 KO
Cyto-bPAC-EGFP
Cilia-bPAC-EGFP
GFP ARL13B DAPI
c
24 V 0.5 A DC
AC adapter
Gravity-relay module
PIN10
USB
Arduino Uno
5V
GND
POWER
PC
AC adapter
Blue LED (470nm)
5 V 1.0 A 5.0 W DC
d
Blue LED
Gravity-relay module
Arduino UNO
e
void setup() {
pinMode(10, OUTPUT);
}
void loop() {
digitalWrite(10, HIGH);
delay(500);
digitalWrite(10, LOW);
delay(120000);
}
f
Programmed light exposure period
Light
100 or 500 msec
2 min
100 or 500 msec
2 min
100 or 500 msec
2 min
Continue
Dark

Supplementary Fig. 10 Setup for optogenetic research (**a**) Scheme to generate gene knock-in at the safe harbor of chromosome 19. *CAG-bPAC-EGFP* or *CAG-ARL13B-bPAC-EGFP* was inserted into the *AAVS1* safe-harbor locus on chromosome 19 by CRISPR/Cas9. (**b**) GFP signals in the ventricular zone of *cyto-bPAC-EGFP*- and *cilia-bPAC-EGFP*-expressing *GPR161* KO organoids. Cilia-bPAC-EGFP was observed in ARL13B-positive primary cilia. White dotted lines indicate ventricular zones. (**c**, **d**) The circuit and devices used in the current study. (**e**) Arduino code for the current study. (**f**) A scheme of light exposure schedule. Scale bars: (**b**) 20 μm.

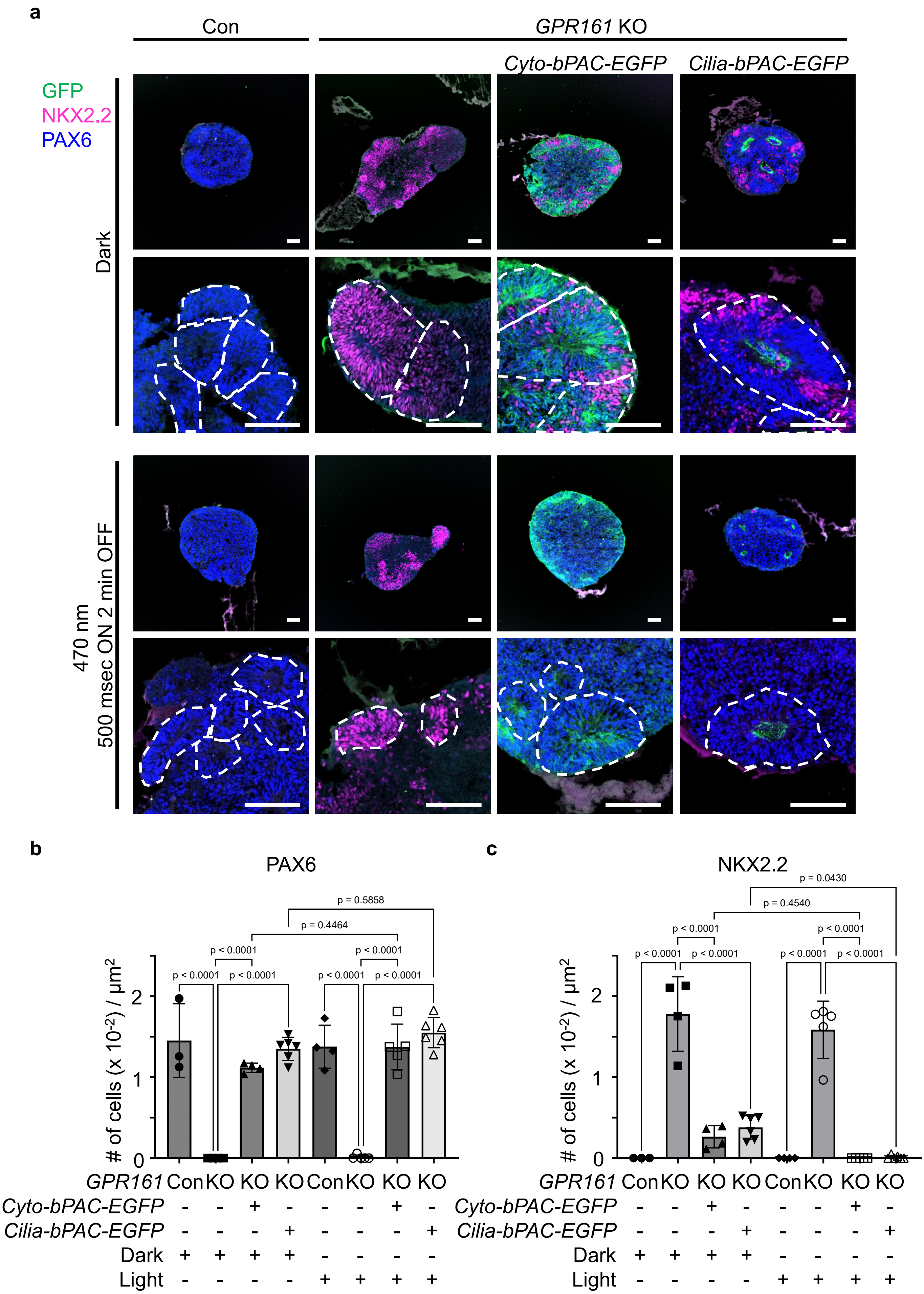
a
Con
GPR161 KO
Cyto-bPAC-EGFP
Cilia-bPAC-EGFP
GFP
NKX2.2
PAX6
Dark
470 nm
500 msec ON 2 min OFF
b
PAX6
# of cells (x 10-2) / µm2
p = 0.5858
p = 0.4464
p < 0.0001
p < 0.0001
p < 0.0001
p < 0.0001
p < 0.0001
p < 0.0001
p < 0.0001
GPR161 Con KO KO KO Con KO KO KO
Cyto-bPAC-EGFP - - + - - - + -
Cilia-bPAC-EGFP - - - + - - - +
Dark + + + + - - - -
Light - - - - + + + +
c
NKX2.2
p = 0.0430
p = 0.4540
p < 0.0001
p < 0.0001
p < 0.0001
p < 0.0001
p < 0.0001
p < 0.0001
GPR161 Con KO KO KO Con KO KO KO
Cyto-bPAC-EGFP - - + - - - + -
Cilia-bPAC-EGFP - - - + - - - +
Dark + + + + - - - -
Light - - - - + + + +

Supplementary Fig. 11 Optogenetic elevation of cytoplasmic or ciliary cAMP restores dorsal identity of *GPR161* KO organoids (**a**) Enhancement of either cytoplasmic cAMP levels or ciliary cAMP levels restored dorsal identity of *cyto-bPAC-EGFP*- or *cilia-bPAC-EGFP-GPR161* KO organoids following programmed light exposure (0.5 sec on, 2 min off cycle). Notably, PAX6-positive NPCs were present and NKX2.2-positive NPCs were reduced even in the dark, indicating leaky bPAC activity. White dotted lines indicate ventricular zones. (**b**, **c**) The numbers of PAX6-positive and NKX2.2-positive NPCs were shown in the ventricular zones of *cyto-bPAC-EGFP*- or *cilia-bPAC-EGFP-GPR161* KO organoids. *n* = 3, 4, 4, 6, 4, 5, 5, 6 organoids in two independent experiments. One-way ANOVA with Sidak's test was performed. Source data are provided as a Source Data file. (**b** and **c**) Data are presented as mean values ± standard deviation. Scale bar: (**a**) 100 µm.

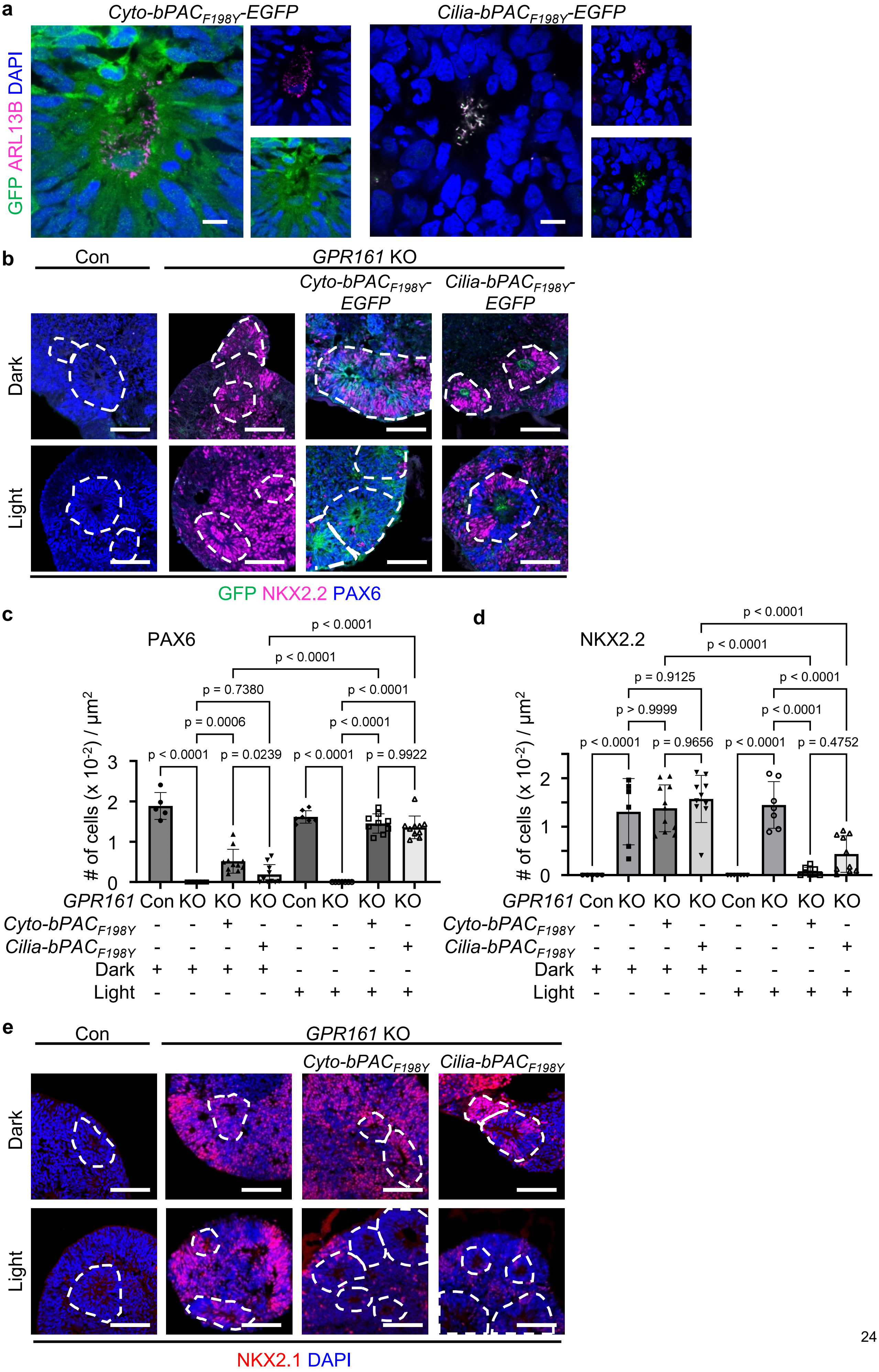

a
Cyto-bPAC$_{F198Y}$-EGFP
Cilia-bPAC$_{F198Y}$-EGFP
GFP ARL13B DAPI
b
Con
GPR161 KO
Cyto-bPAC$_{F198Y}$-EGFP
Cilia-bPAC$_{F198Y}$-EGFP
Dark
Light
GFP NKX2.2 PAX6
c
PAX6
# of cells (x 10^{-2}) / µm^2
p < 0.0001
p = 0.0006
p = 0.7380
p = 0.0239
p < 0.0001
p < 0.0001
p < 0.0001
p < 0.0001
p = 0.9922
p < 0.0001
GPR161 Con KO KO KO Con KO KO KO
Cyto-bPAC$_{F198Y}$ - - + - - - + -
Cilia-bPAC$_{F198Y}$ - - - + - - - +
Dark + + + + - - - -
Light - - - - + + + +
d
NKX2.2
# of cells (x 10^{-2}) / µm^2
p < 0.0001
p > 0.9999
p = 0.9656
p = 0.9125
p < 0.0001
p < 0.0001
p = 0.4752
p < 0.0001
p < 0.0001
p < 0.0001
GPR161 Con KO KO KO Con KO KO KO
Cyto-bPAC$_{F198Y}$ - - + - - - + -
Cilia-bPAC$_{F198Y}$ - - - + - - - +
Dark + + + + - - - -
Light - - - - + + + +
e
Con
GPR161 KO
Cyto-bPAC$_{F198Y}$
Cilia-bPAC$_{F198Y}$
Dark
Light
NKX2.1 DAPI

Supplementary Fig. 12 The effects of cAMP on dorsal/ventral patterning of *GPR161* KO organoids (**a**) GFP signals in the ventricular zone of *cyto-bPAC$_{F198Y}$-EGFP*- and *cilia-bPAC$_{F198Y}$-EGFP*-expressing *GPR161* KO organoids. Cilia-bPAC$_{F198Y}$-EGFP was observed in ARL13B-positive primary cilia. (**b**–**e**) Enhancement of either cytoplasmic cAMP levels or ciliary cAMP levels restored the numbers of PAX6, NKX2.2 and NKX2.1-positive neural stem/progenitor cells (NPCs) of *cyto-bPAC$_{F198Y}$-EGFP*- and *cilia-bPAC$_{F198Y}$-EGFP*-expressing *GPR161* KO organoids following programmed light exposure (0.1 sec on, 2 min off). White dotted lines indicate ventricular zones. n = 5, 6, 11, 10, 7, 7, 9, 10 organoids in four independent experiments. One-way ANOVA with Sidak's test was performed. Source data are provided as a Source Data file. (**c** and **d**) Data are presented as mean values ± standard deviation. Scale bar: (**a**) 5 μm and (**b**, **e**) 100 μm.

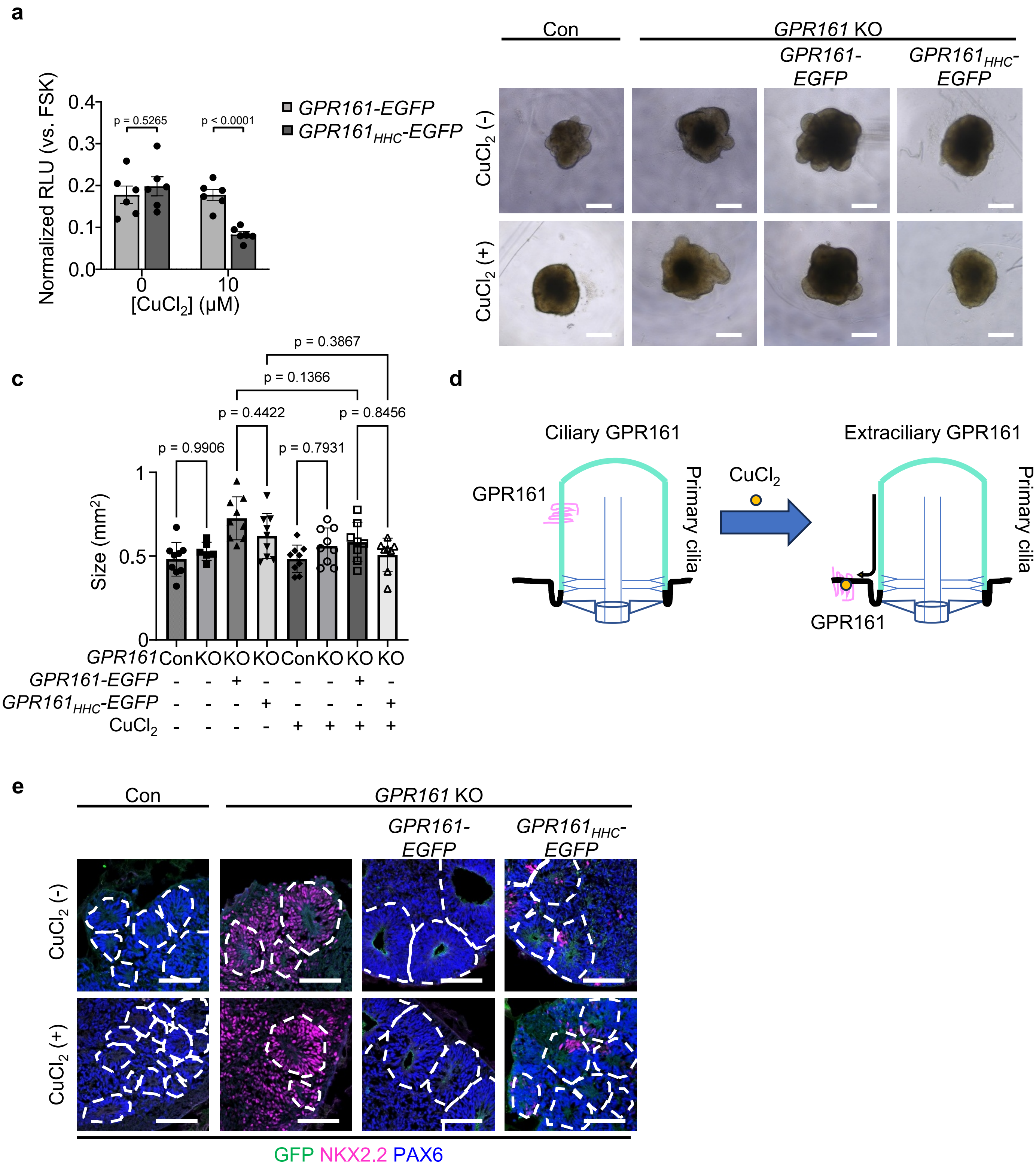
a
Normalized RLU (vs. FSK)
0.4
0.3
0.2
0.1
0.0
p = 0.5265
p < 0.0001
0
10
[CuCl2] (μM)
GPR161-EGFP
GPR161HHC-EGFP
Con
GPR161 KO
GPR161-EGFP
GPR161HHC-EGFP
CuCl2 (-)
CuCl2 (+)
c
p = 0.3867
p = 0.1366
p = 0.4422
p = 0.8456
p = 0.9906
p = 0.7931
Size (mm2)
1
0.5
0
GPR161 Con KO KO KO Con KO KO KO
GPR161-EGFP - - + - - - + -
GPR161HHC-EGFP - - - + - - - +
CuCl2 - - - - + + + +
d
Ciliary GPR161
Extraciliary GPR161
GPR161
Primary cilia
CuCl2
GPR161
Primary cilia
e
Con
GPR161 KO
GPR161-EGFP
GPR161HHC-EGFP
CuCl2 (-)
CuCl2 (+)
GFP NKX2.2 PAX6

Supplementary Fig. 13 Copper(II) chloride does not affect organoid size (**a**) Copper(II) chloride treatment induced a reduction in cAMP levels in *GPR161$_{HHC}$-EGFP-transfected HEK293 cells*, but not *GPR161-EGFP*-transfected HEK293 cells. 0 μM: *n* = 6, 6 independent experiments. 10 μM: *n* = 6, 6 independent experiments. Unpaired *t*-test with Welch's correction was performed. RLU indicates relative luminescence units compared to the FSK-treated control. FSK indicates forskolin. (**b**, **c**) Copper(II) chloride treatment did not affect the size of telencephalic organoids at 2 weeks. *n* = 9, 6, 9, 9, 9, 9, 8, 8 organoids in three independent experiments. One-way ANOVA with Sidak's test was performed. (**d**) A scheme showing that a metal ion induces ciliary exit of GPR161. (**e**) Copper(II) chloride increased NKX2.2-positive neural stem/progenitor cells (NPCs) but did not change PAX6-positive NPCs in organoids derived from *GPR161$_{HHC}$-EGFP*–expressing *GPR161* KO iPS cells, but not in organoids derived from *GPR161-EGFP*–expressing *GPR161* KO iPS cells. White dotted lines indicate ventricular zones. Source data are provided as a Source Data file. (**a**) Data are presented as mean values ± standard error of the mean. (**c**) Data are presented as mean values ± standard deviation. Scale bars: (**b**) 400 μm and (**e**) 100 μm.

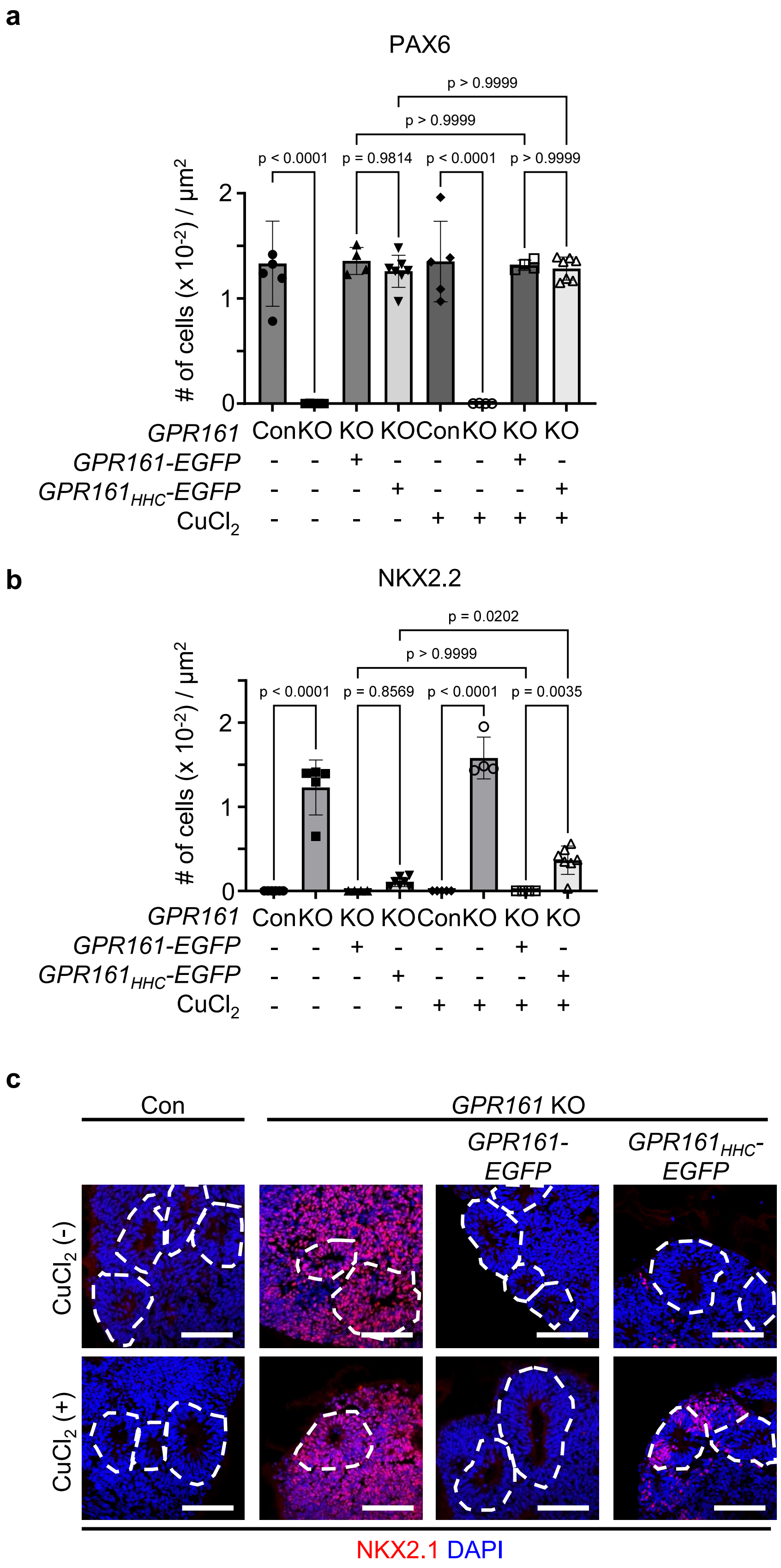
a
PAX6
p > 0.9999
p > 0.9999
p < 0.0001
p = 0.9814
p < 0.0001
p > 0.9999
# of cells (x 10-2) / μm2
GPR161
GPR161-EGFP
GPR161HHC-EGFP
CuCl2
Con KO KO KO Con KO KO KO
b
NKX2.2
p = 0.0202
p > 0.9999
p < 0.0001
p = 0.8569
p < 0.0001
p = 0.0035
c
Con
GPR161 KO
GPR161-EGFP
GPR161HHC-EGFP
CuCl2 (-)
CuCl2 (+)
NKX2.1 DAPI

Supplementary Fig. 14 Ciliary exit of GPR161 by coordination chemogenetics regulates the fate of dorsal/ventral neural stem/progenitor cells (NPCs) (**a**–**c**) Copper(II) chloride did not affect PAX6-positive NPCs, but increased NKX2.1-positive and NKX2.2-positive NPCs in *GPR161*$_{HHC}$*-EGFP*–expressing *GPR161* KO organoids, but not in *GPR161-EGFP*–expressing *GPR161* KO organoids. White dotted lines indicate ventricular zones. *n* = 6, 5, 4, 7, 5, 4, 4, 7 organoids in three independent experiments. One-way ANOVA with Sidak's test was performed. Source data are provided as a Source Data file. (**a** and **b**) Data are presented as mean values ± standard deviation. Scale bars: (**c**) 100 μm.

Supplementary Table 1		
Oligonucleotides	Source	Sequences (5' to 3')
ARL13B crRNA-1 (Design ID: Hs.Cas9.ARL13B.1.AA)	Integrated DNA Technologies	GATATCGGGAAAGCCTATAT
ARL13B crRNA-2 (Design ID: Hs.Cas9.ARL13B.1.AC)	Integrated DNA Technologies	AACCATGTTCAGCAATCTCG
GPR161 crRNA-1 (Design ID: Hs.Cas9.GPR161.1.AC)	Integrated DNA Technologies	CCACGGATGACCAACCAAAC
GPR161 crRNA-2 (Design ID: Hs.Cas9.GPR161.1.AE)	Integrated DNA Technologies	GATCTTCATGGGGTACACCA
sgArl13B-AA-F1	Integrated DNA Technologies	CTTTGACTTGGGAGGTGGAA
sgArl13B-AA-R1	Integrated DNA Technologies	CTTTAGTGGCCAATGCCATC
sgArl13B-AC-F2	Integrated DNA Technologies	TTGCAGTGCTATGTTTTGGAA
sgArl13B-AC-R2	Integrated DNA Technologies	AATTTTCGCACTCGTTCAGC
sgGpr161-F	Integrated DNA Technologies	CCCTCCATCCCTTTTCTAGC
sgGpr161-R	Integrated DNA Technologies	CTCTGAGCATCCTCCTCCAC
GPR161ACseq primer-R1	Integrated DNA Technologies	CCATAGCACACCAGCATGAC
beta-actin-F	Integrated DNA Technologies	GATCTGGCACCACACCTTCT
beta-actin-R	Integrated DNA Technologies	GGGGTGTTGAAGGTCTCAAA
hGli1-F	Integrated DNA Technologies	AGCGTGAGCCTGAATCTGTG
hGli1-R	Integrated DNA Technologies	CAGCATGTACTGGGCTTTGAA
Mycoplasma-F	Trujillo et al	GGCGAATGGGTGAGTAAC
Mycoplasma-R	Trujillo et al	CGGATAACGCTTGCGACCT
Antibodies	Source	Identifier
Acetylated alpha-tubulin (6-11B-1) Dilution 1:500	Sigma-Aldrich	Cat. # T6793, RRID:AB_477585 Lot. 017M4806U
ARL13B Dilution 1:500	Proteintech	Cat. # 17711-1-AP, RRID:AB_2060867 Lot. 00077350
ARL13B Dilution 1:500	BioLegend	Cat. # 857602, RRID:AB_2801216 Lot. B312353
Beta-actin (AC-15) Dilution 1:2500	Santa Cruz	Cat. # sc-69879, RRID:AB_1119529 Lot. # F0321
Beta-catenin Dilution 1:500	Sigma-Aldrich	Cat. # C2206, RRID:AB_476831
Beta-tubulin III (TUBB3) Dilution 1:500	Santa Cruz	Cat. # sc-80005, RRID:AB_2210816 Lot. # K1419
BLBP Dilution 1:500	Millipore	Cat. # ABN14, RRID:AB_10000325
FOXA2 (HNF-3beta; RY-7) Dilution 1:500	Santa Cruz	Cat. # sc-101060, RRID:AB_1124660 Lot. # H0620
FOXG1 Dilution 1:500	Abcam	Cat. # ab18259, RRID:AB_732415 Lot. 1029807-15
Gamma-tubulin (C-11) Dilution 1:500	Santa Cruz	Cat. # sc-17787, RRID:AB_628417 Lot. # J2518
Gamma-tubulin (TU-30) Dilution 1:500	Santa Cruz	Cat. # sc-51715, RRID:AB_630410 Lot. # L0717
GAPDH Dilution 1:2500	Novus	Cat. # NB300-322, RRID:AB_10001458 Lot. A4
GFP (B-2) Dilution 1:500	Santa Cruz	Cat. # sc-9996, RRID:AB_627695 Lot. # G0920
GLI2 Dilution 1:500	R&D Systems	Cat. # AF3526, RRID:AB_2279108 Lot. XQV0122021
GLI3 Dilution 1:2500	R&D Systems	Cat. # AF3690, RRID:AB_2232499 Lot. YLD0322051
GPR161 Dilution 1:500	Sigma-Aldrich	Cat. # AV42354, RRID:AB_1849281 Lot. 00055461
GPR161 Dilution 1:500	Proteintech	Cat. # 13398-1-AP, RRID:AB_2113965 Lot. 4275568
GSX2 Dilution 1:500	Sigma-Aldrich	Cat. # ABN162, RRID:AB_11203296 Lot. 4275568
IFT88 Dilution 1:500	Proteintech	Cat. # 13967-1-AP, RRID:AB_2121979 Lot. 00082017
NKX2.1 (TTF-1; 8G7G3/1) Dilution 1:500	Diagnostic BioSystems	Cat. # MOB285-01, RRID:AB_3730160 Lot. X204-QD-M
NKX2.2 Dilution 1:20	Hybridoma Bank	Cat. # 74.5A5, RRID:AB_531794
Oct3/4 (C-10) Dilution 1:500	Santa Cruz	Cat. # sc-5279, RRID:AB_628051 Lot. #F0920
Olig2 Dilution 1:500	R&D Systems	Cat. # AF2418, RRID:AB_2157554 Lot. UPA0921011
PAX6 Dilution 1:500	BioLegend	Cat. # 901301, RRID:AB_2565003
PAX7 Dilution 1:20	Hybridoma Bank	Cat. # Pax7, RRID:AB_2299243
p-Histone H3 (C-2) Dilution 1:1000	Santa Cruz	Cat. # sc-374669, RRID:AB_11150094 Lot. # H2019
Poly-E (GT335) Dilution 1:1000	Adipogen	Cat. # AG-20B-0020, RRID:AB_2335608 Lot. A44052307
SMO (E-5) Dilution 1:500	Santa Cruz	Cat. # sc-166685, RRID:AB_2239686 Lot. # H1419
SOX2 (E-4) Dilution 1:500	Santa Cruz	Cat. # sc-365823, RRID:AB_10842165 Lot. # C1622
Goat anti-Rabbit Secondary Antibody, Alexa Fluor 488 Dilution 1:500	Invitrogen	Cat. # A-11008, RRID:AB_143165 Lot. 2179202
Goat anti-Rabbit Secondary Antibody, Alexa Fluor 594 Dilution 1:500	Invitrogen	Cat. # A-11037, RRID:AB_2534095 Lot.1608397
Goat anti-Rabbit Secondary Antibody, Alexa Fluor 647 Dilution 1:500	Invitrogen	Cat. # A-21244, RRID:AB_2535812 Lot.42246A
Goat anti-Mouse IgG1 Secondary Antibody, Alexa Fluor 488 Dilution 1:500	Invitrogen	Cat. # A-21121, RRID:AB_2535764 Lot.2040297
Goat anti-Mouse IgG1 Secondary Antibody, Alexa Fluor 555 Dilution 1:500	Invitrogen	Cat. # A-21127, RRID:AB_141596 Lot.2465092
Goat anti-Mouse IgG1 Secondary Antibody, Alexa Fluor 647 Dilution 1:500	Invitrogen	Cat. # A-21240, RRID:AB_141658 Lot.2482960
Goat anti-Mouse IgG2a Secondary Antibody, Alexa Fluor 488 Dilution 1:500	Invitrogen	Cat. # A-21131, RRID:AB_141618 Lot.2581803
Goat anti-Mouse IgG2a Secondary Antibody, Alexa Fluor 555 Dilution 1:500	Invitrogen	Cat. # A-21137, RRID:AB_2535776 Lot.2697001
Goat anti-Mouse IgG2a Secondary Antibody, Alexa Fluor 647 Dilution 1:500	Invitrogen	Cat. # A-21241, RRID:AB_141698 Lot.2482961
Goat anti-Mouse IgG2b Secondary Antibody, Alexa Fluor 488 Dilution 1:500	Invitrogen	Cat. # A-21141, RRID:AB_141626 Lot.2306817
Goat anti-Mouse IgG2b Secondary Antibody, Alexa Fluor 555 Dilution 1:500	Invitrogen	Cat. # A-21147, RRID:AB_2535783 Lot.2438514
Donkey anti-Goat IgG (H+L) Secondary Antibody, Alexa Fluor 555 Dilution 1:500	Invitrogen	Cat. # A-21432, RRID:AB_141788 Lot.2026158
Sheep anti-Mouse IgG-HRP Dilution 1:2500	GE Healthcare	Cat. # NA931, RRID:AB_772210 Lot.364468
Mouse anti-Goat IgG-HRP Dilution 1:2500	Santa Cruz	Cat. # sc-2354, RRID:AB_628490 Lot.B0520

Sheep anti-rabbit IgG-HRP Dilution 1:2500	GE Healthcare	Cat. # NA934, RRID:AB_772206 Lot.364579
Plasmids	Source	Identifier
pXAT2	Oceguera-Yanez et al.,	Addgene: #80494
pAAVS1-CAG-EGFP	Oceguera-Yanez et al.,	Addgene: #80491
pgLAP5-CRYS-Arl13b-bPAC-EGFP	Truong et al.,	N/A
pAAVS1-CAG-EGFP-APEX2	This paper	N/A
pAAVS1-CAG-NPHP31-203-EGFP-APEX2	This paper	N/A
pAAVS1-CAG-bPAC-EGFP	This paper	N/A
pAAVS1-CAG-Arl13B-bPAC-EGFP	This paper	N/A
pAAVS1-CAG-bPACF198Y-EGFP	This paper	N/A
pAAVS1-CAG-Arl13B-bPACF198Y-EGFP	This paper	N/A
pAAVS1-CAG-GPR161-EGFP	This paper	N/A
pAAVS1-CAG-GPR161HHC-EGFP	This paper	N/A
pAAVS1-P-CAG-mCh	Oceguera-Yanez et al.,	Addgene: #80492
pEF5B-FRT-cilia-APEX	Mick et al.,	Addgene: #73186
pLJM-Empty	Golden et al.,	Addgene: #91980
pCAGGS-Ga15	Niwa et al.,	N/A
pCDMd1-GPR161	This paper	N/A
pCDMd1-GPR161HHC	This paper	N/A
pCDMd1-GPR161-EGFP	This paper	N/A
pCDMd1-GPR161HHC-EGFP	This paper	N/A
Reagents	Source	Identifier
TrypLE express enzyme	Gibco	Cat. # 12604021
StemFit® AK02N	Ajinomoto	Cat. # RCAK02N
Y-27632	Chemscene	Cat. # CS-0878
iMatrix-511 silk (recombinant laminin-511)	Takara Bio	Cat. # T311
Stem Cell Banker	Takara Bio	Cat. # CB045
Alt-R tracrRNA with Atto550	Integrated DNA Technologies	Cat. # 1075927
Alt-R S.p.HiFi Cas9 Nuclease V3	Integrated DNA Technologies	Cat. # 1081060
Electroporation Cuvette 0.2cm	Bio-Rad	Cat. # 1652086
DAPI	Sigma-Aldrich	Cat. # D8417
DMEM/F12	Invitrogen	Cat. # 11330-032
ATP	Sigma-Aldrich	Cat. # 10519979001
Magnesium Chloride Hexahydrate	Sigma-Aldrich	Cat. # 19-0190-5
Potassium phosphate monobasic	Sigma-Aldrich	Cat. # P9791
D-(+)-Glucose	Nacalai Tesque	Cat. # 16806
Tris(hydroxymethyl)aminomethane	Nacalai Tesque	Cat. # 35434-34
Potassium Chloride	Nacalai Tesque	Cat. # 28514-75
Nonidet P-40 (NP-40)	Nacalai Tesque	Cat. # 23640-94
Tween 20 (Polyoxyethylene Sorbitan Monolaurate)	Nacalai Tesque	Cat. # 28353-85
Proteinase K	Sigma-Aldrich	Cat. # 03 115 852 001
Q5 High-Fidelity DNA Polymerase	New England Biolabs	Cat. # M0491
96-well round bottom ultra-low attachment microplate	Corning	Cat. # 7007
Nunclon Sphera 96-Well, Nunclon Sphera-Treated, U-Shaped-Bottom Microplate	Thermo Fisher Scientific	Cat. # 174925
KnockOut Serum Replacement	Thermo Fisher Scientific	Cat. # 10828010
MEM Non-essential amino acids solution (100X)	Gibco	Cat. # 11140050
L-glutamine	Thermo Fisher Scientific	Cat. # 25030081
LDN-193189	Cayman Chemical	Cat. # 19396
SB-431542	Cayman Chemical	Cat. # 13031
XAV939	Cayman Chemical	Cat. # 13596
2-mercaptoethanol	FUJIFILM Wako Pure Chemical	Cat. # 133-14571
200 mM L-Glutamine	Nacalai Tesque	Cat. # 16948-04
Penicillin-Streptomycin Mixed Solution	Nacalai Tesque	Cat. # 26253-84
MACS Neuro Medium	Miltenyi Biotec	Cat. # 130-093-570
Insulin	Nacalai Tesque	Cat. # 12878-86
N-2 MAX Media Supplement	R&D Systems	Cat. # AR009
NeuroBrew-21 Supplement without VitA	Miltenyi Biotec	Cat. # 130-097-263
NeuroBrew-21 Supplement	Miltenyi Biotec	Cat. # 130-093-566
L-Ascorbic acid	Sigma-Aldrich	Cat. # A92902
2-hydroxyethyl methacrylate	Sigma-Aldrich	Cat. # P3932
EZ-sphere 24-well plates	Iwaki	Cat. # 4820-900SP
Disposable surgical scalpel	Kai medical	Cat. # No. 10 510-A
Matrigel	Corning	Cat. # 354234
EGF	Alomone Labs	Cat. # E-100
bFGF	Gift from Dr. Spees	Shimada et al., J. Neuroscience 2012
Poly-D-Lysine	R&D Systems	Cat. # 3439-100-01
Laminin	Corning	Cat. # 354232
Paraformaldehyde	FUJIFILM Wako Pure Chemical	Cat. # 162-16065
Tissue-Tek O.C.T. Compound	Sakura Finetek Japan	Cat. # 4583
Sucrose	Nacalai Tesque	Cat. # 09589-05
Immunosaver	Nisshin EM	Cat. # 333
Triton X-100	Sigma-Aldrich	Cat. # 30-5140-5
Fluoromount-G	SouthernBiotech	Cat. # 0100-01
Normal Serum Block (GOAT)	BioLegend	Cat. # 927503
CC/Mount	Diagnostic Biosystems	Cat. # K002
FBS	Sigma-Aldrich	Cat. # 172012
Plastic cover glass	Sumitomo Bakelite	Cat. # MS-92132Z

Marienfeld Superior Borosilicate Glass Cover Glasses, Round	Paul Marienfeld GmbH	Cat. # 0111520
Isogen II	NipponGene	Cat. # 311-07361
ReverTra Ace qPCR RT Master Mix with gDNA remover	Toyobo	Cat. # FSQ-301
Faststart universal sybr green master (ROX)	Sigma-Aldrich	Cat. # 4913914001
Halt Protease and Phosphatase Inhibitor Cocktail	Thermo Fisher Scientific	Cat. # 78445
Sodium Chloride	Sigma-Aldrich	Cat. # S3014
Sodium deoxycholate	Sigma-Aldrich	Cat. # D6750
SDS (Sodium dodecyl sulfate)	Sigma-Aldrich	Cat. # 28-3260-5
Glycerol	Sigma-Aldrich	Cat. # 12-1120-3
Bromophenolblue	Sigma-Aldrich	Cat. # 03-4140-3
DTT	Nacalai Tesque	Cat. # 14128-62
Polyvinylidene difluoride membrane	Millipore	Cat. # IPVH00010
Glycine	Sigma-Aldrich	Cat. # G7126
Methanol	Sigma-Aldrich	Cat. # 19-2410-4
Gel blotting paper	Whatman	Cat. # GB003
BLUE Star PLUS Prestained Protein-Ladder	Nippon Genetics	Cat. # NE-MWP04
Luminata Forte Western HRP Substrate	Millipore	Cat. # WBLUF0500
Bullet PAGE Plus Precast Gel 5-20% 13well	Nacalai Tesque	Cat. # 21793-64
Bullet Blocking One for Western Blotting	Nacalai Tesque	Cat. # 13779-01
Bullet ImmunoReaction Buffer	Nacalai Tesque	Cat. # 18439-85
SuperSignal West Atto Ultimate Sensitivity Chemiluminescent Substrate	Thermo Fisher Scientific	Cat. # A38556
Western Blot Stripping Buffer	Thermo Fisher Scientific	Cat. # 21059
76 razor	Nissin EM	Cat. # 4761
Glutaraldehyde Solution	Electron Microscopy Sciences	Cat. # 16220-P
Osmium tetroxide	MERCK	Cat. # 24505
Quetol 812 epoxy resin	Nisshin EM	Cat. # 340
Uranyl acetate	Merck	Cat. # 8473
Arduino Uno SMD R3	Arduino	Cat. # A000073
Gravity relay module	DFRobot	Cat. # DFR0643
470 nm LED device	Optocode	Cat. # LEDB-SBOXHP
660 nm LED stand light	Optocode	Cat. # LED660-100STND
iFluor555-streptavidin conjugate	AAT Bioquest	Cat. # 16959
Lipofectamine Stem Transfection Reagent	Thermo Fisher Scientific	Cat. # STEM00001
Biotinyl tyramide	Focus Biomolecules	Cat. # 10-3446
Trolox	Cayman	Cat. # 10011659
Hydrogen Peroxide	FUJIFILM Wako Pure Chemical	Cat. # 081-04215
Sodium Azide	FUJIFILM Wako Pure Chemical	Cat. # 194-01275
Sodium ascorbate	Nacalai Tesque	Cat. # 03422-32
Sodium hydrogencarbonate	Sigma-Aldrich	Cat. # 28-1850-5
Urea	Nacalai Tesque	Cat. # 35940-81
Bond-Breaker TCEP solution, neutral pH	Thermo Fisher Scientific	Cat. # 77720
Streptavidin magnetic beads	Pierce	Cat. # 88816
2-Chloroacetamide (CAM)	FUJIFILM Wako Pure Chemical	Cat. # 032-09762
HEPES	Sigma-Aldrich	Cat. # H4034
Calcium Chloride	FUJIFILM Wako Pure Chemical	Cat. # 039-00475
Trypsin	Pierce	Cat. # 90057
Trifluoroacetic acid	FUJIFILM Wako Pure Chemical	Cat. # 206-10731
Acetonitrile	Kanto Chemical	Cat. # 01033-23
SDB-RPS	CDS	Cat. # 2241
Ultrapure water	FUJIFILM Wako Pure Chemical	Cat. # 214-01301
Dynal MPC-S magnetic particle concentrator	Invitrogen	Cat. # 120.20D
Formic acid	FUJIFILM Wako Pure Chemical	Cat. # 067-04531
C18 column	Nikkyo Technos	Cat. # NTCC-360/75-3-125
Human brain marathon-ready cDNA library	Clontech	Cat. # 639300
PFN22A HaloTag CMVd1 Flexi vector	Promega	Cat. # G2841
DMEM	Nacalai Tesque	Cat. # 08458-16
FBS	Nichirei Biosciences	Cat. # 175012-500ML
Penicillin and Streptomycin	FUJIFILM Wako Pure Chemical	Cat. # 168-23191
ViaFect	Promega	Cat. # E4981
Lipofectamine 3000	Thermo Fisher Scientific	Cat. # L3000001
Copper(II) chloride	FUJIFILM Wako Pure Chemical	Cat. # 039-04152
Forskolin	Tokyo Chemical Industry	Cat. # F0855
Critical Commercial kit		
RNA Premium kit	Nippon Genetics	Cat. # FG-81050
In-Fusion HD cloning kit	Takara Bio	Cat. # 639650
KOD-Plus-Mutagenesis Kit	TOYOBO	Cat. # SMK-101
NEBNext Ultra II RNA library prep kit for Illumina	New England Biolabs	Cat. # E7770
Q5 Site-Directed Mutagenesis Kit	New England Biolabs	Cat. # E0554
NEBuilder HiFi DNA Assembly Master Mix	New England Biolabs	Cat. # E2621
Bright-Glo Luciferase assay	Promega	Cat. # E2620
Software and algorithms		
Fiji (ImageJ) (Version 1.54f)	RRID:SCR_002285	https://imagej.net/software/fiji/downloads
Olympus Fluoview (Version 2.6)	RRID:SCR_020339	https://www.olympus-lifescience.com/en/laser-scanning/fv3000/

Zeiss Zen (Version 3.5)	RRID:SCR_013672	https://www.zeiss.com/microscopy/us/products/software/zeiss-zen.html
Hitachi S-4800 (Version 5.3)	N/A	N/A
Jeol TEM Center (Version 1.7)	N/A	https://www.jeol.com/products/scientific/tem/JEM-1400Plus.php
Olympus Cell sense (Version 2.3)	RRID:SCR_014551	https://www.olympus-lifescience.com/en/software/cellsens/
Nikon NIS-Elements (Version 5.2)	RRID:SCR_014329	https://www.microscope.healthcare.nikon.com/products/software/nis-elements
GraphPad Prism (Version 9.5.1)	RRID:SCR_002798	https://www.graphpad.com/
BD FACSDiva (Version 9.4)	RRID:SCR_001456	https://www.bdbiosciences.com/en-us
FlowJo (Version 10.8.1)	RRID:SCR_008520	https://www.flowjo.com/
ApE (Version 3.1.3)	RRID:SCR_014266	https://jorgensen.biology.utah.edu/wayned/ape/
ChatGPT 5.2	RRID:SCR_023775	https://chat.openai.com/
DeepL Write	N/A (For the grammar check)	https://www.deepl.com/write
Grammarly	RRID:SCR_023778	https://www.grammarly.com/
Mendeley(v2.140.1)	RRID:SCR_002750	https://www.mendeley.com/search/
Arduino IDE 2.3.1	RRID:SCR_024884	https://www.arduino.cc/en/software
Fastp (v0.23.2)	RRID:SCR_016962	https://github.com/OpenGene/fastp
Bowtie2 (v2.2.5)	RRID:SCR_016368	https://bowtie-bio.sourceforge.net/bowtie2/index.shtml
SAMtools (v1.15.1)	RRID:SCR_002105	https://www.htslib.org/
deepTools (v3.5.1)	RRID:SCR_016366	https://deeptools.readthedocs.io/en/develop/
HOMER (v4.11)	RRID:SCR_010881	http://homer.ucsd.edu/homer/
IGV (v2.17.4)	RRID:SCR_011793	https://igv.org/doc/desktop/
GSEA 4.3.3	RRID:SCR_003199	https://www.gsea-msigdb.org/gsea/index.jsp
BioJupies	RRID:SCR_016346	https://maayanlab.cloud/biojupies/
DIA-NN 2.0.2 Academia	RRID:SCR_022865	https://aptila.bio/
PyMOL 3.1.4.1	RRID:SCR_000305	https://pymol.org/
g:Profiler (Version e112_eg59_p19_25aa4782)	RRID:SCR_006809	https://biit.cs.ut.ee/gprofiler/gost
Affinity Designer(v1.10.8)	RRID:SCR_016952	https://www.affinity.studio/
Experimental models: Cell lines		
human 201B7 iPS cells	Riken	201B7
human Windy iPS cells	Nishino et al.,	Windy (#51)
human 201B7 iPS cells ARL13B-KO1	This paper	N/A
human 201B7 iPS cells ARL13B-KO2	This paper	N/A
human 201B7 iPS cells GPR161-KO1	This paper	N/A
human 201B7 iPS cells GPR161-KO2	This paper	N/A
human Windy iPS cells ARL13B-KO1	This paper	N/A
human Windy iPS cells ARL13B-KO2	This paper	N/A
human Windy iPS cells GPR161-KO1	This paper	N/A
human Windy iPS cells GPR161-KO2	This paper	N/A
cyto-EGFP-APEX2 201B7 iPS cells	This paper	N/A
cilia-EGFP-APEX2 201B7 iPS cells	This paper	N/A
cyto-bPAC-EGFP 201B7 GPR161 KO2 iPS cells	This paper	N/A
cilia-bPAC-EGFP 201B7 GPR161 KO2 iPS cells	This paper	N/A
cyto-bPACF198Y-EGFP 201B7 GPR161 KO2 iPS cells	This paper	N/A
cilia-bPACF198Y-EGFP 201B7 GPR161 KO2 iPS cells	This paper	N/A
GPR161-EGFP 201B7 GPR161 KO2 iPS cells	This paper	N/A
GPR161HHC-EGFP 201B7 GPR161 KO2 iPS cells	This paper	N/A
HEK293 cells	ATCC	CRL-1573
