# Supplementary material for: Light- and chemical-induced ciliary signaling governs dorsal/ventral regionalization of human telencephalic organoids
Source: Nat Commun. 2026 May 22;17:6712. doi: 10.1038/s41467-026-73505-2 (PMC13385379; doi:10.1038/s41467-026-73505-2)
Supplement: Supplementary file 2 — Description of Additional Supplementary Files [file 41467_2026_73505_MOESM2_ESM.pdf]

Description of Additional Supplementary Files

Title: Supplementary Data 1

Description: Candidate ciliary proteins identified by proximity labeling followed by unbiased mass spectrometry-based proteomic analysis of human telencephalic organoids derived from *CAG-NPHP3*$_{1\text{-}203}$*-EGFP-APEX2*-expressing 201B7 iPS cells (Cilia-APEX2 identified proteins) and *CAG-EGFP-APEX2*-expressing 201B7 iPS cells (Cyto-APEX2 identified proteins).

Title: Supplementary Data 2

Description: Differential gene expression analysis by RNA-seq of control and *ARL13B* KO human telencephalic organoids derived from Windy iPS cells and *ARL13B* KO2 Windy iPS cells, respectively.
