# Supplementary material for: Light- and chemical-induced ciliary signaling governs dorsal/ventral regionalization of human telencephalic organoids
Source: Nat Commun. 2026 May 22;17:6712. doi: 10.1038/s41467-026-73505-2 (PMC13385379; doi:10.1038/s41467-026-73505-2)
Supplement: Supplementary file 5 — Reporting Summary [file 41467_2026_73505_MOESM5_ESM.pdf]

nature portfolio

Corresponding author(s): Issei S. Shimada and Yoichi Kato

Last updated by author(s): 2026.4.22

Reporting Summary

Nature Portfolio wishes to improve the reproducibility of the work that we publish. This form provides structure for consistency and transparency in reporting. For further information on Nature Portfolio policies, see our Editorial Policies and the Editorial Policy Checklist.

Statistics

For all statistical analyses, confirm that the following items are present in the figure legend, table legend, main text, or Methods section.

n/a	Confirmed	
☐	☒	The exact sample size (*n*) for each experimental group/condition, given as a discrete number and unit of measurement
☐	☒	A statement on whether measurements were taken from distinct samples or whether the same sample was measured repeatedly
☐	☒	The statistical test(s) used AND whether they are one- or two-sided<br>*Only common tests should be described solely by name; describe more complex techniques in the Methods section.*
☒	☐	A description of all covariates tested
☐	☒	A description of any assumptions or corrections, such as tests of normality and adjustment for multiple comparisons
☐	☒	A full description of the statistical parameters including central tendency (e.g. means) or other basic estimates (e.g. regression coefficient) AND variation (e.g. standard deviation) or associated estimates of uncertainty (e.g. confidence intervals)
☐	☒	For null hypothesis testing, the test statistic (e.g. *F*, *t*, *r*) with confidence intervals, effect sizes, degrees of freedom and *P* value noted<br>*Give P values as exact values whenever suitable.*
☒	☐	For Bayesian analysis, information on the choice of priors and Markov chain Monte Carlo settings
☒	☐	For hierarchical and complex designs, identification of the appropriate level for tests and full reporting of outcomes
☒	☐	Estimates of effect sizes (e.g. Cohen's *d*, Pearson's *r*), indicating how they were calculated

Our web collection on statistics for biologists contains articles on many of the points above.

Software and code

Policy information about availability of computer code

Data collection	A detailed description of data collection is provided in the methods.Code for the Arduino uno gravity relay module for optogenetics is available https://zenodo.org/records/19492078.
Data analysis	A detailed description of data collection is provided in the methods. The code for optogenetics is shown in Supplementary Figure 10. Data analysis software are as follows:<br>Fiji (ImageJ) (Version 1.54f)<br>Olympus Fluoview (Version 2.6)<br>Zeiss Zen (Version 3.5)<br>Hitachi S-4800 (Version 5.3)<br>Jeol TEM Center (Version 1.7)<br>Olympus Cell sense (Version 2.3)<br>Nikon NIS-Elements (Version 5.2)<br>GraphPad Prism (Version 9.5.1)<br>BD FACSDiva (Version 9.4)<br>FlowJo (Version 10.8.1)<br>ApE (Version 3.1.3)<br>ChatGPT 5.4<br>DeepL Write<br>Grammarly<br>Mendeley (v2.140.1)

Arduino IDE 2.3.1
Fastp (v0.23.2)
Bowtie2 (v2.2.5)
SAMtools (v1.15.1)
deepTools (v3.5.1)
HOMER (v4.11)
IGV (v2.17.4)
GSEA 4.3.3
BioJupies
DIA-NN 2.0.2 Academia
PyMOL 3.1.4.1
g:Profiler (Version e112_eg59_p19_25aa4782)
Affinity Designer (v1.10.8)

For manuscripts utilizing custom algorithms or software that are central to the research but not yet described in published literature, software must be made available to editors and reviewers. We strongly encourage code deposition in a community repository (e.g. GitHub). See the Nature Portfolio guidelines for submitting code & software for further information.

Data

Policy information about availability of data

All manuscripts must include a data availability statement. This statement should provide the following information, where applicable:

- Accession codes, unique identifiers, or web links for publicly available datasets
- A description of any restrictions on data availability
- For clinical datasets or third party data, please ensure that the statement adheres to our policy

RNA sequencing data have been deposited in the DNA Data Bank of Japan (DDBJ) BioProject (PRJDB20782) https://ddbj.nig.ac.jp/search/entry/bioproject/PRJDB20782. Mass spectrometry analysis data have been deposited in the Japan Proteome Standard Repository (JPST003807) https://repository.jpostdb.org/entry/JPST003807.

Research involving human participants, their data, or biological material

Policy information about studies with human participants or human data. See also policy information about sex, gender (identity/presentation), and sexual orientation and race, ethnicity and racism.

Reporting on sex and gender	Previously characterized human induced pluripotent stem cells (iPS cells; 201B7 (RIKEN; female; https://cellbank.brc.riken.jp/cell_bank/CellInfo/?cellNo=HPS0063&lang=en) and #51 (also known as Windy; Dr. Akihiro Umezawa at National Center for Child Health and Development Research Institute); derivative of MRC-5 cells, male; https://onlinelibrary.wiley.com/doi/10.1111/j.1365-2443.2010.01459.x) were used in the current study. Human fetal brain sections (15 gestational weeks, sex unknown) were kindly provided by National Center of Neurology and Psychiatry in Japan.
Reporting on race, ethnicity, or other socially relevant groupings	Race, ethnicity or other social relevant groupings were not used in study design or analysis.
Population characteristics	201B7 iPS cells: Caucasian, 37 years old and Female. Windy iPS cells : Derivative of MRC-5 (Fetal lung origin) cells, Male. Human fetus brain section: 15 gestational weeks. Sex is unknown.Race, ethnicity or other social relevant groupings were not used in study design or analysis.
Recruitment	Fetal human brain sections were previously collected at National Center of Neurology and Psychiatry in Japan with parental consent.
Ethics oversight	Fetal human brain sections were previously generated at National Center of Neurology and Psychiatry in Japan with parental consent. The sections were de-identified and anonymized. The research plan was approved by Nagoya City University (IRB #: 60-24-0081).

Note that full information on the approval of the study protocol must also be provided in the manuscript.

Field-specific reporting

Please select the one below that is the best fit for your research. If you are not sure, read the appropriate sections before making your selection.

[x] Life sciences   [ ] Behavioural & social sciences   [ ] Ecological, evolutionary & environmental sciences

For a reference copy of the document with all sections, see nature.com/documents/nr-reporting-summary-flat.pdf

Life sciences study design

All studies must disclose on these points even when the disclosure is negative.

Sample size	Sample sizes are reported in each figure legend and Source Data. Sample sizes were based on our experience with these assays. No statistical method was used to predetermine sample size.

Data exclusions	Organoids failed to grow were excluded from the experiments and analysis.
Replication	The number of replication is reported in each figure legend and Source Data. Organoids were produced from two iPS cell lines.
Randomization	No randomization was performed.
Blinding	No blinding was performed. Blinding was not feasible due to the large number of samples and the experimental workflow.

Reporting for specific materials, systems and methods

We require information from authors about some types of materials, experimental systems and methods used in many studies. Here, indicate whether each material, system or method listed is relevant to your study. If you are not sure if a list item applies to your research, read the appropriate section before selecting a response.

Materials & experimental systems

n/a	Involved in the study
☐	☒ Antibodies
☐	☒ Eukaryotic cell lines
☒	☐ Palaeontology and archaeology
☒	☐ Animals and other organisms
☒	☐ Clinical data
☒	☐ Dual use research of concern
☒	☐ Plants

Methods

n/a	Involved in the study
☒	☐ ChIP-seq
☒	☐ Flow cytometry
☒	☐ MRI-based neuroimaging

Antibodies

Antibodies used

Acetylated alpha-tubulin (6-11B-1) Dilution 1:500 Sigma-Aldrich Cat. # T6793, RRID:AB_477585
ARL13B Dilution 1:500 Proteintech Cat. # 17711-1-AP, RRID:AB_2060867
ARL13B Dilution 1:500 BioLegend Cat. # 857602, RRID:AB_2801216
Beta-actin (AC-15) Dilution 1:2500 Santa Cruz Cat. # sc-69879, RRID:AB_1119529
Beta-catenin Dilution 1:500 Sigma-Aldrich Cat. # C2206, RRID:AB_476831
Beta-tubulin III (TUBB3) Dilution 1:500 Santa Cruz Cat. # sc-80005, RRID:AB_2210816
BLBP Dilution 1:500 Millipore Cat. # ABN14, RRID:AB_10000325
FOXA2 (HNF-3beta; RY-7) Dilution 1:500 Santa Cruz Cat. # sc-101060, RRID:AB_1124660
FOXG1 Dilution 1:500 Abcam Cat. # ab18259, RRID:AB_732415
Gamma-tubulin (C-11) Dilution 1:500 Santa Cruz Cat. # sc-17787, RRID:AB_628417
Gamma-tubulin (TU-30) Dilution 1:500 Santa Cruz Cat. # sc-51715, RRID:AB_630410
GAPDH Dilution 1:2500 Novus Cat. # NB300-322, RRID:AB_10001458
GFP (B-2) Dilution 1:500 Santa Cruz Cat. # sc-51715, RRID:AB_630410
GLI2 Dilution 1:500 R&D Systems Cat. # AF3526, RRID:AB_2279108
GLI3 Dilution 1:2500 R&D Systems Cat. # AF3690, RRID:AB_2232499
GPR161 Dilution 1:500 Sigma-Aldrich Cat. # AV42354, RRID:AB_1849281
GPR161 Dilution 1:500 Proteintech Cat. # 13398-1-AP, RRID:AB_2113965
GSX2 Dilution 1:500 Sigma-Aldrich Cat. # ABN162, RRID:AB_11203296
IFT88 Dilution 1:500 Proteintech Cat. # 13967-1-AP, RRID:AB_2121979
NKX2.1 (TTF-1; 8G7G3/1) Dilution 1:500 Diagnostic BioSystems Cat. # MOB285-01, RRID:AB_3730160
NKX2.2 Dilution 1:20 Hybridoma Bank Cat. # 74.5A5, RRID:AB_531794
Oct3/4 (C-10) Dilution 1:500 Santa Cruz Cat. # sc-5279, RRID:AB_628051
Olig2 Dilution 1:500 R&D Systems Cat. # AF2418, RRID:AB_2157554
PAX6 Dilution 1:500 BioLegend Cat. # 901301, RRID:AB_2565003
PAX7 Dilution 1:20 Hybridoma Bank Cat. # Pax7, RRID:AB_2299243
p-Histone H3 (C-2) Dilution 1:1000 Santa Cruz Cat. # sc-374669, RRID:AB_11150094
Poly-E (GT335) Dilution 1:1000 Adipogen Cat. # AG-20B-0020, RRID:AB_2335608
SMO (E-5) Dilution 1:500 Santa Cruz Cat. # sc-166685, RRID:AB_2239686
SOX2 (E-4) Dilution 1:500 Santa Cruz Cat. # sc-365823, RRID:AB_10842165
Goat anti-Rabbit Secondary Antibody, Alexa Fluor 488 Dilution 1:500 Invitrogen Cat. # A-11008, RRID:AB_143165
Goat anti-Rabbit Secondary Antibody, Alexa Fluor 594 Dilution 1:500 Invitrogen Cat. # A-11037, RRID:AB_2534095
Goat anti-Rabbit Secondary Antibody, Alexa Fluor 647 Dilution 1:500 Invitrogen Cat. # A-21244, RRID:AB_2535812
Goat anti-Mouse IgG1 Secondary Antibody, Alexa Fluor 488 Dilution 1:500 Invitrogen Cat. # A-21121, RRID:AB_2535764
Goat anti-Mouse IgG1 Secondary Antibody, Alexa Fluor 555 Dilution 1:500 Invitrogen Cat. # A-21127, RRID:AB_141596
Goat anti-Mouse IgG1 Secondary Antibody, Alexa Fluor 647 Dilution 1:500 Invitrogen Cat. # A-21240, RRID:AB_141658
Goat anti-Mouse IgG2a Secondary Antibody, Alexa Fluor 488 Dilution 1:500 Invitrogen Cat. # A-21131, RRID:AB_141618
Goat anti-Mouse IgG2a Secondary Antibody, Alexa Fluor 555 Dilution 1:500 Invitrogen Cat. # A-21137, RRID:AB_2535776
Goat anti-Mouse IgG2a Secondary Antibody, Alexa Fluor 647 Dilution 1:500 Invitrogen Cat. # A-21241, RRID:AB_141698

Goat anti-Mouse IgG2b Secondary Antibody, Alexa Fluor 488 Dilution 1:500 Invitrogen Cat. # A-21141, RRID:AB_141626
Goat anti-Mouse IgG2b Secondary Antibody, Alexa Fluor 555 Dilution 1:500 Invitrogen Cat. # A-21147, RRID:AB_2535783
Donkey anti-Goat IgG (H+L) Secondary Antibody, Alexa Fluor 555 Dilution 1:500 Invitrogen Cat. # A-21432, RRID:AB_141788
Sheep anti-Mouse IgG-HRP Dilution 1:2500 GE Healthcare Cat. # NA931, RRID:AB_772210
Mouse anti-Goat IgG-HRP Dilution 1:2500 Santa Cruz Cat. # sc-2354, RRID:AB_628490
Sheep anti-rabbit IgG-HRP Dilution 1:2500 GE Healthcare Cat. # NA934, RRID:AB_772206

Validation

All antibodies used in this study are commercially available.
Acetylated alpha-tubulin (6-11B-1) https://www.sigmaaldrich.com/US/en/product/sigma/t6793
ARL13B https://www.ptglab.com/products/ARL13B-Antibody-17711-1-AP.htm
ARL13B https://www.biolegend.com/en-us/products/purified-anti-arl13b-antibody-16812
Beta-actin (AC-15) https://www.scbt.com/p/beta-actin-antibody-ac-15
Beta-catenin https://www.sigmaaldrich.com/US/en/product/sigma/c2206
Beta-tubulin III (TUBB3) https://www.scbt.com/p/beta3-tubulin-antibody-2g10
BLBP https://www.sigmaaldrich.com/US/en/product/mm/abn14
FOXA2 (HNF-3beta; RY-7) https://www.scbt.com/p/hnf-3beta-antibody-ry-7
FOXG1 https://www.abcam.com/en-us/products/primary-antibodies/foxg1-antibody-ab18259
Gamma-tubulin (C-11) https://www.scbt.com/p/gamma-tubulin-antibody-c-11
Gamma-tubulin (TU-30) https://www.scbt.com/p/gamma-tubulin-antibody-tu-30
GAPDH https://www.novusbio.com/products/gapdh-antibody_nb300-322
GFP (B-2) https://www.scbt.com/p/gfp-antibody-b-2
GLI2 https://www.rndsystems.com/products/human-gli-2-antibody_af3526
GLI3 https://www.rndsystems.com/products/human-mouse-gli-3-antibody_af3690
GPR161 https://www.sigmaaldrich.com/US/en/product/sigma/av42354
GPR161 https://www.ptglab.com/products/GPR161-Antibody-13398-1-AP.htm
GSX2 https://www.sigmaaldrich.com/US/en/product/mm/abn162
IFT88 https://www.ptglab.com/products/IFT88-Antibody-13967-1-AP.htm
NKX2.1 (TTF-1; 8G7G3/1) https://dbiosys.com/product/ttf-1-thyroid-transcription-factor-1/
NKX2.2 https://dshb.biology.uiowa.edu/74-5A5
Oct3/4 (C-10) https://www.scbt.com/p/oct-3-4-antibody-c-10
Olig2 https://www.rndsystems.com/products/human-mouse-rat-olig2-antibody_af2418
PAX6 https://www.biolegend.com/en-us/products/purified-anti-pax-6-antibody-11511
PAX7 https://dshb.biology.uiowa.edu/PAX7
p-Histone H3 (C-2) https://www.scbt.com/p/p-histone-h3-antibody-c-2
Poly-E (GT335) https://adipogen.com/ag-20b-0020-anti-polyglutamylation-modification-mab-gt335.html
SMO (E-5) https://www.scbt.com/scbt/product/smo-antibody-e-5
SOX2 (E-4) https://www.scbt.com/p/sox-2-antibody-e-4
Goat anti-Rabbit Secondary Antibody, Alexa Fluor 488 https://www.thermofisher.com/antibody/product/Goat-anti-Rabbit-IgG-H-L-Cross-Adsorbed-Secondary-Antibody-Polyclonal/A-11008
Goat anti-Rabbit Secondary Antibody, Alexa Fluor 594 https://www.thermofisher.com/antibody/product/Goat-anti-Rabbit-IgG-H-L-Highly-Cross-Adsorbed-Secondary-Antibody-Polyclonal/A-11037
Goat anti-Rabbit Secondary Antibody, Alexa Fluor 647 https://www.thermofisher.com/antibody/product/Goat-anti-Rabbit-IgG-H-L-Cross-Adsorbed-Secondary-Antibody-Polyclonal/A-21244
Goat anti-Mouse IgG1 Secondary Antibody, Alexa Fluor 488 https://www.thermofisher.com/antibody/product/Goat-anti-Mouse-IgG1-Cross-Adsorbed-Secondary-Antibody-Polyclonal/A-21121
Goat anti-Mouse IgG1 Secondary Antibody, Alexa Fluor 555 https://www.thermofisher.com/antibody/product/Goat-anti-Mouse-IgG1-Cross-Adsorbed-Secondary-Antibody-Polyclonal/A-21127
Goat anti-Mouse IgG1 Secondary Antibody, Alexa Fluor 647 https://www.thermofisher.com/antibody/product/Goat-anti-Mouse-IgG1-Cross-Adsorbed-Secondary-Antibody-Polyclonal/A-21240
Goat anti-Mouse IgG2a Secondary Antibody, Alexa Fluor 488 https://www.thermofisher.com/antibody/product/Goat-anti-Mouse-IgG2a-Cross-Adsorbed-Secondary-Antibody-Polyclonal/A-21131
Goat anti-Mouse IgG2a Secondary Antibody, Alexa Fluor 555 https://www.thermofisher.com/antibody/product/Goat-anti-Mouse-IgG2a-Cross-Adsorbed-Secondary-Antibody-Polyclonal/A-21137
Goat anti-Mouse IgG2a Secondary Antibody, Alexa Fluor 647 https://www.thermofisher.com/antibody/product/Goat-anti-Mouse-IgG2a-Cross-Adsorbed-Secondary-Antibody-Polyclonal/A-21241
Goat anti-Mouse IgG2b Secondary Antibody, Alexa Fluor 488 https://www.thermofisher.com/antibody/product/Goat-anti-Mouse-IgG2b-Cross-Adsorbed-Secondary-Antibody-Polyclonal/A-21141
Goat anti-Mouse IgG2b Secondary Antibody, Alexa Fluor 555 https://www.thermofisher.com/antibody/product/Goat-anti-Mouse-IgG2b-Cross-Adsorbed-Secondary-Antibody-Polyclonal/A-21147
Donkey anti-Goat IgG (H+L) Secondary Antibody, Alexa Fluor 555 https://www.thermofisher.com/antibody/product/Donkey-anti-Goat-IgG-H-L-Cross-Adsorbed-Secondary-Antibody-Polyclonal/A-21432
Sheep anti-Mouse IgG-HRP https://www.cytivalifesciences.com/en/us/products/items/amersham-ecl-hrp-conjugated-antibodies-p-06260?selectedProduct=25005173
Mouse anti-Goat IgG-HRP https://www.scbt.com/p/mouse-anti-goat-igg-hrp
Sheep anti-rabbit IgG-HRP https://www.cytivalifesciences.com/en/us/products/items/amersham-ecl-hrp-conjugated-antibodies-p-06260?selectedProduct=25005174

Eukaryotic cell lines

Policy information about cell lines and Sex and Gender in Research

Cell line source(s)	Previously characterized human induced pluripotent stem cells (iPS cells; 201B7 (RIKEN; female; https://cellbank.brc.riken.jp/cell_bank/CellInfo/?cellNo=HPS0063&lang=en) and #51 (also known as Windy; Dr. Akihiro Umezawa at National Center for Child Health and Development Research Institute); derivative of MRC-5 cells, male; https://onlinelibrary.wiley.com/doi/10.1111/j.1365-2443.2010.01459.x). HEK293 cell line was purchased from ATCC (CRL-1573).
Authentication	iPS cells were directly obtained from RIKEN and Dr. Umezawa at National Center for Child Health and Development Research Institute. HEK293 cell line was purchased from ATCC. iPS cell lines were tested for the expression of pluripotency markers using immunostainings.
Mycoplasma contamination	The iPS cell lines were tested for mycloplasma contamination using a PCR-based test and were found to be negative for mycoplasma.
Commonly misidentified lines (See ICLAC register)	None.

Plants

Seed stocks	*Report on the source of all seed stocks or other plant material used. If applicable, state the seed stock centre and catalogue number. If plant specimens were collected from the field, describe the collection location, date and sampling procedures.*
Novel plant genotypes	*Describe the methods by which all novel plant genotypes were produced. This includes those generated by transgenic approaches, gene editing, chemical/radiation-based mutagenesis and hybridization. For transgenic lines, describe the transformation method, the number of independent lines analyzed and the generation upon which experiments were performed. For gene-edited lines, describe the editor used, the endogenous sequence targeted for editing, the targeting guide RNA sequence (if applicable) and how the editor was applied.*
Authentication	*Describe any authentication procedures for each seed stock used or novel genotype generated. Describe any experiments used to assess the effect of a mutation and, where applicable, how potential secondary effects (e.g. second site T-DNA insertions, mosiacism, off-target gene editing) were examined.*
