# Supplementary material for: Light- and chemical-induced ciliary signaling governs dorsal/ventral regionalization of human telencephalic organoids
Source: Nat Commun. 2026 May 22;17:6712. doi: 10.1038/s41467-026-73505-2 (PMC13385379; doi:10.1038/s41467-026-73505-2)
Supplement: Supplementary file 6 — Transparent Peer Review file [file 41467_2026_73505_MOESM6_ESM.pdf]

nature portfolio

Peer Review File

Light- and chemical-induced ciliary signaling govern dorsal/ventral regionalization of human telencephalic organoids

Corresponding Author: Dr Issei Shimada

This file contains all reviewer reports in order by version, followed by all author rebuttals in order by version.

Version 0:

Reviewer comments:

Reviewer #1

(Remarks to the Author)
In their manuscript, Shimada et al analysed the role of primary cilia in human corticogenesis using cortical organoids. Starting with a proteomic profiling approach, they identified proteins located in primary cilia of cortical radial glial cells. Based on an enrichment of GTPase proteins in the ciliary proteome, they further investigated the role of ARL13B and GPR161. In contrast to inactivating ARL13B which caused a partial ventralisation of cortical organoids, the GPR161 mutation severely ventralized organoids. This ventralisation was rescued by a treatment with forskolin that activates adenylate cyclase and increased cAMP levels or by activating adenylate cyclase specifically in cilia. Finally, they show that removal of GPR161 from cilia is sufficient to induce a partial ventralisation of cortical organoids.

The experiments presented in this manuscript are technically challenging and well suited to address an important question in human corticogenesis, namely the role of primary cilia in forebrain regionalisation. However, there are several shortcomings in the interpretation of data and in the integration of their findings into the current literature.

Major points

1) The authors use the terms brain organoids and cortical organoids interchangeably but these terms denote different types of organoids. Brain organoids comprise several brain regions whereas cortical organoids are models of the cerebral cortex only. This misunderstanding is confusing for the reader and might have also affected the interpretation of ectopic marker expression. For example, NKX2.2, PAX7 and FOXA2 are not expressed in the cortex but in the spinal cord and midbrain. Hence, the ectopic expression of these markers might indicate that mutant organoids are not only ventralised but also caudalised. To test for this possibility, the authors should stain their organoids for FOXG1, a telencephalon marker, and NKX2.1 and GSX2 as ventral telencephalic markers. Their ARL13B bulk mRNA-seq experiment might also show an up-regulation of caudal brain and neural tube markers. The confusing use of the terms brain and cortical organoids might also have affected the interpretation of previous publications. For example, Schembs et al, 2022 used dual Smad inhibition to generate cortical, not brain organoids as stated on page 26. Moreover, this paper extensively analysed necessary and sufficient roles of SHH signalling in dorsal/ventral regionalisation of cortical organoids. The authors should change their interpretation correspondingly.

2) The authors should clearly acknowledge previous work from other labs and their contribution to deciphering the role of cilia in human forebrain regionalisation starting in the introduction to clearly indicate which novel questions their manuscript addresses.

3) In several immunofluorescence stainings of ciliary markers, it is very difficult or impossible to distinguish individual cilia, some images even appear blurry and out of focus (Figures 1K, 2C, 4F, 6G). The authors should present high resolution pictures. Moreover, the authors mainly show single rosettes but not whole organoids to illustrate the expression of dorsal/ventral markers. It remains unclear whether these single rosettes are representative of the expression of these markers in organoids as a whole.

4) The GLI3 western blots are of poor quality and show multiple unspecific bands. It remains unclear how the 88kDa GLI3

repressor form was identified. Moreover, a 180kDa band for the GLI3 full-length protein was not discernible. Finally, the blots were not quantified and the GLI3 repressor to full length protein ratio which is important for neural tube patterning was not determined.

5) The rescue experiments using the cyto-bPACF198Y-GPR161 KO and ciliary-bPACF198Y-GPR161 KO constructs, presented in Figure 5K and J, lack wildtype and GPR161 KO controls without the bPAC constructs. These controls were, however, included in the experiment using the leaky cyto-bPAC-GPR161 KO and ciliary-bPAC-GPR161 KO constructs (Supplementary Figure 6). Moreover, the authors claim that “the degree of reduction in the number of ventral neural stem cells between cytoplasmic and ciliary bPACF198Y activation before and after the light exposure was similar“, but they do not provide a quantification of these ratios. Using the averages presented in Figure 5K, it looks like the cytoplasmic construct has a much stronger effect than the ciliary construct.

6) Figure 6: The low GFP intensity of the GPR161HHC-EGFP construct in cilia without CuCl2 treatment presented in Figure 6F shows that much lower amounts of this fusion protein enter the cilium than the control protein suggesting a defect in ciliary location even before CuCl2 treatment. This is critical for analysing whether ciliary location of GPR161 is required to maintain a cortical fate. On the other hand, the GFP signal in the immunostainings (Figure 6G) appears much higher. There even are plenty of GFP+ but ARL13B- cilia. The authors should also comment why the GPR161-EGFP and GPR161HHC-EGFP fusion proteins restore PAX6 expression irrespective of their ciliary location which contradicts their conclusion.

7) Throughout the manuscript, the authors appear to have used the number of individual rosettes as n in statistical analyses, but do not state from how many different organoids rosettes were taken. This approach risks pseudo-replication as rosettes within the same organoid are certainly not independent from each other. Instead, the authors should calculate the average from several rosettes within one organoid and repeat this for several organoids. Use the number of organoids as their n number in statistical analyses.

Minor points

1) For the reviewers, it would be great to have access to the proteomic data set, i.e. a list of proteins located at the cilium, to judge the quality of the data.

2) page 11: Schembs already described cilia in human foetal cortex. Please, cite this paper.

3) page 11: Based on the sequencing data, the KO1 and KO2 lines in Fig. 2A appear to be different from the KO1 and KO2 lines in Supplementary Figure 2A. Could the authors please clarify this?

4) page 14: Schembs et al already describe GPR161 expression in RGC cilia. Please, cite this paper,

5) page 15: Spina bifida patients cannot indicate the importance of a gene in brain development, as the disorder affects the spinal cord not the brain.

6) page 24: The authors claim that “this rescue was partly mediated by GLI3 repressor formation” but they do not perform experiments to test the role of reduced GLI3 repressor levels. The GLI3 western blot only allows to correlate GLI3 repressor levels with the ventralisation phenotype.

Reviewer #2

(Remarks to the Author)
The study by Shimada et al. demonstrates that primary cilia act as precise signaling hubs during early brain patterning, where the ARL13B–GPR161–cAMP axis finely regulates dorsal versus ventral fate specification. Furthermore, it highlights the potential of spatially targeted interventions, such as optogenetics and chemogenetics, to modulate human neural stem cell lineage commitment.
The role of GPR161 as a key negative regulator of the SHH pathway has been well established in several studies. GPR161 functions as an essential “gatekeeper” of SHH signaling by localizing to the cilium, driving local cAMP production, and anchoring PKA to ensure that Gli transcription factors are processed into their repressor forms. Loss of GPR161 function removes this regulatory brake, leading to unchecked Gli activator formation and resulting in widespread ventralization or tissue overgrowth during development.
The authors present an elegant study utilizing a CRISPR-based GPR161 knockout model, which resulted in the elimination of dorsal ($PAX6^+$) progenitors and the induction of ventral markers (NKX2.2, FOXA2), thereby confirming the critical role of GPR161 in SHH pathway regulation and dorsal–ventral fate specification. Through optogenetic and chemogenetic approaches, they further demonstrate the importance of ciliary cAMP levels and the dynamic localization of GPR161 in determining neural stem cell fate.
One of the findings in this study appears to contradict the current understanding of SHH pathway regulation in primary cilia. The authors report that ARL13B knockout causes abnormal ciliary morphology, mislocalization of GPR161, and enhanced SHH signaling, leading to partial ventralization of neural stem cells even under dorsal-patterning conditions. Previous studies, however, suggest that loss of ARL13B does not hyperactivate SHH signaling in human cells. Instead, ARL13B is considered essential downstream of activated SMO for generating full Gli activator (GliA) output. In human medulloblastoma

cell lines, ARL13B knockdown reduces GLI1 and PTCH1 transcription in response to SHH ligand, SMO agonists, or activating SMO mutations. While the authors attribute the observed ventralization to reduced SHH repression via mislocalized GPR161 and decreased GliR, the status of GliA is not addressed. Considering that SHH pathway activation depends on the GliA/GliR balance, evaluating GliA levels would strengthen the mechanistic explanation of their findings. A luciferase-based SHH activity assay would further support their conclusions.
Minor issues: The resolution of Figures 2C and 4F is very low, making it difficult to discern ciliary structures. Given that Figures 1P and 4C demonstrate the authors' ability to produce high-resolution ciliary images, improving the resolution of Figures 2C and 4F is strongly recommended.
Overall, this study provides a compelling demonstration of the primary cilia–GPR161–cAMP axis in regulating dorsal versus ventral fate commitment. Addressing the issues outlined above would further strengthen the manuscript and make it a valuable contribution worthy of publication in Nature Communications.

Version 1:

Reviewer comments:

Reviewer #1

(Remarks to the Author)
The authors addressed most of my concerns and have certainly improved the quality of the manuscript, but there are still severe short comings with respect to the ventralisation of the organoids. On the positive side, the authors present new data on the expression of the ventral telencephalic markers NKX2.1 and GSX2 for ARL13B mutant organoids in the revised manuscript, however, the expression of these markers was not investigated in either the GPR161 mutant organoids or in the two rescue experiments. Their central claim that cilia mediated signalling is required to inhibit ventralisation under these conditions just rests on the ectopic expression of NKX2.2. In the mouse, this gene is not expressed in the ventral telencephalon and, to my knowledge, its expression in the human foetal telencephalon has not been described. In fact, their own transcriptomic data showed hardly any NKX2.2 expression (1 transcript on average per organoid) in control telencephalic organoids whereas the well-established ventral telencephalic markers GSX2 and NKX2.1 have 663 and 997 transcripts on average, respectively. Hence, to support their key claim, the authors must show that NKX2.2 is indeed expressed in human foetal ventral telencephalon using immunostainings or cite relevant studies. If NKX2.2 is not expressed in this tissue, they need to analyse NKX2.1 and/or GSX2 expression in GPR161 mutant organoids and in their rescue experiments instead as they have done for ARL13B mutant organoids. Otherwise, they can only assert that NKX2.2 ectopic expression was rescued. Claiming a ventralisation of the telencephalic organoids and its rescue would be an unjustified overstatement.

Minor points:
1) p13: FOXG1 is expressed in the telencephalon but not in the diencephalon. Hence, it is a telencephalic marker, not a forebrain marker. This may need to be amended throughout the manuscript.

2) Figures 5D and E: the graphs have been swapped in the revised manuscript compared to the original. The authors need to check this swap.

3)Figure 5I: Enlarge the organoid image that they cover the whole space.

Reviewer #2

(Remarks to the Author)
For the 2 key questions I raised
1. The authors' data suggested that ARL13B knockout causes enhanced SHH signaling. How would they account for the contradiction to previous studies suggesting ARL13B knockdown reduces SHH pathway responses?

They couldn't provide an explanation for that but in the manuscript, they cite prior work saying ARL13B deletion causes GPR161 mislocalization and impairs responsiveness to SHH signaling in primary cilia, therefore acknowledging literature where ARL13B loss is also linked to reduced/altered SHH responsiveness. The combination of their data and previous finding suggested that ARL13B mechanism is context-dependent in ciliary signaling.

2. The GLI-activator/repressor balance.

The authors pointed out technical difficulties that ARL13B KO primary cilia could not be reliably stained with commonly used ciliary antibodies, and also, transient gene expression is technically challenging in 3D telencephalic organoids, they were unable to perform a GLI binding domain luciferase assay to evaluate the level of GLI activation in their organoid model. Instead, they evaluated GLI2 protein level (unchanged), GLI3 repressor form (decreased), and GLII mRNA (increased) in GPR161KO organoids. I think they provided an indirect answer to my question, and they added a part in the discussion for the expression level of all three GLI proteins and their downstream effect in SHH pathway.

Overall, I am satisfied with the authors' effort to address my questions. I would accept the manuscript.

Version 2:

Reviewer comments:

Reviewer #1

(Remarks to the Author)
The authors have addressed my concerns. The manuscript is significantly improved.

Open Access This Peer Review File is licensed under a Creative Commons Attribution 4.0 International License, which permits use, sharing, adaptation, distribution and reproduction in any medium or format, as long as you give appropriate credit to the original author(s) and the source, provide a link to the Creative Commons license, and indicate if changes were made.
In cases where reviewers are anonymous, credit should be given to 'Anonymous Referee' and the source.
The images or other third party material in this Peer Review File are included in the article's Creative Commons license, unless indicated otherwise in a credit line to the material. If material is not included in the article's Creative Commons license and your intended use is not permitted by statutory regulation or exceeds the permitted use, you will need to obtain permission directly from the copyright holder.
To view a copy of this license, visit https://creativecommons.org/licenses/by/4.0/

To all reviewers

We would like to sincerely thank all the reviewers for their thoughtful and constructive feedback. We have carefully addressed all comments. We greatly appreciate the time and effort of the reviewers in helping us improve our work.

Response to Reviewer 1's Comments

Reviewer #1 (Remarks to the Author):

In their manuscript, Shimada et al analysed the role of primary cilia in human corticogenesis using cortical organoids. Starting with a proteomic profiling approach, they identified proteins located in primary cilia of cortical radial glial cells. Based on an enrichment of GTPase proteins in the ciliary proteome, they further investigated the role of ARL13B and GPR161. In contrast to inactivating ARL13B which caused a partial ventralisation of cortical organoids, the GPR161 mutation severely ventralized organoids. This ventralisation was rescued by a treatment with forskolin that activates adenylate cyclase and increased cAMP levels or by activating adenylate cyclase specifically in cilia. Finally, they show that removal of GPR161 from cilia is sufficient to induce a partial ventralisation of cortical organoids.

The experiments presented in this manuscript are technically challenging and well suited to address an important question in human corticogenesis, namely the role of primary cilia in forebrain regionalisation. However, there are several shortcomings in the interpretation of data and in the integration of their findings into the current literature.

We would like to thank Reviewer 1 for their thoughtful evaluation of our work and suggestions. The comments have been extremely helpful in improving our manuscript quality and further clarifying the molecular mechanisms. We have addressed each of the points in the responses below.

Major points

1) The authors use the terms brain organoids and cortical organoids interchangeably but these terms denote different types of organoids. Brain organoids comprise several brain regions whereas cortical organoids are models of the cerebral cortex only. This misunderstanding is confusing for the reader and might have also affected the interpretation of ectopic marker expression. For example, NKX2.2, PAX7 and FOXA2 are not expressed in the cortex but in the spinal cord and midbrain. Hence, the ectopic expression of these markers might indicate that mutant organoids are not only ventralised but also caudalised. To test for this possibility, the authors should stain their organoids for FOXG1, a telencephalon marker, and NKX2.1 and GSX2 as ventral telencephalic markers. Their ARL13B bulk mRNA-seq experiment might also show an up-regulation of caudal brain and neural tube markers. The confusing use of the terms brain and cortical organoids might also have affected the interpretation of previous publications. For example, Schembs et al, 2022 used dual Smad inhibition to generate cortical, not brain organoids as stated on page 26. Moreover, this paper extensively analysed necessary and sufficient roles of SHH signalling in dorsal/ventral regionalisation of cortical organoids. The authors should change their interpretation correspondingly.

Response: We thank the reviewer for raising this important point, and we apologize for the confusing terminology in the original submission. We used a commonly used method with dual SMAD inhibitors and a WNT inhibitor to generate cortical organoids, and we originally termed these as cortical organoids; however, as the reviewer pointed, the organoids expressed not only PAX6, but also PAX7 indicating that our control organoids may include non-cortical dorsal progenitors. PAX7 can be detected in the alar plate in the forebrain, as well as spinal cord and midbrain during development, therefore its presence alone does not specify spinal cord identity[1]. To assess whether our organoids have telencephalic identity, we stained organoids for FOXG1 and confirmed that our organoids retain telencephalic identity. Following *ARL13B* deletion, immunohistochemistry analysis showed that FOXG1 expression patterns did not differ, indicating that *ARL13B* KO organoids remain telencephalic identity (new Supplemental Figure 4B, C). Although *FOXG1* mRNA levels were reduced by RNA-seq, FOXG1 protein expression patterns did not differ following *ARL13B* KO. Based on these results, we now refer to these organoids as "telencephalic organoids". We further re-analyzed our bulk RNA-seq data from *ARL13B* KO organoids and found that our organoids are indeed ventralized without obvious caudalization (new Supplemental Figure 5). Our heatmap revealed that the expression patterns of brain region markers (telencephalon (general), dorsal telencephalon, ventral telencephalon, midbrain and hindbrain) from rostral to caudal did not differ between control and *ARL13B* KO samples (new Supplemental Figure 5B). Please note that we plotted the heatmap with $\log_2$ TPM for rostral-caudal analysis, whereas Z-score was used for dorsal/ventral markers and SHH pathway genes (new Supplemental Figure 5). We also performed a heatmap of the brain region markers using Z-scores; however, the rostral-caudal comparison was not clearly visible using this scaling (Please see attached Rebuttal Figure 1). Therefore, we chose to use $\log_2$ TPM for visualization of regional marker expression. In addition, we stained for

NKX2.1 and GSX2 in *ARL13B* KO organoids and confirmed induction of ventral telencephalic markers (new Figure 3E, F and Supplemental Figure 4E, F). Together, these data indicate that *ARL13B* KO organoids are ventralized telencephalic organoids.

We also thank the reviewer for pointing out the study by Schembs et al., 2022. We now re-

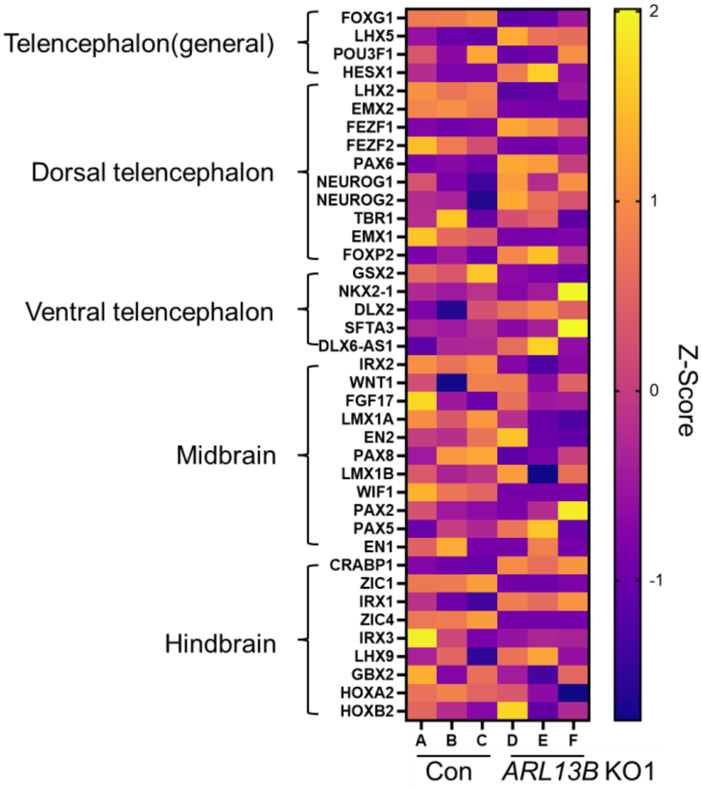

Rebuttal Figure 1. Heatmap of RNA-seq between control and *ARL13B* KO1 organoids. Genes with Z-score were plotted. Please also see Supplemental Figure 5B for comparison.

wrote the introduction to clearly indicate previous works. "Schembs *et al*. demonstrated that loss of Inositol polyphosphate-5-phosphatase E (INPP5E) induced cortical organoids with ventral telencephalic identity and upregulation of SHH signaling"

2) The authors should clearly acknowledge previous work from other labs and their contribution to deciphering the role of cilia in human forebrain regionalisation starting in the introduction to clearly indicate which novel questions their manuscript addresses.

We now re-wrote the introduction to clearly indicate previous works. "Previous studies showed the existence of primary cilia in brain organoids and deletion of ciliary component genes disrupted dorsal/ventral patterning of NPCs [2–5]. Schembs *et al*. demonstrated that loss of Inositol polyphosphate-5-phosphatase E (INPP5E) induced cortical organoids with ventral telencephalic identity with up-regulation of SHH signaling[2]. Others also showed that engineered SHH secretion regulates dorsal/ventral patterning of NPCs in various brain organoid models[6–8]. Consistent with this, a morphogen-gradient patterning study showed that graded SHH is critical for dorsal-ventral patterning of brain organoids[9]. These studies demonstrated the critical role of SHH and primary cilia in NPCs of brain organoids. Despite these advances, little is known about the molecular composition of cilia in human NPCs and whether manipulating components in primary cilia affects the fate of NPCs."

3) In several immunofluorescence stainings of ciliary markers, it is very difficult or impossible to distinguish individual cilia, some images even appear blurry and out of focus (Figures 1K, 2C, 4F, 6G). The authors should present high resolution pictures. Moreover, the authors mainly show single rosettes but not whole organoids to illustrate the expression of dorsal/ventral markers. It remains unclear whether these single rosettes are representative of the expression of these markers in organoids as a whole.

We thank the reviewer for this helpful comment. In the revised manuscript, we replaced the indicated images that clearly resolve individual cilia in new Figure 1K, 2C, 4F and 6G.

We also agree that whole-organoid images are important for interpreting the expression of dorsal/ventral markers. Therefore, we added low-magnification images of whole organoid sections and re-analyzed our data using organoids as the unit of replication. Please see the revised data of Figure 2I, J, Figure 3A-H, Figure 4J-O, Figure 5B-E, H-J, Figure6 H-J, Supplemental Figure 4B, C, E, F, H, I, Supplemental Figure 7A, B, D, E, F, G, Supplemental Figure 8B-D, and Supplemental Figure 10A-C for details.

4) The GLI3 western blots are of poor quality and show multiple unspecific bands. It remains unclear how the 88kDa GLI3 repressor form was identified. Moreover, a 180kDa band for the GLI3 full-length protein was not discernible. Finally, the blots were not quantified and the GLI3 repressor to full length protein ratio which is important for neural tube patterning was not determined.

We thank the reviewer for raising this important point. In the revised manuscript, we have re-performed western blots for GLI3 and showed both GLI3 full length form and GLI3 repressor form (new Figure 4F). We also quantified the GLI3 repressor form divided by either GLI3 full length form or GAPDH (new Figure 4G, H). In both cases, we observed that *GPR161* deletion decreased GLI3 repressor formation, as indicated by previous studies[10–12]. We also performed GLI3 western blotting following forskolin treatment and observed that forskolin treatment rescued the amount of GLI3 repressor formation (Figure 5F and

Supplementaly Figure 8C, D). These data indicate that GPR161 is a critical regulator of GLI3 repressor formation and can be rescued by forskolin treatment.

5) The rescue experiments using the cyto-bPACF198Y-GPR161 KO and ciliary-bPACF198Y-GPR161 KO constructs, presented in Figure 5K and J, lack wildtype and GPR161 KO controls without the bPAC constructs. These controls were, however, included in the experiment using the leaky cyto-bPAC-GPR161 KO and ciliary-bPAC-GPR161 KO constructs (Supplementary Figure 6). Moreover, the authors claim that "the degree of reduction in the number of ventral neural stem cells between cytoplasmic and ciliary bPACF198Y activation before and after the light exposure was similar", but they do not provide a quantification of these ratios. Using the averages presented in Figure 5K, it looks like the cytoplasmic construct has a much stronger effect than the ciliary construct.

We agree with the reviewer that including wildtype and *GPR161* KO controls is critical. We have performed additional experiments for these conditions in dark and light condition. The new figure clearly shows that wild type and *GPR161* KO controls are not affected by light exposure (New Figure 5H-J).

Thank you for asking us regarding the claim that "the degree of reduction in the number of ventral neural stem cells between cytoplasmic and ciliary $bPAC_{F198Y}$ activation before and after the light exposure was similar". Please see the new Figure 5H-J. The new data were quantified with n defined as one organoid (Please see the response to the Comment 3). We did not observe significant differences between cyto-bPAC and cilia-bPAC treatment in the light conditions (new Figure 5H-J). To examine the difference between two treatments, we next analyzed the degree of reduction of cyto-$bPAC_{F198Y}$ and cilia-$bPAC_{F198Y}$ under dark and

light conditions. For this analysis, we first calculated the average values for cyto-bPAC$_{F198Y}$ and cilia-bPAC$_{F198Y}$ in the dark condition, respectively, and then subtracted those values from the corresponding light condition data. As shown in Rebuttal Figure 2, degree of reduction in the number of ventral neural stem/progenitor cells between cytoplasmic and ciliary bPAC$_{F198Y}$ activation before and after the light exposure were not significantly different (Rebuttal Figure 2A, B). In addition, we performed the same analysis using the data from the first submission (n defined as ventricle). There were no significant differences between dark and light conditions (Rebuttal Figure 2C, D). These data suggest that the degree of reduction in the number of ventral neural stem cells between cytoplasmic and ciliary bPACF198Y activation before and after light exposure was not different. However, we agree

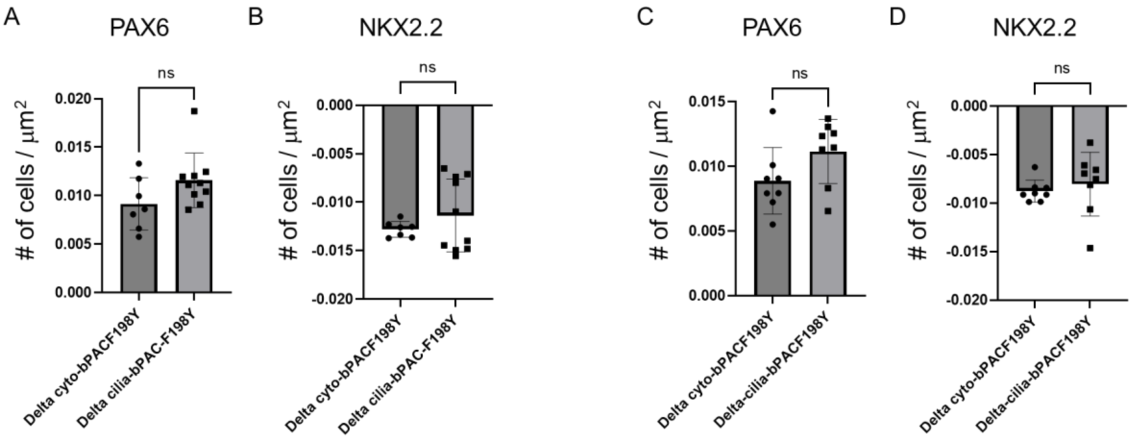

Rebuttal Figure 2. Degree of reduction in the number of ventral neural stem/progenitor cells following cytoplasmic and ciliary bPAC$_{F198Y}$ activation before and after light exposure. (A, B) Differences were calculated by averaging cyto-bPAC$_{F198Y}$ and cilia-bPAC$_{F198Y}$ values under dark conditions, respectively, and then subtracting those values from the corresponding light condition data. (A, B) Each dot represents one organoid; these data are from the revised submission (C, D) Each dot represents one ventricle; these data are from the first submission.

that "similar" might be a too strong word to use, therefore we changed the sentence to, "Since the degree of reduction in the number of ventral NPCs between cytoplasmic and ciliary bPAC$_{F198Y}$ activation before and after the light exposure was not significantly different,

these data indicate that an increase in ciliary cAMP levels following light exposure is sufficient to rescue dorsalization of *GPR161* KO organoids".

6) Figure 6: The low GFP intensity of the GPR161HHC-EGFP construct in cilia without $CuCl_2$ treatment presented in Figure 6F shows that much lower amounts of this fusion protein enter the cilium than the control protein suggesting a defect in ciliary location even before $CuCl_2$ treatment. This is critical for analysing whether ciliary location of GPR161 is required to maintain a cortical fate. On the other hand, the GFP signal in the immunostainings (Figure 6G) appears much higher. There even are plenty of GFP+ but ARL13B- cilia. The authors should also comment why the GPR161-EGFP and GPR161HHC-EGFP fusion proteins restore PAX6 expression irrespective of their ciliary location which contradicts their conclusion.

We thank the reviewer for highlighting this important point. GPR161 is known to function not only in cilia but also outside of cilia in a context-dependent manner[13]. Therefore, it is possible that GPR161 regulates NPC fate through both inside and outside of cilia in human telencephalic organoids. Our data showed that PAX6 expression was rescued by both the GPR161-EGFP and $GPR161_{HHC}$-EGFP models, despite differences in ciliary EGFP intensity. These data suggest that PAX6 expression can be rescued even when ciliary $GPR161_{HHC}$-EGFP is low, and that this may be through combined both intra- and extra-ciliary GPR161 activity. Copper (II) chloride treatment induces exit of $GPR161_{HHC}$-EGFP from cilia, and this change is accompanied by an increase in NKX2.2-positive cells, but not PAX6-positive cells. These results support that ciliary $GPR161_{HHC}$ is sufficient to restrain ventral fate and that loss of ciliary $GPR161_{HHC}$ can promote ventral fate decision of NPCs. However, it was not

sufficient to reduce PAX6-positive cells. Notably, as shown in new Fig. 6J, the increase in NKX2.2 following copper (II) chloride treatment remains lower than in *GPR161* KO organoids, indicating that *GPR161* KO has a stronger effect on NKX2.2 regulation than ciliary exit of $GPR161_{HHC}$-GFP alone. These data also support the possibility that GPR161 function outside of cilia contributes to NKX2.2 regulation. We included the following sentence in the Result section "It is worth noting that the increase in NKX2.2-positive NPCs in *$GPR161_{HHC}$-EGFP GPR161* KO organoids following copper (II) chloride treatment remains lower than in *GPR161* KO organoids, indicating that *GPR161* KO has a stronger effect on NKX2.2 regulation than ciliary exit of $GPR161_{HHC}$-EGFP alone. In contrast, we did not observe a difference of PAX6-positive NPCs in *$GPR161_{HHC}$-EGFP GPR161* KO organoids following copper (II) chloride treatment, indicating that ciliary exit of $GPR161_{HHC}$-EGFP was not sufficient to affect the number of PAX6-positive cells."

We would also like to clarify the relationship between Figure 6F and 6G. Figure 6F represents quantification of ciliary GFP intensity across many cilia in the ventricles of organoids, whereas Figure 6G shows representative immunohistochemistry images. As the reviewer mentioned, the GFP intensity of GPR161-EGFP was much higher than $GPR161_{HHC}$-EGFP in Figure 6F. Because of this difference, most $GPR161_{HHC}$-GFP cilia were difficult to detect using the same microscope setting as GPR161-EGFP. Conversely, when the laser power was increased to visualize $GPR161_{HHC}$-EGFP cilia, most GPR161-EGFP signals became saturated. Therefore, we selected fields in which both GPR161-EGFP cilia and $GPR161_{HHC}$-EGFP cilia could be imaged under the same settings to clarify the location of GPR161 in cilia. As Figure 6F indicates, GFP intensity in the cilia is variable in both

GPR161-GFP and GPR161$_{HHC}$-GFP, therefore a subset of cilia can be imaged with the same setting in new Figure 6G.

We agree that the ciliary GFP signal of GPR161$_{HHC}$ -EGFP is lower than that of GPR161-EGFP in the absence of copper (II) chloride treatment. GPR161$_{HHC}$ is a mutant version of GPR161 (Q193H$^{5.43}$, M287H$^{6.52}$, and A291C$^{6.56}$) in transmembrane helix (TM) 5 and 6. These mutations were introduced to enable coordination chemogenetics for regulating receptor activity. We recently demonstrated a chemogenetic coordination method for metal ion–induced inhibition of adenosine A2A receptor (A$_{2A}$R) activity[14]. We aimed to adapt the A$_{2A}$R strategy to GPR161, and therefore first examined the structural similarity between A$_{2A}$R and GPR161. However, GPR161 lacks some residues suitable for metal ion binding for coordination chemogenetics. To apply coordination chemogenetics to GPR161, we introduced three mutations (Q193H$^{5.43}$, M287H$^{6.52}$, and A291C$^{6.56}$) in TM5 and TM6 and named this construct GPR161$_{HHC}$. These mutations are required for the coordination chemogenetics system. Consistent with our model, copper (II) chloride treatment significantly reduced the cAMP activity (Figure 6D and Supplemental Figure 12A), indicating that constitutive active GPR161$_{HHC}$ activity is reduced following copper (II) chloride treatment. Moreover, copper (II) chloride treatment reduced the ciliary localization of GPR161$_{HHC}$-EGFP (new Figure 6F, G), indicating that GPR161$_{HHC}$-EGFP exits from cilia following copper (II) chloride treatment. Because GPR161 is known to be removed from cilia upon Sonic Hedgehog (SHH) pathway activation, loss of ciliary GPR161 is expected to increase SHH signaling[10–12,15]. In agreement with this idea, following copper (II) chloride treatment, the number of NKX2.2-positive NPCs significantly increased. At the same time, as the reviewer pointed out, GPR161$_{HHC}$-EGFP localization in cilia is impaired without copper (II) chloride

treatment (Figure 6F). Because $GPR161_{HHC}$ contains mutations in TM5 and TM6, it is possible that ciliary localization of $GPR161_{HHC}$ is partially impaired even before copper (II) chloride treatment, or that its exit from cilia is accelerated. TM5 and TM6 are critical domains for Gαs coupling, and therefore there may be altered (such as potentially leakiness) signaling properties even without metal ion binding; however, this is not the case since $GPR161_{HHC}$ overexpression has the same activity of cAMP regulation as GPR161 overexpression (Figure 6D). Additionally, this is supported by the finding that, in *GPR161* KO organoids, expression of *GPR161-EGFP* and *$GPR161_{HHC}$-EGFP* restored the numbers of PAX6 and NKX2.2-positive NPCs to levels that are comparable to control organoids (Figure 6H, J). Our previous work indicates that TULP3 is required for GPR161 trafficking into cilia[16]. The ciliary localization sequence (CLS) in the third intracellular loop (ICL3) lies adjacent to TM5 and TM6, and TM5 and TM6 mutations could alter the local helical context and membrane proximity of ICL3, changing how the CLS is exposed for TULP3 recognition. Moreover, while roles for TM5 and TM6 in ciliary trafficking have not been established, mutations in TM5 and TM6 might affect ciliary targeting indirectly by uncharacterized mechanisms. To determine the mechanism underlying decreased $GPR161_{HHC}$-EGFP in cilia, several follow-up experiments would be needed. First, structural studies of $GPR161_{HHC}$ with TULP3 could test whether their interaction is altered. Second, co-IP followed by mass spectrometry could identify trafficking factors that bind wildtype GPR161 but not $GPR161_{HHC}$. It is possible that unknown proteins bind directly to TM5 and TM6 to promote ciliary targeting. While these experiments would be important, we believe that they are beyond the scope and timeline of the current revision. We therefore added this issue as a limitation in the Discussion, “The mechanism underlying the decreased GFP intensity of $GPR161_{HHC}$ in NPCs remains to be determined. The $GPR161_{HHC}$ mutation may reduce ciliary localization

by impairing trafficking to primary cilia or reducing the retention in primary cilia, resulting in decreased accumulation in the cilia".

Following copper (II) chloride treatment on $GPR161_{HHC}$-EGFP cilia, we observed many GFP puncta around the cilia in the ventricular zones of organoids (new Figure 6G). These GFP puncta were not observed before copper (II) chloride treatment, indicating that they may represent $GPR161_{HHC}$-EGFP that were exited from cilia. To address this, we could perform immunogold staining followed by transmission electron microscope analysis. However, this is technically challenging and would require substantial optimization. Therefore, we will analyze this further in future studies.

7) Throughout the manuscript, the authors appear to have used the number of individual rosettes as n in statistical analyses, but do not state from how many different organoids rosettes were taken. This approach risks pseudo-replication as rosettes within the same organoid are certainly not independent from each other. Instead, the authors should calculate the average from several rosettes within one organoid and repeat this for several organoids. Use the number of organoids as their n number in statistical analyses.

Thank you for raising this important point regarding statistical independence and the risk of pseudo-replication. We agree that ventricular rosettes within the same organoid are not fully independent biological replicates, and that the appropriate unit of replication should be the organoid, not the individual rosette. In the revised manuscript, we re-analyzed our data using organoids as the unit of replication (Figure 2J, Figure 3B, D, F, H, Figure 4K, M, O, Figure 5C-E, H, J, Figure6 H, J, Supplemental Figure 4C, F, I, Supplemental Figure 7B, E, G,

Supplemental Figure 8C, D, and Supplemental Figure 10B, C). We now report the number of organoids for each experiment. The number of organoids is indicated in the figure legends and in the Supplemental Table 4.

Minor points

1) For the reviewers, it would be great to have access to the proteomic data set, i.e. a list of proteins located at the cilium, to judge the quality of the data.

We agree that access to the full proteomic dataset (including the list of cilia-associated proteins) would help reviewers assess the dataset. We have therefore included the data in Supplemental Figure 1 and Supplemental Table 1. The raw data was deposited in the Japan Proteome Standard Repository (JPST003807) and can be accessed via the link below using the access key 3855.

https://repository.jpostdb.org/preview/713049345686749e216040.

2) page 11: Schembs already described cilia in human foetal cortex. Please, cite this paper.

We thank the reviewer for this suggestion. We have now cited this work and added the following sentence to the Introduction: “These data indicate that NPCs in the fetal cortex

have primary cilia, as previously reported in the forebrain of a postconceptional week 8 human embryo and in other mammals".

3) page 11: Based on the sequencing data, the KO1 and KO2 lines in Fig. 2A appear to be different from the KO1 and KO2 lines in Supplementary Figure 2A. Could the authors please clarify this?

We apologize for any confusion. As described in the Materials and Methods, we used two previously reported iPS cell lines "201B7" and "Windy"[17–20]. In the revised manuscript, KO1 and KO2 in the new Figure 2B are *ARL13B* KO lines generated in 201B7 iPS cells, whereas KO1 and KO2 in new Supplemental Figure 2B are *ARL13B* KO lines generated in Windy iPS cells. The iPS cells line names are now indicated in Supplemental Table 4.

4) page 14: Schembs et al already describe GPR161 expression in RGC cilia. Please, cite this paper,

We thank the reviewer for pointing this out. We have now cited the work and added the sentence below in the results. "GPR161 localizes to primary cilia in both mouse and human telencephalic organoids, and negatively regulates the SHH signaling pathway".

5) page 15: Spina bifida patients cannot indicate the importance of a gene in brain development, as the disorder affects the spinal cord not the brain.

We appreciate this clarification and agree that the original wording was not accurate. We have revised the sentence in the Result section: "*GPR161* deletion induces open cranial and caudal neural tubes in mice and dominant-negative variants of *GPR161* have been identified in spina bifida patients, indicating the importance of GPR161 during brain development"

6) page 24: The authors claim that "this rescue was partly mediated by GLI3 repressor formation" but they do not perform experiments to test the role of reduced GLI3 repressor levels. The GLI3 western blot only allows to correlate GLI3 repressor levels with the ventralisation phenotype.

We agree with the reviewer that our previous wording was too strong. To address this, we have revised the text as follows: "This rescue by forskolin was accompanied by increased levels of GLI3 repressor formation; however, assessment of the role of the GLI3 repressor in the GPR161 KO background requires careful analysis in future studies

Reviewer #2 (Remarks to the Author):

The study by Shimada et al. demonstrates that primary cilia act as precise signaling hubs during early brain patterning, where the ARL13B–GPR161–cAMP axis finely regulates dorsal versus ventral fate specification. Furthermore, it highlights the potential of spatially targeted interventions, such as optogenetics and chemogenetics, to modulate human neural stem cell lineage commitment.

The role of GPR161 as a key negative regulator of the SHH pathway has been well established in several studies. GPR161 functions as an essential "gatekeeper" of SHH signaling by localizing to the cilium, driving local cAMP production, and anchoring PKA to ensure that Gli transcription factors are processed into their repressor forms. Loss of GPR161 function removes this regulatory brake, leading to unchecked Gli activator formation and resulting in widespread ventralization or tissue overgrowth during development.

The authors present an elegant study utilizing a CRISPR-based GPR161 knockout model, which resulted in the elimination of dorsal ($PAX6^+$) progenitors and the induction of ventral markers (NKX2.2, FOXA2), thereby confirming the critical role of GPR161 in SHH pathway regulation and dorsal–ventral fate specification. Through optogenetic and chemogenetic approaches, they further demonstrate the importance of ciliary cAMP levels and the dynamic localization of GPR161 in determining neural stem cell fate.

One of the findings in this study appears to contradict the current understanding of SHH pathway regulation in primary cilia. The authors report that ARL13B knockout causes

abnormal ciliary morphology, mislocalization of GPR161, and enhanced SHH signaling, leading to partial ventralization of neural stem cells even under dorsal-patterning conditions. Previous studies, however, suggest that loss of ARL13B does not hyperactivate SHH signaling in human cells. Instead, ARL13B is considered essential downstream of activated SMO for generating full Gli activator (GliA) output. In human medulloblastoma cell lines, ARL13B knockdown reduces GLI1 and PTCH1 transcription in response to SHH ligand, SMO agonists, or activating SMO mutations. While the authors attribute the observed ventralization to reduced SHH repression via mislocalized GPR161 and decreased GliR, the status of GliA is not addressed. Considering that SHH pathway activation depends on the GliA/GliR balance, evaluating GliA levels would strengthen the mechanistic explanation of their findings. A luciferase-based SHH activity assay would further support their conclusions.
Minor issues: The resolution of Figures 2C and 4F is very low, making it difficult to discern ciliary structures. Given that Figures 1P and 4C demonstrate the authors' ability to produce high-resolution ciliary images, improving the resolution of Figures 2C and 4F is strongly recommended.

Overall, this study provides a compelling demonstration of the primary cilia–GPR161–cAMP axis in regulating dorsal versus ventral fate commitment. Addressing the issues outlined above would further strengthen the manuscript and make it a valuable contribution worthy of publication in Nature Communications.

We would like to thank Reviewer 2 for their positive feedback on our work. Their comments have been invaluable in enhancing the quality of the manuscript. We have addressed each of their points in the responses below.

Thank you for this important comment. We agree that ARL13B loss does not simply hyperactivate SHH signaling. ARL13B is a critical signaling component in the primary cilia for SHH regulation. *Arl13b*^{hnn} mutant and *Arl13b* KO embryos show increased ventral NPCs in the neural tube with low-level Gli activator activity[21,22]. Moreover, in SHH medulloblastoma model, *Arl13b* deletion inhibits tumor growth by preventing SHH activation[23]. These data indicate that Arl13b is a fine-tuning regulator for sonic hedgehog activity in cilia.

We agree that evaluating the GLIA/GLIR balance strengthens the mechanistic insight of our findings. Because GLI2 is a GLI activator, we first aimed to analyze GLI2 in *ARL13B* KO organoids. However, in 3D organoids, *ARL13B* KO primary cilia could not be reliably stained with commonly used ciliary antibodies (CFAP300, IFT88, INPP5E, poly-E, and acetylated α-tubulin). In addition, because transient gene expression is technically challenging in 3D telencephalic organoids, we were unable to perform a GLI binding domain luciferase assay in this model. Although we observed *GPR161* mislocalization in 2D NPCs derived from *ARL13B* KO iPS cells, we considered cilia in the 3D organoids are more suitable for the analysis of cilia-dependent NPC fate decisions, as the organoid models recapitulate *in vivo* tissue architecture.

To obtain GLI activator/GLI repressor balance in a 3D context despite these technical limitations, we focused on *GPR161* KO organoids. A recent study showed increased ciliary GLI2 in *INPP5E* KO organoids[2]. We therefore stained and quantified GLI2 in the cilia of *GPR161* KO organoids (new Supplemental Figure 6E, F), but did not detect increased GLI2 in *GPR161* KO organoids as compared to control telencephalic organoids. This result suggests that increased ciliary GLI2 may not be a dominant mechanism in *GPR161* KO model. Consistent with this, Brooks and Mukhopadhyay *et al* showed that GLI2 is dispensable for rostral neural tube development in *GPR161* KO mice[24]. GLI3 plays a

significant role in rostral neural tube rescue and both GLI2/GLI3 contribute in the midline-caudal neural tube rescue[24]. We also found that ciliary localization of SMO was increased in *GPR161* KO organoids (new Supplemental Figure 6G, H). Previous paper showed that activated SMO can suppress PKA through its C-terminal PKA inhibitory motif[25]. In addition, *GLI1* mRNA was upregulated (Figure 4I), and GLI3 repressor formation was decreased (Figure 4F–H). Recent study showed that GLI1 functions as a transcription factor after ciliary localization and subsequent nuclear translocation, where it binds the SHH target genes[26]. In agreement with our study, previous study showed that *GLI3* deletion is critical for dorsal/ventral fate decision of NPCs in unguided brain organoids[8]. Therefore, our data support a shift of the GLI activator/repressor balance toward GLI activator output in *GPR161* KO organoids. We re-wrote the following paragraph in the discussion. "SHH signaling determines dorsal/ventral fate of the neural tube in various animal model[24,27,28]. Upregulation of *GLI1* in both *ARL13B* and *GPR161* KO organoids suggests that SHH signaling contributes to ventralization. GLI1 acts as the amplifier of SHH signaling pathway. A recent study showed that GLI1 also functions as a transcription factor after ciliary localization and subsequent nuclear translocation, where it binds SHH target genes[26]. SMO also accumulated in the cilia of *GPR161* KO organoids, indicating activation of SMO-mediated inhibition of PKA[29]. Indeed, GLI3 repressor formation was decreased in *GPR161* KO organoids. In agreement with our study, a previous study showed that GLI3 deletion is critical for dorsal/ventral fate decisions of NPCs in unguided brain organoids[8]. These data indicate that reduced GLI3 repressor is a major downstream effect in *GPR161* KO organoids. However, the precise roles of full-length GLI3 and GLI3 repressor in dorsal/ventral fate decisions remain to be examined. Notably, we did not observe increased GLI2 levels in the cilia of *GPR161* KO organoids as compared to control telencephalic organoids, suggesting that GLI2 may not be critical in this

context. Interestingly, Brooks *et al.* recently showed that Gli2 is dispensable for rostral neural tube development in *Gpr161* KO mice [24]. They reported that Gli3 is critical for rostral neural tube closure, and both Gli2 and Gli3 are critical for midline-caudal neural tube closure in *Gpr161* KO mice[24]. Since GLI1 was increased and GLI3 repressor formation was decreased in *GPR161* KO organoids, the GLI activator/repressor balance is shifted toward activator function in our organoid model."

We thank the reviewer for this helpful comment. In the revised manuscript, we replaced the indicated images that clearly resolve individual cilia in new Figure 2C and 4F.

References

1. Namm, A., Arend, A., Suutre, S. & Aunapuu, M. Expression of pax6 and pax7 proteins during the central nervous system development in human embryos. *International Journal of Morphology* **38**, (2020).
2. Schembs, L. *et al.* The ciliary gene INPP5E confers dorsal telencephalic identity to human cortical organoids by negatively regulating Sonic hedgehog signaling. *Cell Rep.* **39**, (2022).
3. Gabriel, E. *et al.* CPAP promotes timely cilium disassembly to maintain neural progenitor pool. *EMBO J.* **35**, 803–819 (2016).
4. Eiraku, M. *et al.* Self-Organized Formation of Polarized Cortical Tissues from ESCs and Its Active Manipulation by Extrinsic Signals. *Cell Stem Cell* **3**, (2008).
5. Wiegering, A. *et al.* A differential requirement for ciliary transition zone proteins in human and mouse neural progenitor fate specification. *Nature Communications* **16**, (2025).
6. Cederquist, G. Y. *et al.* Specification of positional identity in forebrain organoids. *Nat. Biotechnol.* **37**, (2019).

7. De Santis, R., Etoc, F., Rosado-Olivieri, E. A. & Brivanlou, A. H. Self-organization of human dorsal-ventral forebrain structures by light induced SHH. *Nat. Commun.* **12**, (2021).

8. Fleck, J. S. *et al.* Inferring and perturbing cell fate regulomes in human brain organoids. *Nature* **621**, 365–372 (2023).

9. Scuderi, S. *et al.* Specification of human brain regions with orthogonal gradients of WNT and SHH in organoids reveals patterning variations across cell lines. *Cell Stem Cell* **32**, (2025).

10. Shimada, I. S. *et al.* Derepression of sonic hedgehog signaling upon Gpr161 deletion unravels forebrain and ventricular abnormalities. *Dev. Biol.* **450**, 47–62 (2019).

11. Mukhopadhyay, S. *et al.* The ciliary G-protein-coupled receptor Gpr161 negatively regulates the sonic hedgehog pathway via cAMP signaling. *Cell* **152**, 210–223 (2013).

12. Shimada, I. S. *et al.* Basal Suppression of the Sonic Hedgehog Pathway by the G-Protein-Coupled Receptor Gpr161 Restricts Medulloblastoma Pathogenesis. *Cell Rep.* **22**, (2018).

13. Hwang, S. H., Somatilaka, B. N., White, K. & Mukhopadhyay, S. Ciliary and extraciliary gpr161 pools repress hedgehog signaling in a tissue-specific manner. *Elife* **10**, (2021).

14. Doura, T. *et al.* A chemogenetic strategy for inhibiting G protein-coupled receptor activity using metal coordination. Preprint at https://doi.org/10.26434/chemrxiv-2025-1zq06 (2025).

15. Pal, K. *et al.* Smoothened determines β-arrestin-mediated removal of the G protein-coupled receptor Gpr161 from the primary cilium. *Journal of Cell Biology* **212**, (2016).

16. Badgandi, H. B., Hwang, S.-H., Shimada, I. S., Loriot, E. & Mukhopadhyay, S. Tubby family proteins are adapters for ciliary trafficking of integral membrane proteins. *J. Cell Biol.* **216**, (2017).

17. Kondo, Y. *et al.* Histone deacetylase inhibitor valproic acid promotes the differentiation of human induced pluripotent stem cells into hepatocyte-like cells. *PLoS One* **9**, (2014).

18. Iwao, T. *et al.* Differentiation of human induced pluripotent stem cells into functional enterocyte-like cells using a simple method. *Drug Metab. Pharmacokinet.* **29**, 44–51 (2014).

19. Nishino, K. *et al.* Defining hypo-methylated regions of stem cell-specific promoters in human iPS cells derived from extra-embryonic amnions and lung fibroblasts. *PLoS One* **5**, (2010).

20. Nakamura, Y. *et al.* Biallelic null variants in PNPLA8 cause microcephaly by reducing the number of basal radial glia. *Brain* **147**, (2024).

21. Caspary, T., Larkins, C. E. & Anderson, K. V. The Graded Response to Sonic Hedgehog Depends on Cilia Architecture. *Dev. Cell* **12**, 767–778 (2007).

22. Su, C. Y., Bay, S. N., Mariani, L. E., Hillman, M. J. & Caspary, T. Temporal deletion of Arl13b reveals that a mispatterned neural tube corrects cell fate over time. *Development (Cambridge)* **139**, (2012).

23. Bay, S. N., Long, A. B. & Caspary, T. Disruption of the ciliary GTPase Arl13b suppresses Sonic hedgehog overactivation and inhibits medulloblastoma formation. *Proc. Natl. Acad. Sci. U. S. A.* **115**, 1570–1575 (2018).

24. Brooks, E. R., Hwang, S. H., White, K. A. & Mukhopadhyay, S. GPR161-GLI3 repressor signaling at cilia directs apical constriction and cell fate during cranial neural tube closure. *Development* **152**, (2025).

25. Happ, J. T. *et al.* A PKA inhibitor motif within SMOOTHENED controls Hedgehog signal transduction. *Nat. Struct. Mol. Biol.* **29**, (2022).

26. Dear, H. S. *et al.* GliFHV mice: a tool to investigate GLI processing and localization. *Development* **152**, (2025).

27. Legué, E. & Liem, K. F. Mutations in Ciliary Trafficking Genes affect Sonic Hedgehog-dependent Neural Tube Patterning Differentially along the Anterior–Posterior Axis. *Neuroscience* **450**, 3–14 (2020).

28. Dessaud, E., McMahon, A. P. & Briscoe, J. Pattern formation in the vertebrate neural tube: A sonic hedgehog morphogen-regulated transcriptional network. *Development* vol. 135 Preprint at https://doi.org/10.1242/dev.009324 (2008).

29. Walker, M. F. *et al.* GRK2 kinases in the primary cilium initiate SMOOTHENED-PKA signaling in the Hedgehog cascade. *PLoS Biol.* **22**, (2024).

We would like to sincerely thank all the reviewers for their thoughtful and constructive feedback. We have carefully addressed all comments. We greatly appreciate the time and effort of the reviewers in helping us improve our work.

Reviewer #1 (Remarks to the Author):

The authors addressed most of my concerns and have certainly improved the quality of the manuscript, but there are still severe short comings with respect to the ventralisation of the organoids. On the positive side, the authors present new data on the expression of the ventral telencephalic markers NKX2.1 and GSX2 for ARL13B mutant organoids in the revised manuscript, however, the expression of these markers was not investigated in either the GPR161 mutant organoids or in the two rescue experiments. Their central claim that cilia mediated signalling is required to inhibit ventralisation under these conditions just rests on the ectopic expression of NKX2.2. In the mouse, this gene is not expressed in the ventral telencephalon and, to my knowledge, its expression in the human foetal telencephalon has not been described. In fact, their own transcriptomic data showed hardly any NKX2.2 expression (1 transcript on average per organoid) in control telencephalic organoids whereas the well-established ventral telencephalic markers GSX2 and NKX2.1 have 663 and 997 transcripts on average, respectively. Hence, to support their key claim, the authors must show that NKX2.2 is indeed expressed in human foetal ventral telencephalon using immunostainings or cite relevant studies. If NKX2.2 is not expressed in this tissue, they need to analyse NKX2.1 and/or GSX2 expression in GPR161 mutant organoids and in their rescue experiments instead as they have done for ARL13B mutant organoids. Otherwise, they can only assert that NKX2.2 ectopic expression was rescued. Claiming a ventralisation of the telencephalic organoids and its rescue would be an unjustified overstatement.

We thank the reviewer for this important comment. We agree that NKX2.2 is not canonical marker of ventral telencephalon, and that NKX2.2 expression alone is not sufficient to support a claim of ventralization of neural stem/progenitor cells (NPCs). The organoids may exhibit gene expression patterns distinct from those of *in vivo* models; therefore, we may have observed ectopic expression of non-canonical genes in our organoids. At the same time, we think it is important to interpret NKX2.2 in its broader developmental context. In mouse, Flames *et al*. showed that *Nkx2.2* can be detected at low levels in the medial ganglionic eminence (MGE) by *in situ* hybridization (Flames et al., 2007). In human, Hansen *et al*. reported that NKX2.2-positive cells can be visualized very sparsely by immunohistochemistry in the MGE (Hansen et al., 2013). In addition, Braun *et al*. showed weak *NKX2.2* detection in the subpallium of 5 post conceptional week (pcw) human brain using the multiplexed RNA enhanced electric fluorescence *in situ* hybridization(Braun et al., 2023). These

data suggest that, although NKX2.2 is very weak and sparse, NKX2.2 expression can be detected in the ventral telencephalon in mice and human. We agree that NKX2.2 alone is not sufficient to define ventralization; however, NKX2.2 can serve as supporting evidence for ventralization.

To address the reviewer's concern directly, we performed immunostaining for NKX2.1 and GSX2 in *GPR161* KO organoids. We found that the number of NKX2.1-positive cells was increased in *GPR161* KO organoids, and that this increase was reduced by forskolin treatment and optogenetics (Figures 5D, 5E, 5I and 5J; and Supplemental Figures 9D and 12E). In addition, chemogenetics experiments showed that exit of GPR161$_{HHC}$ from primary cilia was sufficient to increase NKX2.1-positive NPCs. (Figures 6I and 6J; and Supplemental Figures 14C). In contrast, we did not observe a clear increase in GSX2-positive cells following *GPR161* deletion (Supplemental Figures 8A and 8B). These results suggest that *GPR161* deletion induces ventralization of organoids based on both NKX2.1 and NKX2.2. These data indicate that *GPR161* and *ARL13B* deletion induced different ventral patterning outcomes, which may be due to the fact that ARL13B has functions not only in SHH signaling, but also in other ciliary signaling.

We have revised the manuscript to include NKX2.1 and GSX2 and have carefully worded the text to avoid overinterpretation. We discussed ventralization of ARL13B and GPR161 in the discussion. "Of note, the *GPR161* deletion phenotype appears different from the phenotype induced by *ARL13B* deletion in our organoids. *ARL13B* deletion induced organoids with NKX2.1, NKX2.2 and GSX2, suggesting a ventral telencephalon identity with LGE/MGE-like features, although NKX2.2 is not a canonical marker of ventral telencephalon. NKX2.2 is a commonly used marker for the ventralization of neural tube, and hypothalamus region, with limited expression in the ventral telencephalon(Braun et al., 2023; Flames et al., 2007; Hansen et al., 2013). On the other hand, *GPR161* deletion induced organoids with NKX2.1, NKX2.2 and FOXA2, but not GSX2, suggesting a ventral forebrain pattern with hypothalamus-like features. The interpretation is broadly consistent with the tripartite hypothalamus model, in which the anterior tuberal hypothalamus occupies a ventral position relative to the telencephalon(Manning et al., 2025). It is also important to note that organoids may exhibit gene expression patterns distinct from those of *in vivo* models; therefore, we may have observed ectopic expression of non-canonical genes in the organoids. Nevertheless, compared to control organoids, which express PAX6 but not NKX2.1, NKX2.2, GSX2 or FOXA2, deletion of *ARL13B* or *GPR161* induced ventralization-associated changes in NPC markers under a commonly used protocol for telencephalic organoids."

We thank the reviewer again for this comment, which helped us improve both the interpretation of the data and the quality of the manuscript.

Minor points:

1) p13: FOXG1 is expressed in the telencephalon but not in the diencephalon. Hence, it is a telencephalic marker, not a forebrain marker. This may need to be amended throughout the manuscript.

We thank the reviewer for this correction. We agree that FOXG1 is a telencephalic marker, and we have corrected this description throughout the manuscript.

2) Figures 5D and E: the graphs have been swapped in the revised manuscript compared to the original. The authors need to check this swap.

We thank the reviewer for pointing this out. The graphs in Figures 5D and 5E had indeed been swapped, and this has now been corrected.

3)Figure 5I: Enlarge the organoid image that they cover the whole space.

We thank the reviewer for this suggestion. The organoid image in Figure 5I has been enlarged.

Reviewer #2 (Remarks to the Author):

For the 2 key questions I raised

1. The authors' data suggested that ARL13B knockout causes enhanced SHH signaling. How would they account for the contradiction to previous studies suggesting ARL13B knockdown reduces SHH pathway responses?

They couldn't provide an explanation for that but in the manuscript, they cite prior work saying ARL13B deletion causes GPR161 mislocalization and impairs responsiveness to SHH signaling in primary cilia, therefore acknowledging literature where ARL13B loss is also linked to reduced/altered SHH responsiveness. The combination of their data and previous finding suggested that ARL13B mechanism is context-dependent in ciliary signaling.

2. The GLI-activator/repressor balance.

The authors pointed out technical difficulties that ARL13B KO primary cilia could not be reliably stained with commonly used ciliary antibodies, and also, transient gene expression is technically challenging in 3D telencephalic organoids, they were unable to perform a GLI binding domain luciferase assay to evaluate the level of GLI activation in their organoid model. Instead, they evaluated GLI2 protein level (unchanged), GLI3 repressor form (decreased), and GLII mRNA (increased) in GPR161KO organoids. I think they provided an indirect answer to my question, and they added a part in the discussion for the expression level of all three GLI proteins and their downstream effect in SHH pathway.

Overall, I am satisfied with the authors' effort to address my questions. I would accept the manuscript.

We thank the reviewer for the careful evaluation of our revised manuscript and for the positive assessment of our efforts to address the previous comments. We appreciate for the reviewer's support of the manuscript.

References

Braun, E., Danan-Gotthold, M., Borm, L. E., Lee, K. W., Vinsland, E., Lönnerberg, P., Hu, L., Li, X., He, X., Andrusivová, Ž., Lundeberg, J., Barker, R. A., Arenas, E., Sundström, E., & Linnarsson, S. (2023). Comprehensive cell atlas of the first-trimester developing human brain. *Science*, *382*(6667). https://doi.org/10.1126/science.adf1226

Flames, N., Pla, R., Gelman, D. M., Rubenstein, J. L. R., Puelles, L., & Marín, O. (2007). Delineation of multiple subpallial progenitor domains by the combinatorial expression of transcriptional codes. *Journal of Neuroscience*, *27*(36). https://doi.org/10.1523/JNEUROSCI.2750-07.2007

Hansen, D. V., Lui, J. H., Flandin, P., Yoshikawa, K., Rubenstein, J. L., Alvarez-Buylla, A., & Kriegstein, A. R. (2013). Non-epithelial stem cells and cortical interneuron production in the human ganglionic eminences. *Nature Neuroscience*, *16*(11). https://doi.org/10.1038/nn.3541

Manning, E., Chinnaiya, K., Furley, C., Kim, D. W., Blackshaw, S., Placzek, M., & Place, E. (2025). Resolving forebrain developmental organisation by analysis of differential growth patterns. *Nature Communications*. https://doi.org/10.1038/s41467-025-67623-6
